# Double Orthogonal Factorization Systems

**DOI:** 10.1007/s10485-026-09891-x

**Published:** 2026-11-12

**Authors:** C. B. Aberlé, Elena Caviglia, Matthew Kukla, Ruben Maldonado, Luca Mesiti, Dorette Pronk, Tanjona Ralaivaosaona

**Affiliations:** 1 Carnegie-Mellon University, Pittsburgh, USA; 2 Stellenbosch University, Stellenbosch, South Africa; 3 National Institute for Theoretical and Computational Sciences, Stellenbosch, South Africa; 4 The George Washington University, Washington, D.C., USA; 5 Universidad Nacional Autónoma de México, Mexico City, México; 6 Dalhousie University, Halifax, Canada; 7 Leiden Institute of Advanced Computer Science, Leiden, The Netherlands

**Keywords:** Double categories, Double fibrations, Factorization systems, Monads, Internal categories, 18N10, 18A32, 18B10, 18C15

## Abstract

We define strict and lax orthogonal factorization systems on double categories. These consist of an orthogonal factorization system on arrows and one on double cells that are compatible with each other. Our definitions are motivated by several explicit examples, including factorization systems on double categories of spans, relations and bimodules. We then prove monadicity results for orthogonal factorization systems on double categories in order to justify our definitions. For fibrant double categories we discuss the structure of the double orthogonal factorization systems that have a given orthogonal factorization system on the arrows in common. Finally, we study the interaction of orthogonal factorization systems on double categories with double fibrations.

## Introduction

The notion of factorizing arrows into arrows from certain left and right subclasses has played an important role in category theory since its very beginning [1]. Orthogonal factorization systems (OFS) for ordinary categories were officially introduced by Isbell [2], Freyd and Kelly [3], and Bousfield [4]. An orthogonal factorization system provides a category with a notion of *image* along its arrows, generalizing the usual image factorization on categories such as $\textbf{Set}$. For instance, various factorization systems on the category of categories give rise to the following images for functors: the replete image, the essential image and the full image. This concept of image is integral to the foundational structure of homological algebra. More generally, orthogonal factorization systems reveal important properties of the structure of a category, and may assist in defining a functor on a category. As was shown in [5], an orthogonal factorization system can be viewed as a type of distributive law for the interaction between the two classes of arrows: if one can compose the arrows in each class and has a way of re-factorizing a composition of an arrow in the left class after an arrow in the right class, one has a composition law for the whole category.

A related aspect of orthogonal factorization systems is their interaction with fibrations between categories. Each fibration gives rise to what has been called a *cartesian factorization system* on its domain: the left class consists of the vertical arrows in the fibration (and satisfies the 3-for-2 property) and the right class consists of the cartesian arrows. This result has been known as folklore; it was mentioned without proof in [6] and was proved in a broader context in Theorem 3.9 of [7]. It can be generalized to show that there is a canonical way to lift any orthogonal factorization system in the base of a fibration to an orthogonal factorization system in the total category.

With the recent introduction of the notion of a fibration between double categories [8], we believe it is time to develop an analogous notion of orthogonal factorization system for double categories that interacts well with the double fibrations.

In this paper we will study a notion of orthogonal factorization system for double categories that generalizes that for ordinary categories. We call these *double orthogonal factorization systems* (DOFS). We will show how a number of well-known orthogonal factorization systems can be extended to double orthogonal factorization systems, often in more than one way. This notion of double orthogonal factorization system was also introduced in [9], where the interaction of these factorization systems with monads inside a double category was studied for its applications to game semantics. However, none of the aspects that we address in the current paper were considered in that paper. In [10], Paré introduced a different, more 2-dimensional factorization system of double cells that suggests further expansions of this notion.

Motivated by examples of factorization systems on the double categories $\mathbb {S}\textrm{q} (\mathcal {C})$ of squares in a category $\mathcal {C}$, $\mathbb {S}\textrm{pan}(\mathcal {C})$ of spans in a category with pullbacks, and $\mathbb {R}\textrm{el}(\mathcal {C})$ of relations in a regular category $\mathcal {C}$, we see how an orthogonal factorization system on $\mathcal {C}$ can be lifted to an orthogonal factorization system on the category of proarrows and double cells of the double category that is suitably compatible with the factorization systems on the arrows. (We will show in later sections that there is often more than one way to lift an OFS on the arrows of a double category to one on the category of proarrows and double cells.)

This leads us to our internal definition of a double orthogonal factorization system (DOFS): a pseudo-category object in a suitably enhanced 2-category of orthogonal factorization systems with a choice of factorizations. We require that the source, target and units functors of these category objects preserve the factorizations, but the composition functor in the category diagram can be more ‘loose’. If the composition functor preserves both classes, we call this a pseudo-DOFS; if it also preserve the chosen factorizations we call it a strict DOFS; if it preserves only the right class we call it a lax DOFS, and if it preserve only the left class we call it a colax DOFS.

Note the similarity with the notion of double fibration [8]. For double fibrations one takes internal pseudo-categories in the category of fibrations with a chosen cleavage but whose arrows only preserve cartesian arrows; however, one requires that the source and target functors in a double fibration diagram preserve the chosen cleavages. The reason for this restriction is the same as it is for us: we require the existence of the pullback of the source along the target as an object in our category. For the DOFS we require more strictness for the units functor, primarily because we have this in all examples we have considered and it allows us to give a description of a DOFS that is phrased exclusively in terms of the double cells of the double category (cf. Proposition 3.17). For some purposes it may be convenient to work with a units functor that only preserves the chosen left and right classes but does not preserve chosen factorizations. Almost all of our theory goes through for this case and we have added comments throughout the paper providing the parallel results for this case.

Classically, a good way to capture and understand orthogonal factorization systems and morphisms between them is in terms of algebras for a particular 2-monad. The first result in this direction was published by Coppey [11] showing that strict factorization systems can be viewed as strict algebras for the squaring 2-monad. The full result characterizing orthogonal factorization systems with a chosen factorization as the pseudo-algebras for this 2-monad was established by Korostenski and Tholen in [12]. This result leads us to four natural notions of morphism between categories with an OFS: the strict algebra maps preserve the chosen factorizations, the pseudo-maps preserve both classes of morphisms, the lax maps preserve the right class and the colax maps preserve the left class. (Note that this could also be obtained from a result in Sect. 5 of [13].) This provides us with four 2-categories in which to take internal pseudo-categories in order to define a corresponding notion of DOFS. We illustrate this with several examples in each category of algebras.

However, there is a different way to approach the generalization from OFSs to DOFSs. In Theorem 4.3 we establish a monadicity result that intuitively says that suitable 2-monads on a 2-category give rise to a 2-monad on the 2-category of internal pseudo-categories with internal (pseudo or oplax) functors as arrows, and level-wise (also called, internal) transformations as 2-cells, with the property that the 2-category of normal pseudo-algebras for the 2-monad on the internal categories is isomorphic to the 2-category of internal categories internal to the enhanced 2-category of normal pseudo-algebras of the original 2-monad.

When applied to the 2-category of categories, this general monadicity theorem allows us to view our various flavours of DOFS as normal pseudo-algebras for the 2-monads on various 2-categories of double categories defined by sending a double category $\mathbb {D}$ to the double category $\mathbb {D}^\rightarrow$ obtained by applying the squaring monad on  component-wise to $\mathbb {D}$ (i.e., to each of the categories $D_0$ and $D_1$). As we show in Construction 4.6, $\mathbb {D}^\rightarrow$ is equivalently given by the exponential of $\mathbb {D}$ to the double category which has a single non-trivial horizontal (i.e. tight) arrow and only has identities as proarrows and double cells. Exponentials of double categories are described and explicitly constructed in [14, Page 187].

This lets us establish the correct notions of arrows between double categories with a DOFS. The natural notion of 2-cell coming from the algebra perspective is that of an internal, or levelwise, transformation. These are the transformations with components that are arrows in the strict direction of the double category. We simply take all such transformations between the appropriate (lax, colax or pseudo) morphisms. However, we can also add transformations in the proarrow direction: these correspond to suitable functors from the object category of the domain to the arrow category of the codomain, in a manner compatible with the OFS structures on the underlying categories.

We end the paper with a large variety of examples. We show how extensions and restrictions in fibrant double categories allow us to lift each orthogonal factorization system on its category of objects and arrows to two double orthogonal factorization systems. We also show that these are initial and terminal among all possible liftings, and illustrate this in the double categories of spans, relations and profunctors. In our final section, we discuss the relationship between double orthogonal factorization systems and double fibrations and show how a double factorization system on the base can be lifted to one on the total double category. In particular, for the double category of spans these methods provide us with four distinct double orthogonal factorization systems extending the epi-mono factorization system on sets.

### Outline of the Paper

Section 2 recalls the definition of orthogonal factorization system for ordinary categories, as well as their monadicity result. We then apply this monadicity result to discuss strict, pseudo, lax and colax arrows and 2-cells between orthogonal factorization systems. This provides us with various 2-categories of OFSs. We also establish a useful result on pullbacks of OFSs.

In Sect. 3, we begin by reviewing the definition and introduce our terminology for double categories. We then present our motivating examples (the double categories of squares, of relations and of spans), followed by the internal definition of a double orthogonal factorization system (as a suitable internal category in one of the 2-categories of OFSs). We then present an equivalent characterization in terms of cells, and conclude the section with examples of DOFSs related to double categories of quintets.

Section 4 establishes the monadicity of double categories equipped with a double orthogonal factorization systems as algebras for an $\mathcal {F}$-monad. This sheds light on the various flavours (strict, pseudo, lax and colax) of double orthogonal factorization systems, naturally forming various 2-categories of DOFSs. However, note that in the larger world of double categories there are more arrows and 2-cells available, placing all of these 2-categories inside a larger double category.

In Sect. 5 we first prove some general results about DOFSs on fibrant double categories. We then explore a list of examples (modules, profunctors, quantale-valued relations, and spans) of fibrant double categories with multiple DOFSs lifting a common OFS on the arrows.

Section 6 discusses the lifting of double orthogonal factorization systems along double fibrations. We show many examples, including explicit descriptions of (pseudo-vertical, cartesian) factorization systems and the morphisms in the category of DOFSs (of various flavours) that form liftings of a common OFS.

### Notation

Given a 2-category , we will write  (respectively, , ) for the 2-category of normal (i.e., unitary) pseudo-categories internal to , with normal (i.e., unitary) internal pseudo (respectively, normal lax, normal oplax) functors and internal, also called levelwise, natural transformations; we will review these concepts in detail in Sect. 3.1.

Given a monad $T:\mathcal {C}\rightarrow \mathcal {C}$ on a category $\mathcal {C}$, we will write  for the category of *T*-algebras and algebra morphisms.

Given a 2-monad  on a 2-category , we will denote by  (respectively, , ) the 2-category of normal pseudo-algebras with strict (respectively, pseudo, lax) morphisms and 2-cells (i.e., *T*-transformations).

## Categories of Orthogonal Factorization Systems

In this section, we first recall ordinary orthogonal factorization systems and their monadicity over the 2-category  of categories. We then extract from this monadicity result three flavours of morphisms between categories equipped with orthogonal factorization systems. For brevity, we refer to a morphism of such categories simply as a *morphism of orthogonal factorization systems*, or, morphism of OFSs. Finally, we study the corresponding 2-categories of orthogonal factorization systems.

### Definition 2.1

( [3]) Let  be a category. An *orthogonal factorization system* on  consists of two classes of morphisms  and  such that:

- The classes  and  are closed under composition, and contain all isomorphisms.
- Every morphism f:A→B in  can be written as $f=r_f \circ \ell _f$ where  and , giving commutativity of the following diagram:

**Figure:**
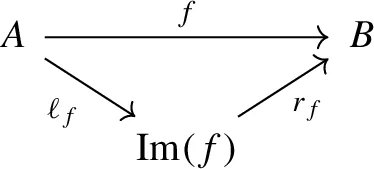
- For any commutative square

**Figure:**
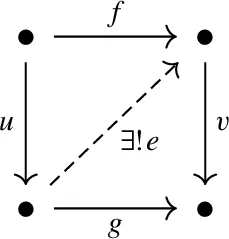

where , there exists a unique *e* such that g=v∘e and f=e∘u.

The target object of $\ell _f$ (equivalently, the source of $r_f$) is often called the *image* of *f*, with respect to $(\mathcal {L},\mathcal {R})$. Note that the image is unique up to unique isomorphism. When we choose a specific factorization for each arrow, the object $\operatorname {Im}(f)$ is uniquely determined.

### Definition 2.2

A *functorial factorization system* on $\mathcal {C}$ is an assignment, to each morphism $f \in \text {Mor}(\mathcal {C})$, of a pair of morphisms $\ell _f, r_f$ such that $f = r_f \circ \ell _f$, that extends to a functor $Q:\mathcal {C}^{\rightarrow } \rightarrow \mathcal {C}$ (where $\mathcal {C}^{\rightarrow }$ is the arrow category of $\mathcal {C}$), mapping every *f* to its image *Q*(*f*).

**Figure:**
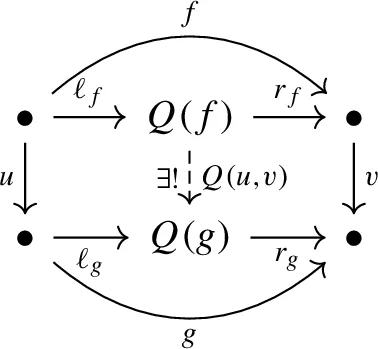

Note that every OFS with a choice of a factorization for each arrow is a functorial factorization system.

### Remark 2.3

In this paper, when we say that a category has an OFS, we will always mean that it is also equipped with a choice of factorizations $f=r_f\circ \ell _f$ for every arrow *f* in the category. (Note that this is analogous to choosing a cleavage for a fibration.) We will require that for each identity arrow $\operatorname {id}_{A}$ we choose $\ell _{\operatorname {id}_{A}}=\operatorname {id}_{A}=r_{\operatorname {id}_{A}}$.

### Remark 2.4

There is a different notion of functional factorization system in the literature that should not be confused with the one described above (which is in line with Riehl’s notes on factorization systems). This was used in [15] for instance.

In [12], Korostenski and Tholen proved a 2-monadicity result for orthogonal factorization systems. The 2-monad they considered is the squaring 2-monad $(-)^{\rightarrow }$ on , sending any category to its arrow category (also known as its category of commutative squares). More precisely, they proved the following, although phrased slightly differently.

### Theorem 2.5

([12, Theorem B]) Orthogonal factorization systems, with a choice of factorizations for the arrows as in Remark 2.3, are the normal pseudo-algebras for the squaring 2-monad .

### Remark 2.6

Explicitly, the normal pseudo-algebra structure map associated to an orthogonal factorization system $(\mathcal {L},\mathcal {R})$ on a category $\mathcal {C}$ is the functor $Q:\mathcal {C}^{\rightarrow }\rightarrow \mathcal {C}$ that sends every  to the middle object of its chosen factorization , and sends every commutative square as on the left below to the unique morphism *Q*(*u*, *v*) on the right below induced by the functoriality of the orthogonal factorization system:

**Figure:**
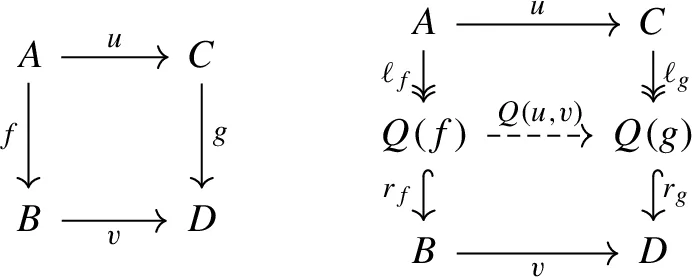

Interestingly, the functor *Q* contains all the data of the orthogonal factorization system $(\mathcal {L},\mathcal {R})$. The normality condition precisely imposes that *Q* is a (strict) retraction of the diagonal $\mathcal {C}\rightarrow \mathcal {C}^{\rightarrow }$, which sends *A* to $\operatorname {id}_{A}$ and f:A→B to (*f*, *f*). Considering the factorization

**Figure:**
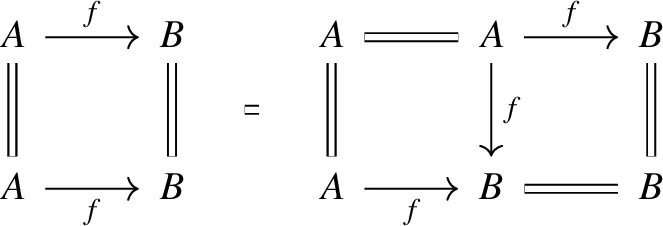

in $\mathcal {C}^{\rightarrow }$, one then finds that

$$\begin{aligned} Q(\operatorname {id}_{A},f)=\ell _f, \qquad Q(f,\operatorname {id}_{B})=r_f, \qquad f=Q(f,f)=r_f\circ \ell _f \end{aligned}$$

provided that the choice of factorization for the identity arrows is $\ell _{\operatorname {id}_{A}}=\operatorname {id}_{A}=r_{\operatorname {id}_{A}}$. We will always assume that in this paper. The morphisms in $\mathcal {L}$ are precisely those *f* for which $Q(f,\operatorname {id})$ is an isomorphism, and the morphisms in $\mathcal {R}$ are precisely those *f* for which $Q(\operatorname {id},f)$ is an isomorphism.

As was noted in [12], $\mathcal {C}^\rightarrow$ is the free category with an orthogonal factorization system on a given category $\mathcal {C}$. Its factorization system is given as follows: the left class consists of the morphisms of the form (*i*, *u*) with *i* iso and *u* any morphism in $\mathcal {C}$, and the right class consists of the morphisms of the form (*v*, *j*) with *v* any morphism in $\mathcal {C}$ and *j* iso. A general morphism (*u*, *v*) then factorizes as $(u,\operatorname {id})\circ (\operatorname {id},v)$. This is indeed the free algebra on $\mathcal {C}$ with respect to the squaring 2-monad $(-)^{\rightarrow }$. One can in fact check that the multiplication $\mu _{\mathcal {C}}:{(\mathcal {C}^\rightarrow )}^\rightarrow \rightarrow \mathcal {C}^\rightarrow$ of the monad precisely translates to the left and right classes described above.

### Remark 2.7

Orthogonal factorization systems are in particular *algebraic weak factorization systems*, which were introduced in [16] under the name “natural weak factorization systems". As a consequence, we obtain the further monadicity results shown in Proposition 2.8 below.

We can extract from $Q:\mathcal {C}^{\rightarrow }\rightarrow \mathcal {C}$ two other functors $L,R:\mathcal {C}^{\rightarrow }\rightarrow \mathcal {C}^{\rightarrow }$, sending

**Figure:**
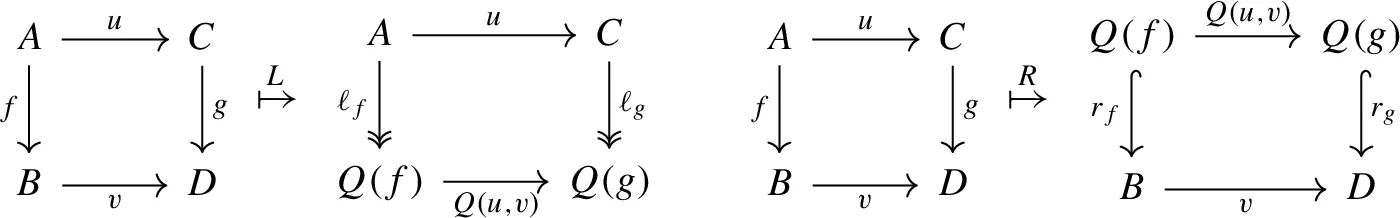

### Proposition 2.8

([16], Theorem 3.2) Let $(\mathcal {L},\mathcal {R})$ be an orthogonal factorization system on a category $\mathcal {C}$. Its associated functors $L,R:\mathcal {C}^{\rightarrow }\rightarrow \mathcal {C}^{\rightarrow }$ of Remark 2.7 extend respectively to an idempotent comonad and to an idempotent monad. The left class $\mathcal {L}$, viewed as a full subcategory of $\mathcal {C}^{\rightarrow }$, is precisely the category of coalgebras for *L*; as a consequence, it has all colimits, created by the projection to $\mathcal {C}^{\rightarrow }$. The right class $\mathcal {R}$, viewed as a full subcategory of $\mathcal {C}^{\rightarrow }$, is precisely the category of Eilenberg-Moore algebras for *R*; as a consequence, it has all limits, created by the projection to $\mathcal {C}^{\rightarrow }$.

We can now extract from Theorem 2.5 four flavours of morphisms between orthogonal factorization systems (see also Remark 2.3), corresponding to pseudo, lax, colax, and strict morphisms between normal pseudo-algebras. Each flavour of factorization yields a 2-category of categories equipped with an OFS, whose 1-arrows are given by morphisms of the corresponding type, and 2-cells are natural transformations.

### Definition 2.9

A *pseudo-morphism* of orthogonal factorization systems from $(\mathcal {C},(\mathcal {L}_{\mathcal {C}}, \mathcal {R}_{\mathcal {C}}))$ to $(\mathcal {D},(\mathcal {L}_{\mathcal {D}}, \mathcal {R}_{\mathcal {D}}))$ is a functor $F:\mathcal {C}\rightarrow \mathcal {D}$ that preserves both the left and the right classes; i.e., that sends morphisms in $\mathcal {L}_{\mathcal {C}}$ to morphisms in $\mathcal {L}_{\mathcal {D}}$ and morphisms in $\mathcal {R}_{\mathcal {C}}$ to morphisms in $\mathcal {R}_{\mathcal {D}}$.

A *lax morphism* of orthogonal factorization systems from $(\mathcal {C},(\mathcal {L}_{\mathcal {C}}, \mathcal {R}_{\mathcal {C}}))$ to $(\mathcal {D},(\mathcal {L}_{\mathcal {D}}, \mathcal {R}_{\mathcal {D}}))$ is a functor $F:\mathcal {C}\rightarrow \mathcal {D}$ that preserves the right class: $F(\mathcal {R}_{\mathcal {C}})\subseteq \mathcal {R}_{\mathcal {D}}$.

A *colax morphism* of orthogonal factorization systems from $(\mathcal {C},(\mathcal {L}_{\mathcal {C}}, \mathcal {R}_{\mathcal {C}}))$ to $(\mathcal {D},(\mathcal {L}_{\mathcal {D}}, \mathcal {R}_{\mathcal {D}}))$ is a functor $F:\mathcal {C}\rightarrow \mathcal {D}$ that preserves the left class: $F(\mathcal {L}_{\mathcal {C}})\subseteq \mathcal {L}_{\mathcal {D}}$.

A *strict morphism* of orthogonal factorization systems (with a choice of factorizations) from $(\mathcal {C},(\mathcal {L}_{\mathcal {C}}, \mathcal {R}_{\mathcal {C}}))$ to $(\mathcal {D},(\mathcal {L}_{\mathcal {D}}, \mathcal {R}_{\mathcal {D}}))$ is a functor $F:\mathcal {C}\rightarrow \mathcal {D}$ that preserves the chosen factorizations on the nose: $\ell _{F(f)}=F(\ell _f)$ and $r_{F(f)}=F(r_f)$ for all *f* in $\mathcal {C}_1$.

### Notation 2.10

We obtain the following 2-categories of categories equipped with an OFS, from the flavours of morphism above:

, whose 1-arrows are pseudo-morphisms of orthogonal factorization systems;
, whose 1-arrows are lax morphisms;
, whose 1-arrows are colax morphisms;
, whose 1-arrows are strict morphisms.

In all of them we use all natural transformations as 2-cells.

### Remark 2.11

Let $F:(\mathcal {C},(\mathcal {L}_{\mathcal {C}}, \mathcal {R}_{\mathcal {C}})) \rightarrow (\mathcal {D},(\mathcal {L}_{\mathcal {D}}, \mathcal {R}_{\mathcal {D}}))$ be a lax morphism of orthogonal factorization systems. Call $Q_{\mathcal {C}}$ and $Q_{\mathcal {D}}$ the normal pseudo-algebra maps corresponding to $(\mathcal {L}_{\mathcal {C}}, \mathcal {R}_{\mathcal {C}})$ and $(\mathcal {L}_{\mathcal {D}}, \mathcal {R}_{\mathcal {D}})$ and their chosen factorizations respectively. Then *F* preserves the factorizations of morphisms in $\mathcal {C}$ in a lax way. More precisely, given a morphism f:A→B in $\mathcal {C}$ and considering its factorization

**Figure:**
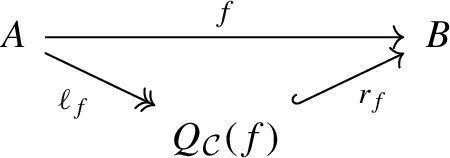

with $\ell _f\in \mathcal {L}$ and $r_f\in \mathcal {R}$, there exists a unique morphism $\rho _f:Q_{\mathcal {D}}(F(f))\rightarrow F(Q_{\mathcal {C}}(f))$ which makes the following diagram commute:

**Figure:**
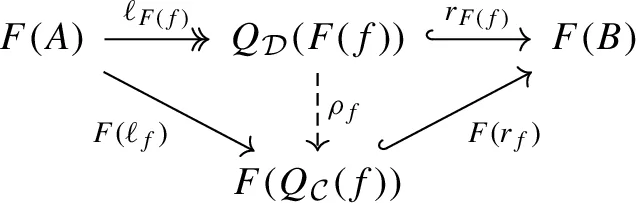

Indeed $F(r_f)\in \mathcal {R}_{\mathcal {D}}$, as *F* preserves the right class, and we obtain the unique lifting $\rho _f$ by definition of orthogonal factorization system. By uniqueness the morphisms $\rho _f$ form the components of a natural transformation $\rho :Q_{\mathcal {D}}F^{\rightarrow }\Rightarrow FQ_{\mathcal {C}}$.

Analogously, a colax morphism $F:(\mathcal {C},(\mathcal {L}_{\mathcal {C}}, \mathcal {R}_{\mathcal {C}})) \rightarrow (\mathcal {D},(\mathcal {L}_{\mathcal {D}}, \mathcal {R}_{\mathcal {D}}))$ of orthogonal factorization systems gives rise to a natural transformation $\lambda :FQ_{\mathcal {C}}\Rightarrow Q_{\mathcal {D}}F^{\rightarrow }$.

If *F* is a pseudo-morphism of orthogonal factorization systems, then for every f:A→B in $\mathcal {C}$ the morphism $\rho _f$ is an isomorphism. So in this case *F* preserves the factorizations up to isomorphism.

Notice then that a strict morphism *F* of orthogonal factorization systems preserves in particular both the left and the right classes. Indeed, a morphism is in the left class if and only if the right part of its factorization is an isomorphism, and *F* preserves the factorizations.

### Proposition 2.12

Let $(\mathcal {C},(\mathcal {L}_{\mathcal {C}}, \mathcal {R}_{\mathcal {C}}))$ and $(\mathcal {D},(\mathcal {L}_{\mathcal {D}}, \mathcal {R}_{\mathcal {D}}))$ be orthogonal factorization systems and corresponding normal pseudo-algebra maps $Q_\mathcal {C}$ and $Q_\mathcal {D}$. For a functor $F:\mathcal {C}\rightarrow \mathcal {D}$, the following are equivalent:

(i) *F* is a lax morphism (respectively, pseudo-morphism, strict morphism) of orthogonal factorization systems;
(ii) *F* extends to a lax morphism (respectively, pseudo-morphism, strict morphism) between normal pseudo-algebras for the squaring 2-monad $(-)^{\rightarrow }$ on ; i.e., there exists a natural transformation (respectively, a natural isomorphism, an identity) 
1 such that for every $A\in \mathcal {C}$ we have $\rho _{\operatorname {id}_{A}}=\operatorname {id}_{F(A)}$ and, for every commutative square in $\mathcal {C}$ as on the left below, the pentagon on the right below commutes:

**Figure:**
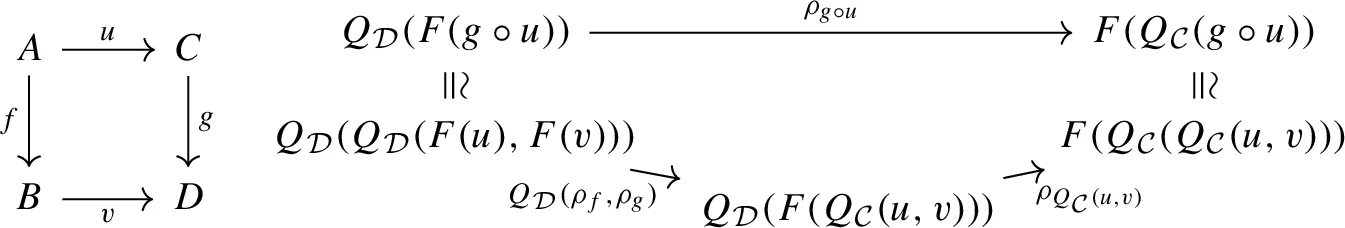

where the isomorphisms are given by the normal pseudo-algebra structures;
(iii) (based on [17, Sect. I.6.2]) there exists a natural transformation (respectively, a natural isomorphism, an identity) ρ as in (1) such that $(1,\rho ):L_\mathcal {D}\circ F\rightarrow F\circ L_\mathcal {C}$ is a lax morphism of comonads and $(\rho ,1):R_\mathcal {D}\circ F\rightarrow F\circ R_\mathcal {C}$ is a lax morphism of monads, where $L_\mathcal {C},R_\mathcal {C},L_\mathcal {D},R_\mathcal {D}$ are the functors of Proposition 2.8, for $\mathcal {C}$ and $\mathcal {D}$ respectively; i.e., for every f:A→B in $\mathcal {C}$ the following three diagrams commute:

**Figure:**
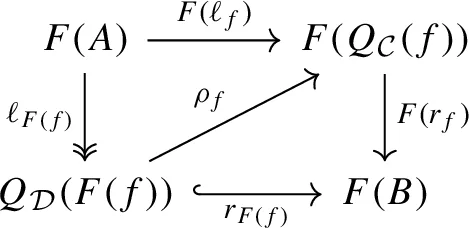

**Figure:**
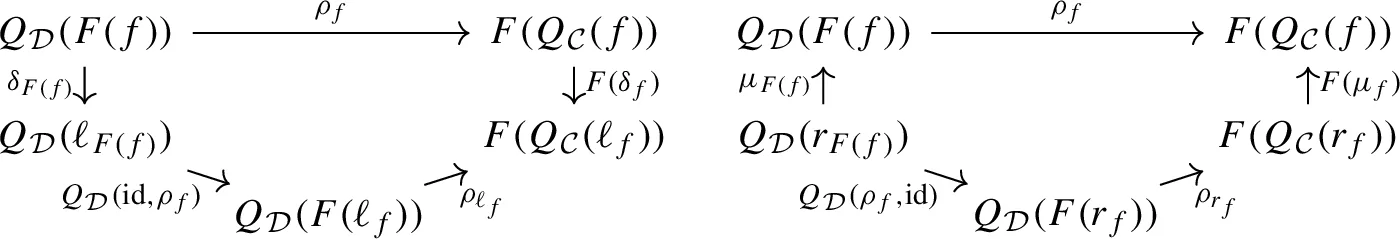

where $\delta _h$ and $\mu _h$ are given by

**Figure:**
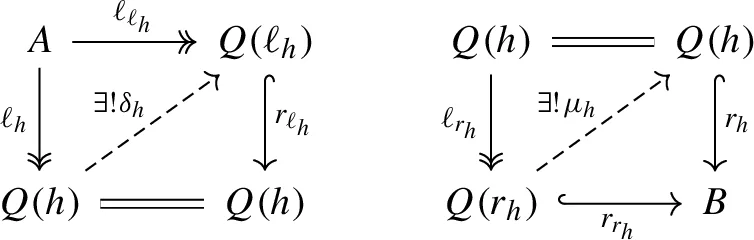

Moreover, the 2-cells between (lax) morphisms of normal pseudo-algebras for $(-)^{\rightarrow }$ are precisely the natural transformations between the underlying functors.

### Proof

We prove . Given f:A→B in $\mathcal {C}$, we can produce the component $\rho _f$ of ρ on *f* as in Remark 2.11. Then ρ is natural by the uniqueness of the liftings required by definition of orthogonal factorization system. And clearly $\rho _{\operatorname {id}}=\operatorname {id}$, because the choice of factorization for the identity is always by identities. It is straightforward to show that the pentagon commutes, using again the uniqueness of the liftings.

We now prove . We can take the needed ρ to be the natural transformation ρ given by (*ii*). By Remark 2.6, naturality of ρ and $\rho _{\operatorname {id}}=\operatorname {id}$, we obtain that the two triangles in the first diagram in (*iii*) commute. Indeed, considering the morphism in $\mathcal {C}^{\rightarrow }$ on the left below, naturality of ρ gives the square on the right below

**Figure:**
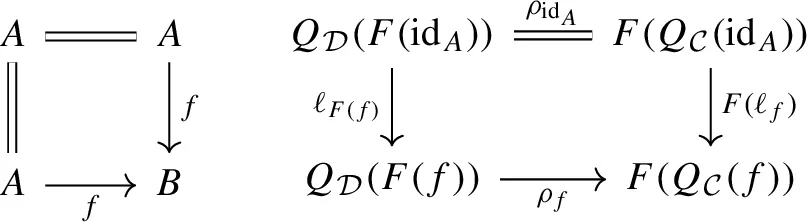

and considering the morphism $(f,\operatorname {id})$ we obtain the other triangle. Evaluating the pentagon of (*ii*) for the morphism $(\operatorname {id},f)$ (respectively, $(f,\operatorname {id})$) in $\mathcal {C}^{\rightarrow }$, we obtain the pentagon on the left (respectively, on the right) of (*iii*). It is indeed straightforward to show that the isomorphisms of the pentagon of (*ii*) become the appropriate morphisms δ and μ.

Finally, we prove . The lax morphism of monads (ρ,1) induces a functor . So by Proposition 2.8, *F* preserves the right class. If ρ is a natural isomorphism, the two triangles in the first diagram in (*iii*) show that *F* preserves both the left and the right classes. If ρ is the identity, the same triangles show that *F* preserves the factorizations.

It is then straightforward to show that the 2-cells between (lax) morphisms of normal pseudo-algebras for $(-)^{\rightarrow }$ are just the natural transformations, by uniqueness of liftings. □

### Corollary 2.13

 and  are precisely the 2-categories  (respectively, ) of normal pseudo-algebras for $(-)^{\rightarrow }$ on  and pseudo-morphisms (respectively, lax morphisms) between them.

### Proof

This follows immediately from Proposition 2.12. □

### Remark 2.14

The results characterizing the pseudo, lax and colax morphisms of normal pseudo-algebras for the squaring monad could also be obtained from a more general result about factoring cones proved by Garner in Sect. 5 of [13].

The following result will be needed to define double orthogonal factorization systems.

### Proposition 2.15

The 2-categories , , , and  have all pullbacks of strict morphisms.

### Proof

Let  and  be categories equipped with orthogonal factorization systems, and strict morphisms  and . We show that the pullback category  admits an orthogonal factorization system, and that the associated projections and the unique arrows determined by its universal property for arrows in  are factorization-preserving. Additionally, when working in ,  or  respectively, we show that the induced unique maps into the pullback are respectively pseudo, lax or colax.

Denote the factorization systems on  and  by  and  respectively. We form a factorization system on  whose classes are given by:
For a morphism $\left(f,g\right):\left(A,B\right)\to \left(A^{\prime },B^{\prime }\right)$ in  we choose the factorization:

**Figure:**
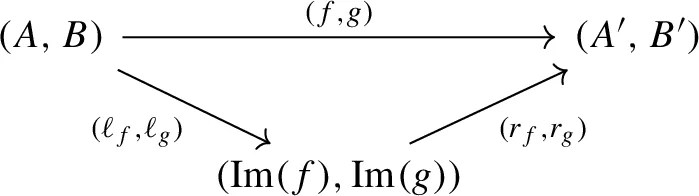

where $f=r_f\circ \ell _f$ and $g=r_g\circ \ell _g$ are the factorizations of *f*, *g* in , and  respectively. Note that this is well-defined because *F* and *G* are strict morphisms between categories with an OFS. Because  and  are closed under composition, so is . Likewise, by the fact that  and  contain all isomorphisms,  does as well. An identical argument holds for ; therefore,  is a factorization system. We now show orthogonality. Given a diagram in  of the form:

**Figure:**
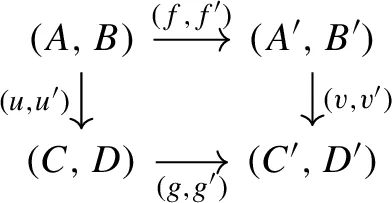

with  and , consider the following square in :

**Figure:**
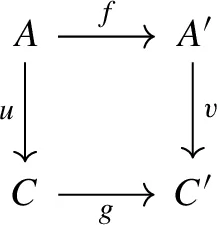

We have , so by orthogonality there exists a unique morphism $e:C\to A^{\prime }$ making the following commute:

**Figure:**
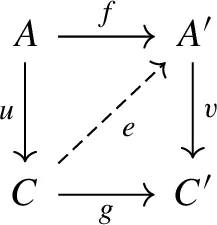

Applying an identical argument to the square likewise constructed in  yields $e^{\prime }:D\to B^{\prime }$ satisfying a similar condition. Furthermore, *F*(*e*) and $G(e^{\prime })$ are both the unique diagonal arrow in the following diagram in $$\mathcal {C}$$,

**Figure:**
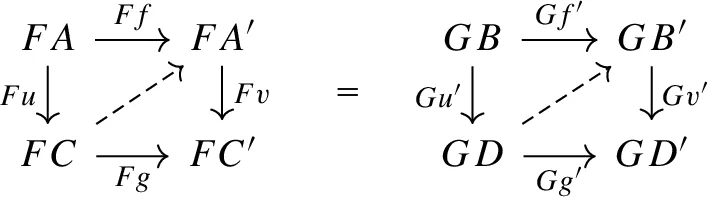

Hence, $F\left(e\right)=G\left(e^{\prime }\right)$ and $\left(e,e^{\prime }\right)$ is a well-defined arrow in the pullback category. Now, $\left(e,e^{\prime }\right)$ is the unique arrow making the following diagram commute:

**Figure:**
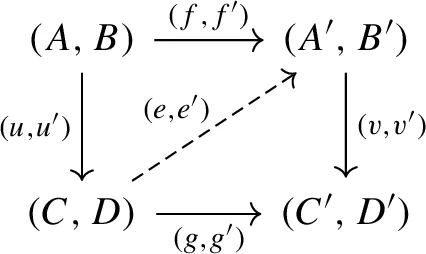

This yields orthogonality of the factorization system  on .

Consider the projection map . For , we have , hence $P_1$ preserves the left class, and likewise for the right class. Furthermore, $P_1$ preserves the chosen factorization by construction. Therefore, $P_1$ is a strict morphism of categories equipped with a factorization system, as in Definition 2.9. An analogous argument applies to $P_2$. With this established, we now show that the induced maps into the pullback satisfy the relevant properties for , , , and .

Suppose that , and  are lax morphisms such that $$F\circ F'=G\circ G'$$. By the universal property of pullbacks, there exists a unique  such that $$F' = P_1\circ I$$ and $$G' = P_2\circ I$$. Explicitly, *I* maps an object  to $\left(F^{\prime }\left(D\right),G^{\prime }\left(D\right)\right)$, and a morphism $h:D\to D^{\prime }$ to $\left(F^{\prime }\left(h\right),G^{\prime }\left(h\right)\right)$.

If $F^{\prime }$ and $G^{\prime }$ both preserve right classes, and , we have , hence . Thus, *I* is a lax morphism of OFSs. If $F^{\prime }$ and $G^{\prime }$ both preserve the left classes, an identical argument shows that *I* also preserves the left class, so *I* is a colax morphism of OFSs. It also follows that if both $F^{\prime }$ and $G^{\prime }$ are pseudo-morphisms of OFSs (i.e., preserve both the left and right classes), so is *I*. Hence, the morphisms into the pullback will be pseudo, lax, or colax as required.

When $F^{\prime }$ and $G^{\prime }$ are strict, let *u* be any morphism in , with chosen factorization $$u=r_u\circ \ell _u$$. Since $$P_1$$ and $$P_2$$ are strict pseudo-morphisms of categories with an OFS, *I*(*u*) in  factors as $$(F'(u), G'(u)) = (F'(r_u), G'(r_u)) \circ (F'(\ell _u), G'(\ell _u))$$; therefore, *I* preserves factorizations strictly.

Because  admits 2-pullbacks, and 2-cells in , , , and  are ordinary natural transformations, the relevant 2-dimensional universal properties are satisfied.

We have shown that  admits an orthogonal factorization system. Furthermore, all morphisms induced by the pullback are appropriately strict, pseudo, lax, or colax. Therefore, pullbacks of strict morphisms exist in , , , and . □

## Double Orthogonal Factorization Systems

In this section, we propose our notion of double orthogonal factorization system (DOFS). We first introduce our notation and terminology for double categories and then present some motivating examples that led us to this notion of DOFS. We observe that some examples are more lax than others, and then focus primarily on two cases of double orthogonal factorization systems: the lax and the pseudo case. As an extension to what has been discussed in the previous section, one may easily generalize this to the strict and colax versions of our theory, and we will see a couple of examples of these later in this paper.

We define double orthogonal factorization systems with an approach of internal category theory. We then explicitly describe them in terms of compatible orthogonal factorization systems on the arrows and the double cells of a double category. In Sect. 4, we will prove monadicity results for double orthogonal factorization systems, further justifying our notions.

### Double Categories

We review the internal definition of double categories, and establish our notation. To begin, recall the definition of a pseudo-category internal to a 2-category, following [18].

#### Definition 3.1

Let  be a 2-category where all pullbacks depicted below exist. A *pseudo-category* $\mathbb {C}$ *internal to*  consists of objects $C_0$ and $C_1$ of  (called the object of objects and the object of arrows, respectively) together with morphisms $\operatorname {src}$, $\operatorname {tgt}$, *i* and ⊗ (source, target, identity and composition, respectively) as below

**Figure:**
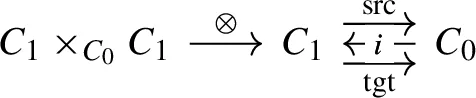

and invertible 2-cells $\alpha , \lambda$, and ρ:

**Figure:**
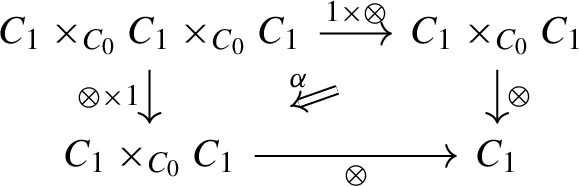

**Figure:**
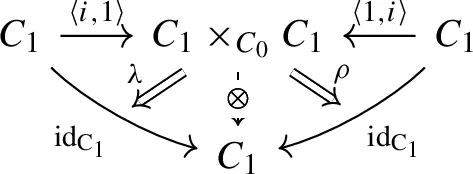

The above cells are further required to satisfy the usual coherence conditions, along with the usual equalities for arrow composition (with several weakened by isomorphisms satisfying further coherence conditions). These requirements are fully listed in [18]. When the 2-cells $\alpha , \lambda$, and ρ are identities, we say that the pseudo-category internal to  is *strict*, or a *category internal to* . When the 2-cells λ and ρ are identities, the pseudo-category is called *normal*.

#### Definition 3.2

A *pseudo-double category* is a pseudo-category internal to , the 2-category of categories, functors, and natural transformations.

We refer to the objects of  simply as *objects*; arrows of  are referred to as *arrows* and drawn horizontally. The objects of  will be referred to as *proarrows*, and drawn vertically. Note that we use the term ‘arrows’ for what some call the *tight arrows* of a double category, and the term ‘proarrows’ for what they call the *loose arrows* of a double category.

In this paper we will work with a 2-category of pseudo-categories internal to a 2-category . The morphisms will be the internal lax functors as described in the next definition.

#### Definition 3.3

Suppose that $\mathbb {C}$ and $\mathbb {D}$ are pseudo-categories internal to a 2-category . An internal lax functor $F: \mathbb {C} \rightarrow \mathbb {D}$ consists of the following:

- Morphisms $F_0:C_0 \rightarrow D_0$ and $F_1:C_1 \rightarrow D_1$ in ;
- 2-Cells $\varphi , \theta$ in  as follows:

**Figure:**
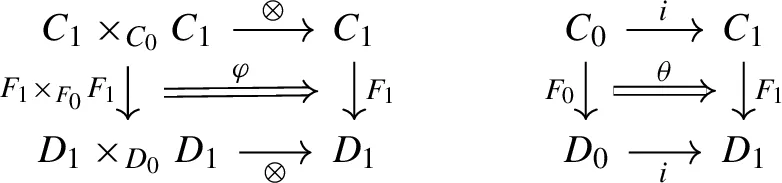

satisfying several coherence conditions (see [18]).

When φ and θ are invertible, *F* is said to be an internal *pseudo*-functor; in the situation where they are reversed, *F* is said to be *oplax*. When θ is an identity, *F* is said to be *unitary* or *normal*.

Applying the previous definition to pseudo-categories internal to  yields a notion of functors between double categories.

#### Definition 3.4

A (normal, lax, oplax, pseudo) *double functor* is a (normal, lax, oplax, pseudo) functor internal to .

### Motivating Examples

#### Example 3.5

*(The double category of commutative squares in* $\mathcal {C}$, $\mathbb {S}\textrm{q} (\mathcal {C})$*)*

For any category $\mathcal {C}$, the double category $\mathbb {S}\textrm{q} (\mathcal {C})$, introduced by Ehresmann in his original work on double categories [19], has the same objects as $\mathcal {C}$, both the arrows and the proarrows of $\mathbb {S}\textrm{q} (\mathcal {C})$ are the arrows of $\mathcal {C}$, and the double cells are the commutative squares in $\mathcal {C}$. When $\mathcal {C}$ is a category with an OFS $(\mathcal {L},\mathcal {R})$, we can lift the factorization structure on the arrows of $\mathbb {S}\textrm{q} (\mathcal {C})$, here drawn horizontally, to a factorization structure on the double cells of $\mathbb {S}\textrm{q} (\mathcal {C})$ as follows.

Given any cell α of $\mathbb {S}\textrm{q} (\mathcal {C})$ (which is completely determined by its boundary arrows), we factor the (horizontal) arrows in its boundary as $f=r_f\circ \ell _f$ and $g=r_g\circ \ell _g$ according to the factorization system on $\mathcal {C}$. By the functoriality of the factorization system there is a unique arrow *q* as in the following diagram.
2This shows that α factors into a horizontal (arrow-direction) composition of a cell $l_{\alpha }$ with (horizontal) arrows in $\mathcal {L}$, followed by a cell $r_{\alpha }$ with (horizontal) arrows from $\mathcal {R}$. Hence, $\mathbb {S}\textrm{q} (\mathcal {C})$ has a factorization system $(\mathcal {L}', \mathcal {R}')$ on its double cells where the left class $\mathcal {L}'$ consists of cells with morphisms from $\mathcal {L}$ as arrows and the right class $\mathcal {R}'$ consists of cells with morphisms from $\mathcal {R}$ as arrows. Both classes of cells are closed under horizontal composition. It is not hard to show that the factorization system on the double cells is orthogonal as factorization system on the category with vertical arrows as objects and double cells as arrows (using horizontal composition).

Thus, we have an OFS $(\mathcal {L},\mathcal {R})$ on the category $\mathbb {S}\textrm{q} (\mathcal {C})_0$ of objects and arrows (or, horizontal arrows) and an OFS $(\mathcal {L}', \mathcal {R}')$ on the category $\mathbb {S}\textrm{q} (\mathcal {C})_1$ or proarrows (or, vertical arrows) and double cells (with horizontal composition). We will now investigate how these factorization systems interact with the structure of the double category. First note that the source, target and unit morphisms preserve the factorizations, so they are strict morphisms of categories with an OFS.

The vertical composition of two cells factors as follows:

**Figure:**
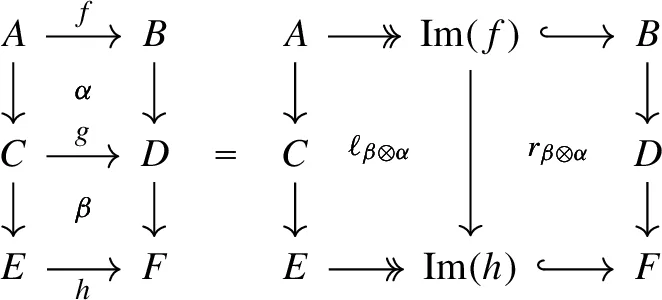

where the center arrow exists by functoriality of the OFS on . On the other hand, the composition of the factorizations of both cells is given by:

**Figure:**
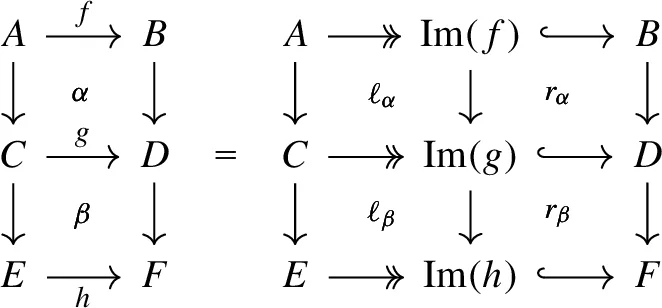

The equalities $\ell _{\beta \otimes \alpha } = \ell _{\beta } \otimes \ell _{\alpha }$ and $r_{\beta \otimes \alpha } = r_{\beta } \otimes r_{\alpha }$ hold by uniqueness of the middle arrow, which is a consequence of functoriality of the OFS. It follows that the composition of the codomains of $\ell _{\beta }$ and $\ell _{\alpha }$ is equal to the codomain of the composition of $\ell _{\beta \otimes \alpha }$.

Hence, the double category $\mathbb {S}\textrm{q} (\mathcal {C})$ has a factorization system $(\mathcal {L}', \mathcal {R}')$ on its double cells, such that $\mathcal {L}'$ and $\mathcal {R}'$ are closed under vertical composition and furthermore, with the chosen factorization for cells indicated in diagram (2), all structure arrows are strict morphisms between OFSs.

#### Remark 3.6

In our first example, all structure maps preserve the factorization systems strictly. The next example presents a factorization system on $\mathbb {S}\textrm{pan}(\mathcal {C})$ where vertical composition only preserves the right class. However, source, target and unit arrows are strict.

#### Example 3.7

*(The span double category on* $\mathcal {C}$, $\mathbb {S}\textrm{pan}(\mathcal {C})$*)*

Let $\mathcal {C}$ be a category with all pullbacks and an OFS $(\mathcal {L}, \mathcal {R})$ with chosen factorizations,

**Figure:**
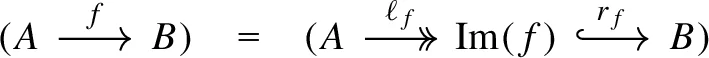

The double category $\mathbb {S}\textrm{pan}(\mathcal {C})$ has morphisms in $\mathcal {C}$ as arrows and spans of $\mathcal {C}$ as proarrows (proarrow composition is through chosen pullbacks); further, a double cell is defined by an arrow from the apex of its domain span to the apex of its codomain making both squares commute as in the following diagram.
3Since $\mathcal {C}$ has an OFS, any cell in $\mathbb {S}\textrm{pan}(\mathcal {C})$ can be factorized in the following manner.

**Figure:**
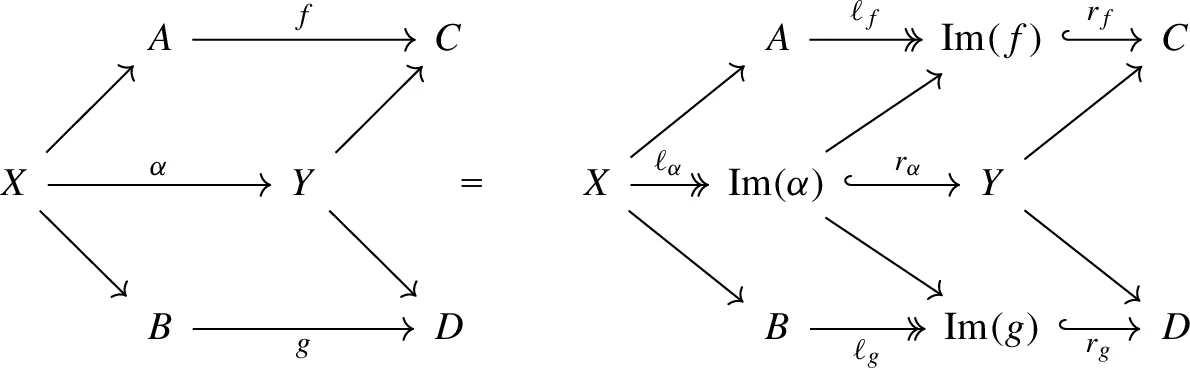

giving rise to the factorization system on $\mathbb {S}\textrm{pan}(\mathcal {C})_1$ where a cell (3) is in the left class $\mathcal {L}'$ when all three horizontal arrows *f*, *g*, and α are in $\mathcal {L}$, and it is in the right class $\mathcal {R}'$ if all three of these arrows are in $\mathcal {R}$. It is clear that both classes of this system are closed under horizontal composition and contain all horizontally invertible cells. It is also clear that the source, target and units functors preserve the chosen factorizations.

We now show that the right class $\mathcal {R}'$ is closed under vertical composition. Let θ and ψ be vertically composable double cells in the right class. Composing them yields the following.

**Figure:**
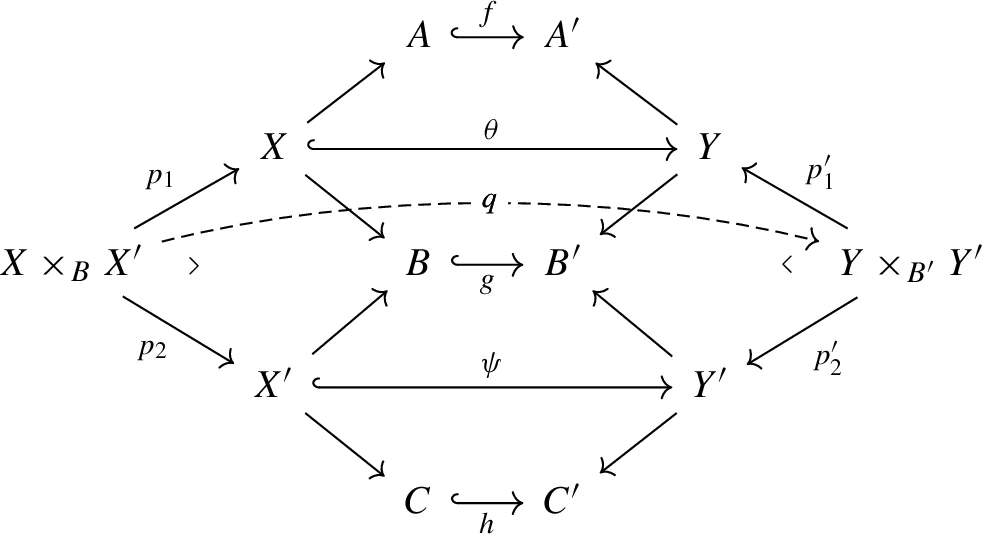

We need to show that the center arrow $q:X\times _BX'\rightarrow Y\times _{B'}Y'$ induced by the universal property of the right pullback is also in $\mathcal {R}$. To this end, we will show that it satisfies the right lifting property with respect to any morphism in $\mathcal {L}$. Suppose we have any morphism e:R↠S in $\mathcal {L}$ which is part of a commutative square on the left, as in the following diagram:

**Figure:**
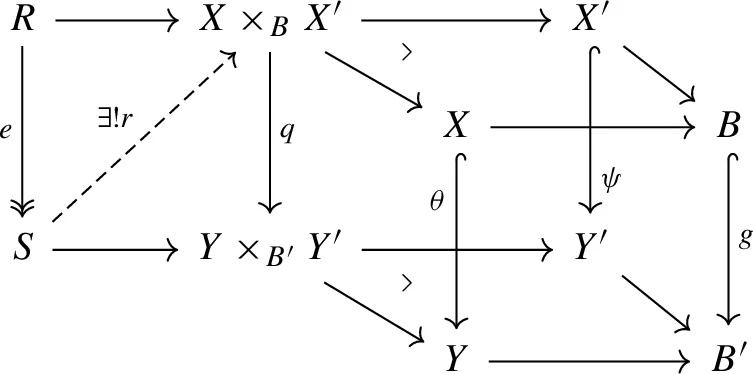

We wish to show that there exists a unique $r:S \rightarrow X \times _B X'$ giving us two commutative triangles to make up the square. By lifting *e* against θ,g, and ψ (with squares formed by composing upper segments of the diagram appropriately), we obtain unique morphisms $f_1, f_2$, and $f_3$ as in the following diagram,

**Figure:**
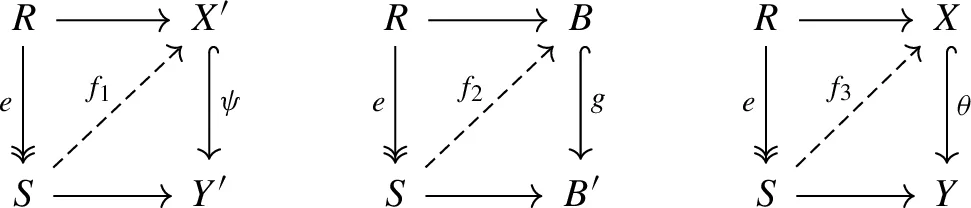

Uniqueness of each lifting, along with the pullback’s universal property, guarantees a unique *r* filling the left square as desired.

**Figure:**
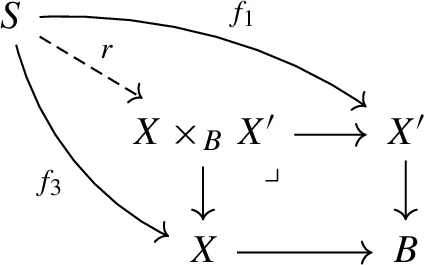

Thus, *q* is right-orthogonal to any map in the left class $\mathcal {L}$, so *q* is in the right class $\mathcal {R}$.

We show in Example 3.9 that vertical composition does not necessarily preserve the left class of cells, $\mathcal {L}'$, in this case. We show this for the case that .

#### Example 3.8

*(The double category of relations on any regular category* $\mathcal {C}$, $\mathbb {R}\textrm{el}(\mathcal {C})$ *)* Let  be a regular category. Then the canonical factorization system on $\mathcal {C}$ has regular epimorphisms as its left class $\mathcal {L}$ and monomorphisms as its right class $\mathcal {R}$. We assume again that a factorization $f=r_f\circ \ell _f$ has been chosen for each arrow *f* in $\mathcal {C}$. The double category of relations on $\mathcal {C}$ has morphisms of $\mathcal {C}$ as arrows and relations in $\mathcal {C}$ as proarrows. This means that a proarrow *R* from *X* to *Y* is given by a monic arrow $R\hookrightarrow X\times Y$ in $\mathcal {C}$. A double cell

**Figure:**
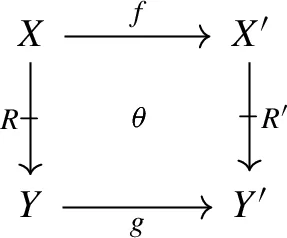

in $\mathbb {R}\textrm{el}(\mathcal {C})$ is given by an arrow $\theta :R\rightarrow R'$ in $\mathcal {C}$ such that the following diagram commutes in $\mathcal {C}$:

**Figure:**
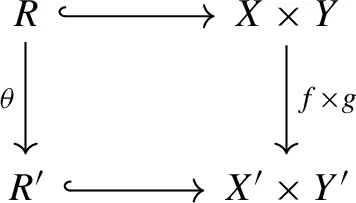

We introduce a factorization system on the category of proarrows and cells in $\mathbb {R}\textrm{el}(\mathcal {C})$ as follows:

- the left class $\mathcal {L}'$ consists of cells with regular epimorphisms as arrow domain and codomain and a regular epimorphism for the arrow θ between the relations.

**Figure:**
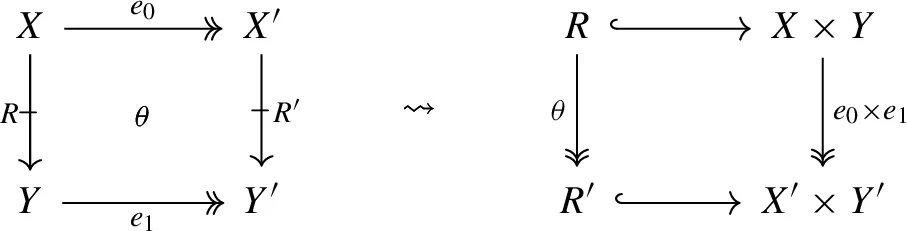
- the right class $\mathcal {R}'$ consists of cells with monomorphisms as arrow domain and codomain and a monomorphism for the arrow θ between the relations.

**Figure:**
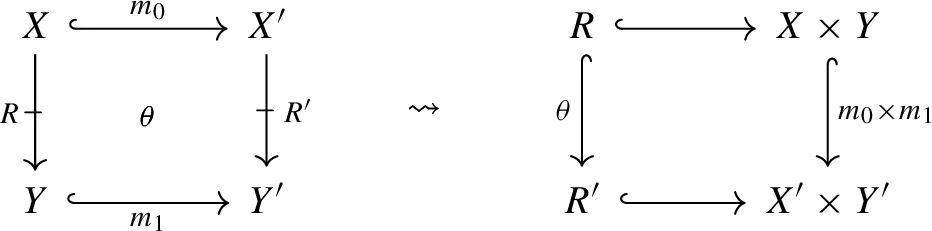

We now verify that the right class $\mathcal {R}'$ of this factorization system is closed under vertical composition. To this end, we first recall the definition of vertical composition. Given cells θ and α in $\mathbb {R}\textrm{el}(\mathcal {C})$, their vertical composition corresponds to an arrow $\theta \otimes \alpha$ in $\mathcal {C}$:

**Figure:**
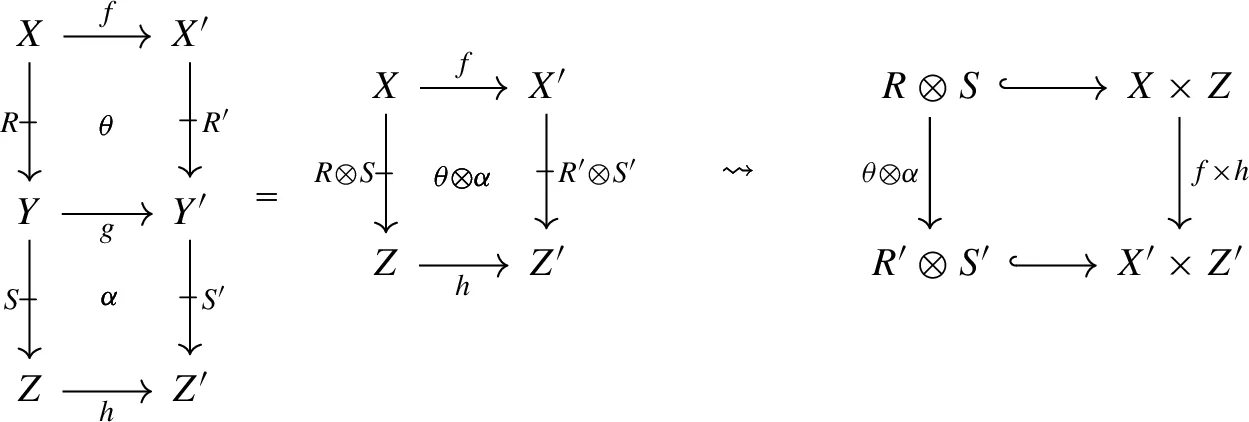

A morphism $t:R \times _Y S \rightarrow X \times Z$ is obtained by the universal property of X×Z via the diagram:

**Figure:**
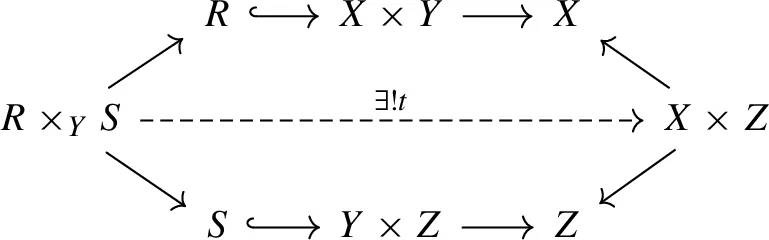

The image of *t* (from the canonical OFS on ) gives a subobject R⊗S of X×Z:

**Figure:**
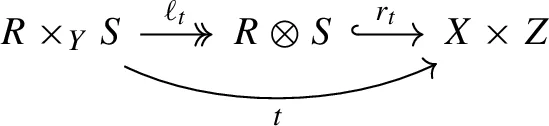

Furthermore, by the universal property of the pullback ${R'\times _{Y'} S'}$, we have a morphism $\theta \times \alpha :R \times _Y S \rightarrow R' \times _{Y'}S'$:

**Figure:**
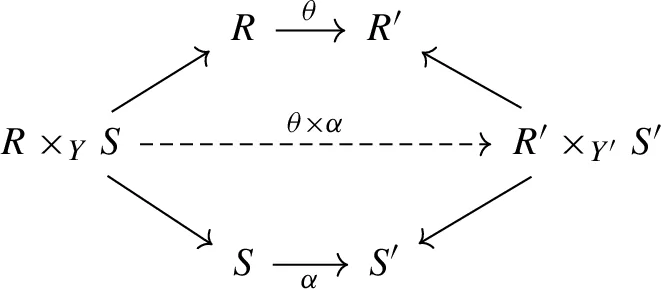

Finally, by the commutativity of the outer rectangle in the following diagram, together with the functoriality of the canonical OFS on *C*, there exists a unique morphism $$\theta \otimes \alpha : R\otimes S\rightarrow R'\otimes S'$$ between the images of *t* and $t^{\prime }$:

**Figure:**
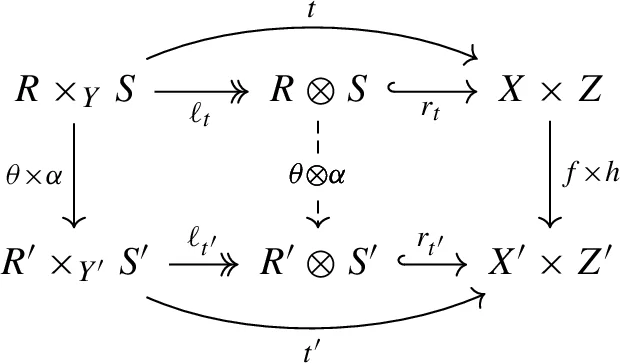

Observe that if $f:X\to X^{\prime }$ and $h:Z\to Z^{\prime }$ are monomorphisms, then their product $$f\times h:X\times Z\rightarrow X'\times Z'$$ is also a monomorphism. Under this assumption, and since the right-hand square in the above diagram commutes, it follows that $$r_{t'}\circ (\theta \otimes \alpha )$$ is a monomorphism. Consequently, $$\theta \otimes \alpha $$ is itself a monomorphism. This completes the proof that the right class $$\mathcal {R}'$$ is closed under vertical composition.

The left class $\mathcal {L}'$, however, is not necessarily closed under vertical composition, as we shall demonstrate in Example 3.9.

We now examine factorizations of cells in $\mathbb {R}\textrm{el}(\mathcal {C})$. Let

**Figure:**
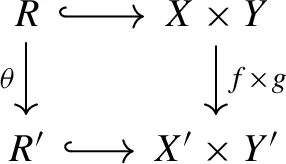

be a double cell. We factor θ as $r_\theta \circ \ell _\theta$ and f×g as $(r_f\times r_g)\circ (\ell _f \times \ell _g)$. By the functoriality of the canonical OFS on *C*, there exists a unique morphism as indicated in the following diagram:

**Figure:**
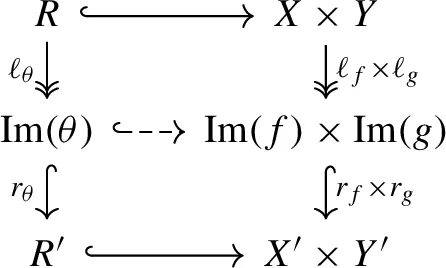

Hence, we can write any double cell in $\mathbb {R}\textrm{el}(\mathcal {C})$ as a cell in the left class $\mathcal {L}'$, followed by a cell in the right class $\mathcal {R}'$.

Next, we examine orthogonality. Consider an equation

**Figure:**
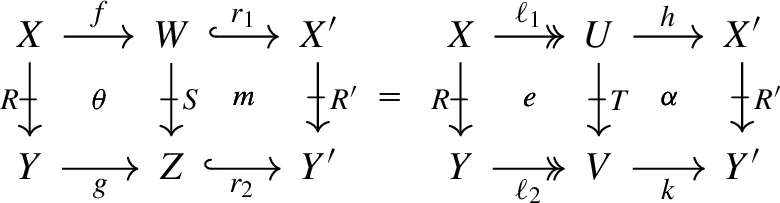

where $e\in \mathcal {L}'$ and $m\in \mathcal {R}'$. Equivalently, the following two commutative diagrams are equal:

**Figure:**
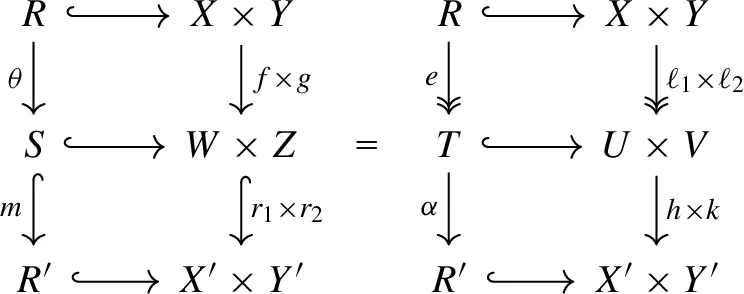

By the lifting property, applied respectively to the three squares below, there exist unique morphisms ψ, *a*, and *b*:

**Figure:**
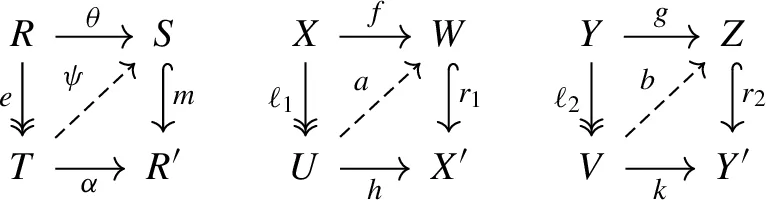

These morphisms induce the inner square in the diagram

**Figure:**
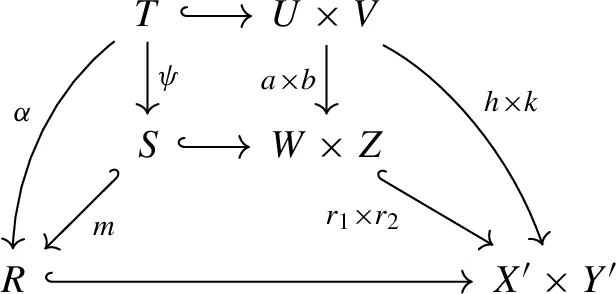

Since $r_1\times r_2$ is a monomorphism, and all other regions and the outer square of the diagram commute, it follows that the inner square also commutes. Hence, this square defines a double cell, which is precisely the required lift for the original equation.

#### Example 3.9

Consider the following diagram of double cells in .

**Figure:**
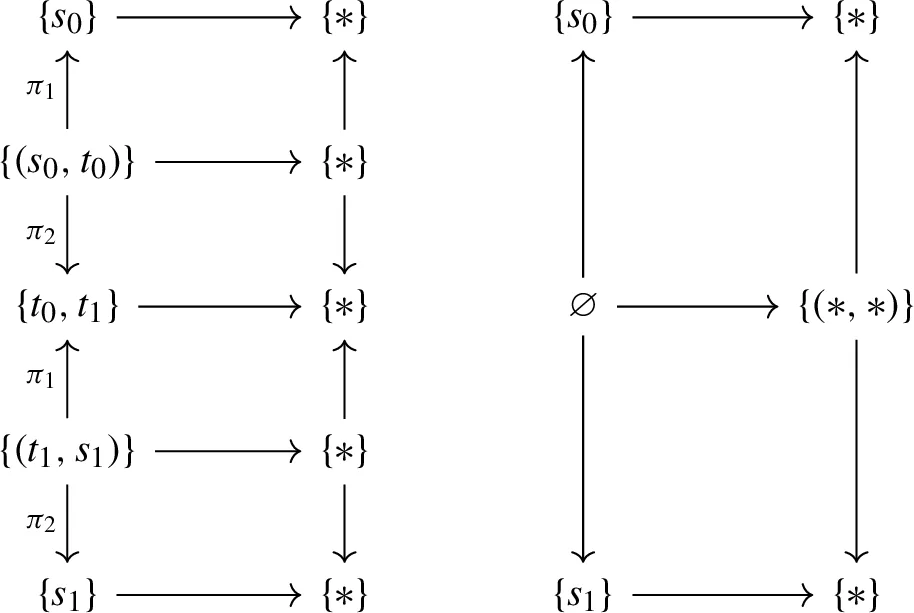

On the left side we have two vertically composable cells that are both in the left class $\mathcal {L}'$. However, their vertical composition, given on the right is clearly not in $\mathcal {L}'$: the arrow $\varnothing \rightarrow \{(*,*)\}$ is clearly not surjective. Note that this example applies to  as well.

#### Example 3.10

*(The cospan double category on* $\mathcal {C}$, $\mathbb {C}\textrm{ospan}(\mathcal {C})$*)* Let $\mathcal {C}$ be a category with pushouts and an orthogonal factorization system $(\mathcal {L}, \mathcal {R})$. Consider the double category $\mathbb {C}\textrm{ospan}(\mathcal {C})$, whose cells are given by commutative diagrams of the form:

**Figure:**
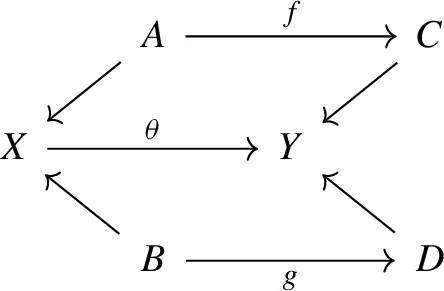

Using the underlying OFS on $\mathcal {C}$, we can factorize any cell in the following manner, with $\bar{f}, \bar{g}$ existing by functoriality of the OFS on $\mathcal {C}$:

**Figure:**
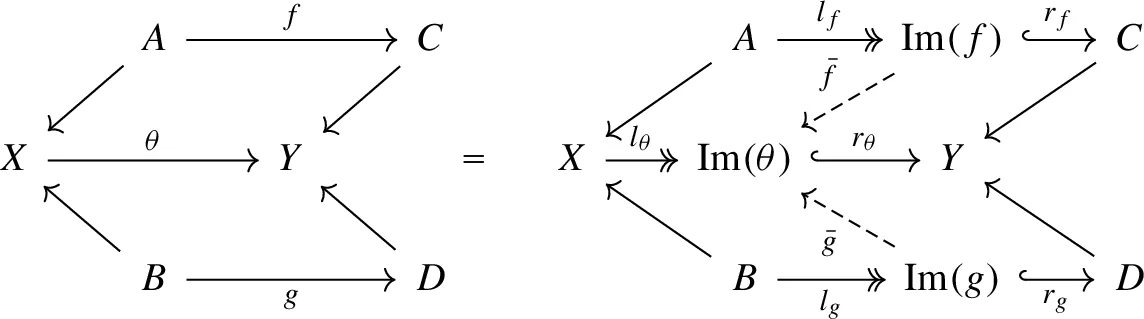

Note that this is the horizontal opposite of $\mathbb {S}\textrm{pan}(\mathcal {C}^{{\operatorname {op}}})$, so by a dual argument to the case of spans, it can be shown that the left class of double cells (whose arrows are in $\mathcal {R}$) is closed under vertical composition. However, the right class need not be closed under vertical composition; this can be seen by considering the dual of 3.9 in .

#### Example 3.11

*(The double category of paths in* $\mathcal {C}$, $\mathbb {P}\textrm{ath}\,\mathcal {C}$.*)* For a category $\mathcal {C}$, we define the double category $\mathbb {P}\textrm{ath}\,\mathcal {C}$, following the construction and notation in [20], such that the objects are the objects of $\mathcal {C}$, horizontal arrows are morphisms of $\mathcal {C}$, vertical arrows are paths in $\mathcal {C}$, i.e., composable sequences of arrows,

**Figure:**
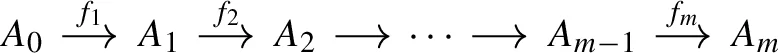

We denote such a path by $\langle f_i\rangle _{1\le i \le m}$. To define the double cells in this double category, we will use the following notation for arrows in a path: For any $i,j \in \{1,\cdots , m\}$, such that i≤j, we denote the composition of the arrows in the path between $A_i$ and $A_j$ by

4 $$\begin{aligned} f_j^i = {\left\{ \begin{array}{ll} f_jf_{j-1}\cdots f_{i+1} &  \text {if } i< j\\ \mathrm {id_{A_i}}&  \text {if } i=j \end{array}\right. } \end{aligned}$$

We will also write [m+1] for the set $\{0,1,\ldots ,m\}$.

A double cell with boundary

**Figure:**
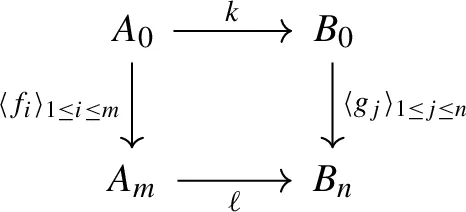

is then given by a pair $(\varphi , \langle h_i \rangle _{0\le i \le m})$ where

$$\begin{aligned} \varphi :[m+1] \rightarrow [n+1] \end{aligned}$$

is an order preserving function with φ(0)=0 and φ(m)=n, and the $h_i :A_i\rightarrow B_{\varphi (i)}$ are morphisms of $\mathcal {C}$ such that $h_0=k$, $h_m=\ell$ and for every i∈[m+1], the diagram

**Figure:**
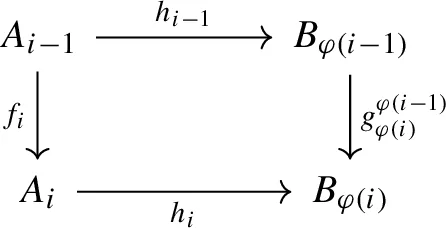

commutes. These double cells are called *fences* in [20]. An example of a double cell of $\mathbb {P}\textrm{ath}\,\mathcal {C}$ is given by,
5Vertical composition of these cells is defined by concatenation. Horizontal composition is defined by following the threads of the cross arrows and composing:

$$\begin{aligned} (\psi ,\langle k_j\rangle _{1\le j\le n})(\varphi ,\langle h_i\rangle _{1\le i\le m})=(\psi \varphi ,\langle k_{\varphi (i)}h_i\rangle _{1\le i\le m}) \end{aligned}$$

It is straightforward to check that the double category $\mathbb {P}\textrm{ath}\,\mathcal {C}$ is strict.

Suppose that the category $\mathcal {C}$ has an OFS $(\mathcal {L},\mathcal {R})$ and $(\varphi , \langle h_i \rangle _{1\le i \le m})$ is a fence, i.e., a double cell in $\mathbb {P}\textrm{ath}\,\mathcal {C}$. We may than choose to take the chosen factorization in $(\mathcal {L},\mathcal {R})$ for each of the cross arrows $h_i$, $h_i=r_{h_{i}}\circ \ell _{h_i}$. We can extend this to a factorization of the fence by observing that for all $i\in \{1, \cdots , m\}$, the commuting squares making up the fence can be factored as indicated (see example 3.5):

**Figure:**
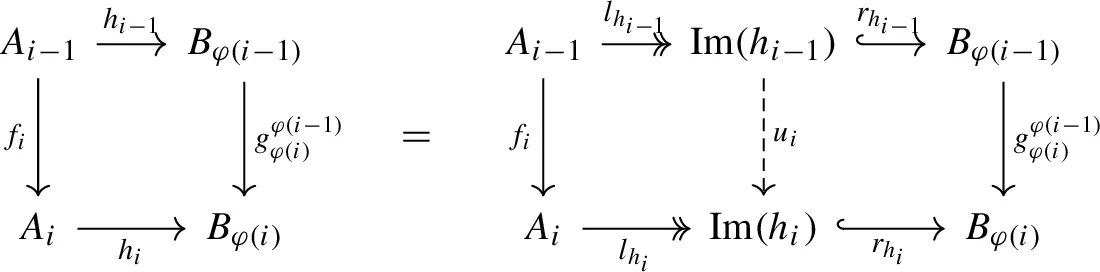

The morphisms $u_i$ exist and are unique because the factorisation $(\mathcal {L}, \mathcal {R})$ is orthogonal. Hence, every double cell $(\varphi , \langle h_i \rangle _{0\le i \le m})$ factors as the composition of a (left) double cell $(\varphi _l =\textrm{id}_{[m+1]}, \langle l_{h_i}\rangle _{1\le i\le m})$ from $\langle f_i\rangle _{1\le i\le m}$ to $\langle u_i\rangle _{1\le i\le m}$ and a (right) double cell $(\varphi _r= \varphi , \langle r_{h_i}\rangle _{0\le i\le m})$ from $\langle u_i\rangle _{1\le i\le m}$ to $\langle g_i\rangle _{1\le i\le n}$. For example, the double cell (5) factorises as follows.

**Figure:**
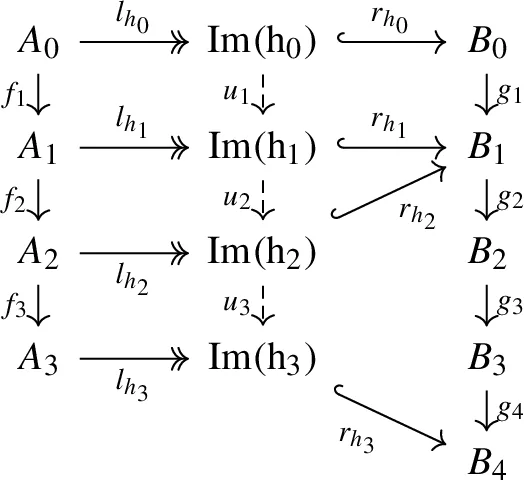

Note that double cells where the indexing map φ is the identity are called *neat fences* in [20].

This leads us to introduce the factorization system $(\mathcal {L}',\mathcal {R}')$ on the double cells of $\mathbb {P}\textrm{ath}\,\mathcal {C}$ where $\mathcal {L}'$ is the class of neat fences $(\textrm{id}_{[m+1]}, \langle l_{h_i}\rangle _{0\le i\le m})$ with cross arrows $h_i\in \mathcal {L}$ and $\mathcal {R}'$ is the class of fences $(\varphi , \langle l_{h_i}\rangle _{0\le i\le m})$ where all $h_i$ are in $\mathcal {R}$. In short, $\mathcal {L}'$ consists of neat $\mathcal {L}$-fences and $\mathcal {R}'$ consists of arbitrary $\mathcal {R}$-fences. It is clear that both $\mathcal {L}'$ and $\mathcal {R}'$ contain all horizontally invertible double cells, so in order to prove that $(\mathcal {L}',\mathcal {R}')$ is an OFS, it is sufficient to check the unique lifting property. So consider a horizontal commutative square of fences,
6where the arrows $\ell _i$ in the left fence are in $\mathcal {L}$ and the arrows $r_i$ in the right fence are in $\mathcal {R}$. We want to show that there is a unique fence *D* providing the diagonal lifting. First note that by the commutativity of the square, we have that for each i∈[m+1], there is a commutative square in $\mathcal {C}$,
7Since $$\ell _i\in \mathcal {L}$$ and $$r_{\varphi (i)}\in \mathcal {R}$$, there is a unique arrow $$d_i\in \mathcal {C}$$ making this diagram commute. Now these arrows together define a fence $$D=(\varphi ,\langle d_i\rangle _{0\le i\le m})$$ which forms a diagonal lifting for diagram (6). To show its uniqueness, let $$D'=(\theta ,\langle d_i'\rangle _{0\le i\le m})$$ be another lifting for this diagram. This implies that $$\theta =\varphi $$, because the top triangle commutes. So each arrow $$d_i'$$ is of the form $$d_i':A'_i\rightarrow B_{\varphi (i)}$$ and the way composition of fences is defined implies that $$d_i'$$ is a lifting for (7). Hence, $$d_i=d_i'$$ and hence $D^{\prime }=D$: (6) has a unique diagonal lifting and we conclude that $$(\mathcal {L}',\mathcal {R}')$$ is an OFS for the category of double cells and vertical arrows of $$\mathbb {P}\textrm{ath}\,\mathcal {C}$$.

Since vertical composition is defined by concatenation, it is clear that it preserves both the left and the right class of cells in this case. Furthermore, it preserves the chosen factorizations, so it is a strict map of orthogonal factorization systems.

#### Remark 3.12

We have presented examples of cell-wise factorization systems, suitably compatible with a factorization system on the arrows, on three common families of double categories.

1. Note that in all cases the source and target functors are strict morphisms of categories with an OFS. In light of Proposition 2.15 we will see that this is essential in both the internal category and the monadic approach toward double factorization systems. Note that in our examples the units functors are also strict. We will assume this throughout most of this paper, but one could develop the theory without this assumption, but one would loose some nice properties.
2. Vertical composition can be allowed to be a strict, lax, colax or pseudo-morphism of OFSs. We have seen here two examples of double categories with a strict vertical composition morphisms (quartets and paths), two with a lax vertical composition (relations and spans) and one with a colax vertical composition (copans).
3. These examples also lead us to ask whether there may be more than one factorization system on the double cells of a double category that extends a given OFS on the category of objects and arrows. We will discuss this in Proposition 5.5 and Theorem 5.7. In particular we will see that $\mathbb {R}\textrm{el}(\mathcal {C})$ has another DOFS with the same OFS on the arrows, and $\mathbb {S}\textrm{pan}(\mathcal {C})$ has at least three other factorization systems and we will discuss the morphisms between these factorization systems in Sects. 5 and 6.

### Internal Definition

We will define the notion of a pseudo double orthogonal factorization system (pseudo DOFS) (respectively a lax DOFS) as a pseudo-category internal to  (respectively ), with strict source, target and units assignment. (Note that one can define strict and colax DOFSs in a similar way, but we will leave this to the reader.) Recall from Sect. 2 the definition of these 2-categories of categories with an orthogonal factorization system, and the result (Proposition 2.15) that they have the pullbacks we need below.

#### Definition 3.13

Given lax internal functors $F,G:\mathbb {C} \rightarrow \mathbb {D}$, an *internal*, or *levelwise, natural transformation* $\eta :F \Rightarrow G$ consists of 2-cells $\eta _0:F_0 \Rightarrow G_0, \eta _1:F_1 \Rightarrow G_1$ satisfying the coherence conditions in Sect. 3.2 of [18].

#### Notation 3.14

Given a 2-category , we denote by  (respectively, , ) the 2-category of pseudo-categories internal to , internal pseudo (respectively, lax, oplax) functors and internal natural transformations. It is straightforward to show that these are indeed 2-categories.

We are now ready to give our formal definition of a double orthogonal factorization system. We want to mention here that an equivalent definition, but without chosen factorizations, was given in [9, Definition 57]. The terminology of *double orthogonal factorization system* was also used for a different concept, a ternary factorization system, in [6].

#### Definition 3.15

A strict (resp. lax, resp. colax, resp. pseudo) double orthogonal factorization system (DOFS) is a pseudo-category internal to the 2-category  (resp. ; resp. ; resp. ), such that $\operatorname {src}$, $\operatorname {tgt}$ and *i* are strict morphisms of orthogonal factorization systems.

#### Remark 3.16

1. According to this definition, a (lax/colax) DOFS $(\mathbb {L},\mathbb {R})$ on a double category $\mathbb {D}$ amounts to an OFS $(\mathcal {L}_0,\mathcal {R}_0)$ for the category $\mathcal {D}_0$ of objects and horizontal arrows (with chosen factorizations), along with left and right classes of double cells which form an OFS $(\mathcal {L}_1,\mathcal {R}_1)$ with respect to horizontal composition of cells (viewed as arrows of $\mathcal {D}_1$ in Definition 3.15) and chosen factorizations of the cells. These factorization systems are compatible, in the sense that source, target and units functors preserve the chosen factorizations. Furthermore, the (vertical) identity cell on a horizontal arrow is in the left class $\mathcal {L}_1$ (resp. right class $\mathcal {R}_1$) if and only if the arrow is in the left class $\mathcal {L}_0$ (resp. right class $\mathcal {R}_0$). In a pseudo DOFS vertical composition needs to preserve both the left and the right class of double cells. In a strict DOFS, we add to this that the vertical composition preserves the chosen factorizations of the cells. In the lax (resp. colax) case, only the right (resp. left) class is closed under vertical composition.
2. We are aware that it is more common to only require that the source and target arrows of an internal category diagram be stricter than the other structure arrows. However, doing this would lead to an undesirable level of weakness in the lax and colax cases: in the lax (resp. colax) case we would only have that the vertical identity cells on the right (resp. left) class of arrows are in the right (resp. left) class of double cells. Since we have not observed any relevant examples of this type, we would at least require that the identity cells in the left class are precisely those on the left class of arrows and similarly for the right class (potentially not requiring that the chosen factorizations of vertical identity cells consist of vertical identity cells). This would mean that the units functor *i* would only need to be a pseudo-morphism of OFSs. In Sect. 6 we will consider lax, colax and pseudo DOFS with *i* only a pseudo-morphism of OFSs because lifting along a double fibration may lead to a DOFS on the total double category where the chosen factorizations of vertical identities are not given in terms of vertical identities. Other than in that instance, we will assume that DOFSs in this paper have a units functor *i* that is a strict morphism of OFSs. We will indicate in the next sections how the results would need to be adjusted if one would work with a units functor that is only pseudo instead of strict. One feature of DOFSs with a strict units functor is that the complete structure of the DOFS is determined by its structure on the double cells of $\mathbb {D}$ as expressed in the following proposition.

#### Proposition 3.17

A pseudo DOFS $(\mathbb {L},\mathbb {R})$ on a double category $\mathbb {D}$ is equivalent to a pair of classes of double cells $(\mathcal {L},\mathcal {R})$ satisfying:

1. $\mathcal {L}$ and $\mathcal {R}$ are closed under horizontal composition, and contain all the horizontally invertible double cells.
2. $\mathcal {L}$ and $\mathcal {R}$ are closed under vertical composition.
3. For any double cell in either class, the unit cells on its source and target are also contained in the respective class; e.g., if $\alpha \in \mathcal {L}$, then $i(\operatorname {src}(\alpha ))\in \mathcal {L}$.
4. For every double cell φ there are chosen cells $\ell _\varphi \in \mathcal {L}$ and $r_\varphi \in \mathcal {R}$ such that
  **Figure:**
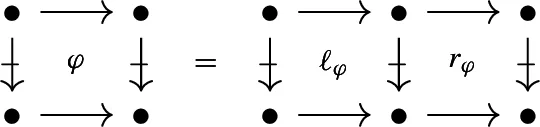
  - If two cells share the same source (respectively, target), then the corresponding chosen cells have the same source (respectively, target); e.g., if $\operatorname {src}(\alpha )=\operatorname {src}(\beta )$ then $\operatorname {src}(\ell _\alpha )=\operatorname {src}(\ell _\beta )$.
  - The chosen double cells that factor a vertical identity double cell are again vertical identity double cells.
5. (the lifting property) For any equation

**Figure:**
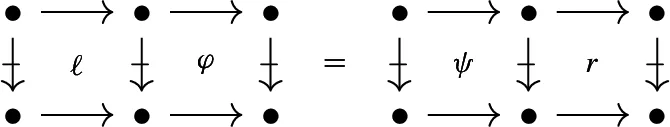

with $\ell \in \mathcal {L}$ and $r\in \mathcal {R}$, there is a unique cell θ that satisfies the two equations

**Figure:**
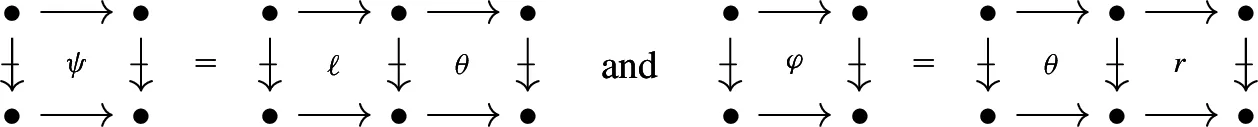

In the particular case that the cells $\ell , \varphi , \psi ,r$ are unit cells, then θ is also a unit cell.

A lax DOFS $(\mathbb {L},\mathbb {R})$ on a double category is equivalent to a pair of classes $(\mathcal {L}, \mathcal {R})$ satisfying the above conditions, but replacing (2) by:

$\textit{2}'.$: $\mathcal {R}$ is closed under vertical composition.

A colax DOFS $(\mathbb {L},\mathbb {R})$ on a double category $\mathbb {D}$ is equivalent to a pair of classes $(\mathcal {L}, \mathcal {R})$ satisfying the above conditions, but replacing (2) by:

$\textit{2}''.$: $\mathcal {L}$ is closed under vertical composition.

#### Proof

We first show that a double category $\mathbb {D}$ with a DOFS $(\mathbb {L}, \mathbb {R})$ satisfies the conditions of this Proposition, by taking $\mathcal {L}:= \mathcal {L}_1$ and $\mathcal {R}:= \mathcal {R}_1$.

Condition (1): Both $\mathcal {L}$ and $\mathcal {R}$ are closed under horizontal composition and contain the horizontally invertible cells by assumption.

Condition (2): This follows from the fact that the vertical composition is required to be a pseudo-morphism of OFSs.

Condition (3): This is a consequence of the fact that $\operatorname {src}$, $\operatorname {tgt}$, and *i* are strict morphisms of categories with orthogonal factorization systems (although it would be sufficient to assume that they were only pseudo-morphisms for this purpose).

Condition (4): Here we use the chosen factorization of the cells (viewed as arrows in $\mathcal {D}_1$) as a cell in $\mathcal {R}$ after a cell in $\mathcal {L}$. This is expressed in (4)(a). Condition (4)(b) follows from the fact that $\operatorname {src}$ and $\operatorname {tgt}$ are strict morphisms of OFSs. Condition (4)(c) follows from the fact that *i* is a strict morphism of OFSs.

Condition (5): The general case follows from the orthogonality of $(\mathcal {L}_1,\mathcal {R}_1)$, whereas the case of unit cells follows from the orthogonality of $(\mathcal {L}_0,\mathcal {R}_0)$.

Now, a double category $\mathbb {D}$ with a pair of classes of cells $(\mathcal {L},\mathcal {R})$ satisfying the conditions of the proposition gives rise to: the classes $\mathcal {L}_0$ and $\mathcal {R}_0$ of morphisms in $\mathcal {D}_0$ that consist of the horizontal morphisms whose associated unit cells lie in $\mathcal {L}$ and $\mathcal {R}$, respectively; and the classes $\mathcal {L}_1:= \mathcal {L}$ and $\mathcal {R}_1:= \mathcal {R}$, of $\mathcal {D}_1$.

The pair $(\mathcal {L}_1,\mathcal {R}_1)$ is an OFS for $\mathcal {D}_1$ because $\mathcal {L}$ and $\mathcal {R}$ are both closed under horizontal composition, both have all horizontally invertible cells, condition (4)(a) gives the chosen factorizations and condition (5) gives the lifting property.

We now show that the pair $(\mathcal {L}_0,\mathcal {R}_0)$ is an OFS for $\mathcal {D}_0$. Indeed, from the fact that horizontal composition of unit cells is again a unit, and $\mathcal {L}$ and $\mathcal {R}$ are closed under horizontal composition, it follows that $\mathcal {L}_0$ and $\mathcal {R}_0$ are closed under composition. They contain all isomorphisms in $\mathcal {D}_0$ because $\mathcal {L}$ and $\mathcal {R}$ contain all horizontally invertible cells. Every morphism *f* in $\mathcal {D}_0$ factors as a morphism in $\mathcal {L}_0$ followed by a morphism in $\mathcal {R}_0$ by conditions (4)(a) and (4)(c), and we take these factorizations as the chosen ones. The lifting property in $(\mathcal {L}_0,\mathcal {R}_0)$ follows from the last part of condition (5).

The functors $$\operatorname {src}{}$$ and $$\operatorname {tgt}$$ are strict morphisms of OFSs by condition (3) and condition (4)(b). The units functor *i* is a strict morphism of OFSs by the way the pairs $$(\mathcal {L}_0,\mathcal {R}_0)$$ and $$(\mathcal {L}_1,\mathcal {R}_1)$$ are defined together with conditions (4)(b) and (4)(c) and our choice of factorizations. The vertical composition is a pseudo-morphism of OFSs by condition (2). The proof of the equivalence for the lax (respectively, colax) DOFS is exactly the same, but noticing that axiom ( $2^{\prime }$) (respectively, axiom ( $2^{\prime \prime }$)) implies that vertical composition is a lax (respectively, colax) morphism of categories with orthogonal factorization system. □

We now present a result giving an equivalent formulation of Condition (5). This result will serve as a supporting tool in examples in Sect. 3.4.

#### Proposition 3.18

Under the remaining assumptions of Proposition 3.17, condition (5) may be replaced by the following equivalent condition:

$\textit{5}'.$: For any equation

**Figure:**
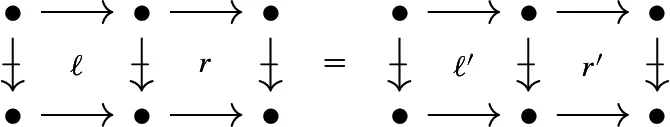

with $\ell , \ell ' \in \mathcal {L}$ and *r*, $r'\in \mathcal {R}$, there is a unique horizontally invertible cell θ that satisfies the following two equations

**Figure:**
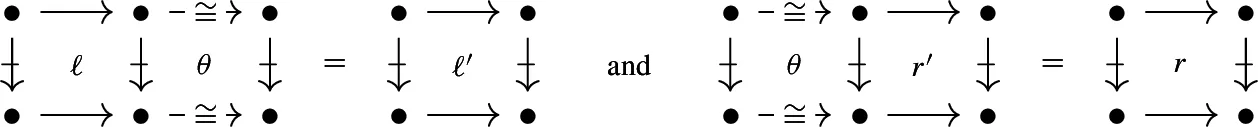

When $\ell ,r,\ell ', r'$ are unit cells, θ is also a unit cell.

#### Proof

The equivalence follows from standard results on orthogonal factorization systems. The implication $\left(5\right)\Rightarrow \left(5^{\prime }\right)$ follows from the fact that uniqueness of factorizations up to unique isomorphism is a special case of the lifting property (see, for instance, Sect. 2.2 of [3]). Conversely, $\left(5^{\prime }\right)\Rightarrow \left(5\right)$ follows from the fact that, in an OFS, the lifting property is a consequence of the existence of factorizations of every morphism as a morphism in the left class followed by a morphism in the right class, the closure of both classes under composition, and the uniqueness of factorizations up to unique isomorphism. □

The following observation shows that the cell θ in Proposition 3.18 is a globular cell if the horizontal sources and targets coincide in two different factorizations.

#### Observation 3.19

In Proposition 3.18, consider the particular case

**Figure:**
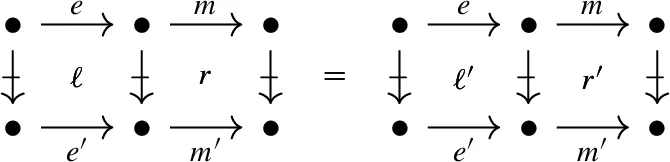

where ℓ and $\ell^{\prime }$ have the same source and target, and *r* and $r^{\prime }$ have the same source and target. Now consider the equations $$\operatorname {src}(\theta )\circ e = e$$ and $$m\circ \operatorname {src}(\theta )=m$$ for the unique cell θ in Proposition 3.18. The orthogonal property in the orthogonal factorization system in $$\mathcal {C}_0$$ implies that $$src(\theta ) = \operatorname {id}$$. Analogously $$\operatorname {tgt}(\theta ) = \operatorname {id}$$. This asserts that the unique cell arising from two different factorizations of the same cell, with the same sources and targets, is horizontally globular.

### Examples of DOFS on Double Categories Constructed from 2-Categories

In this subsection, we present an example of a DOFS in the double category of quintets on , together with an example in the double category of paths over the double category of quintets on a 2-category $\mathcal {B}$.

#### Example 3.20

*(The quintet double category on the 2-category* , *)*. Our aim is to show that the class $\mathcal {L}$ that consists of cells with final functors in their horizontal arrows and the class $\mathcal {R}$ that consists of cells that are natural isomorphism with discrete fibrations in their horizontal arrows is a DOFS on . First of all, final functors and discrete fibrations form an OFS in the category of categories [21]. Thanks to that fact, it is clear that both $\mathcal {L}$ and $\mathcal {R}$ are closed under horizontal composition. Also, by definition, the classes $\mathcal {L}$ and $\mathcal {R}$ contain all the horizontally invertible cells. Now, we want to show that the axioms (1), (2), (3), and (4) in Proposition 3.17 and axiom (5’) in Proposition 3.18 hold for $\mathcal {L}$ and $\mathcal {R}$. It is clear that axioms (1), (3), and (4) hold. We will focus on axiom (2) and axiom (5’).

We start by proving that Axiom (2) holds. We wish to factor an arbitrary cell α into two cells

**Figure:**
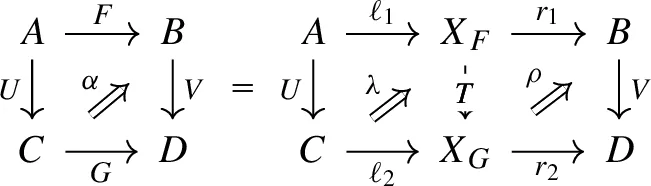

where $$\lambda \in \mathcal {L}$$ and $$\rho \in \mathcal {R}$$.

Step 1 We choose $\ell _1,r_1,\ell _2,r_2$ using the comprehensive factorization system as in [22] or on page 74 of [23].
Step 2 We define the functor *T* as follows: For an object (b,[h:Fa→b]) in $X_F$ , $T(b,[h]):= (Vb, [Vh\circ \alpha _a])$ in $X_G$, and for a morphism *t* in $X_F$, T(t):=V(t).
Step 3 We define λ and ρ. Observe that for an object a∈A, $\ell _2U(a) = (GUa, [\textrm{id}])$, and $T\ell _1(a) = T(Fa, [\textrm{id}]) = (VFa, [\alpha _a])$. We define $\lambda _a$ to be the morphism $\alpha _a: (GUA, [\operatorname {id}]) \rightarrow (VFa,[\alpha _a])$. The natural isomorphisms $\rho _{(b,[h])}$ are defined to be the identities $\operatorname {id}_{b}:r_2T(b,[h])=Vb \rightarrow Vr_1(b,[h]) =Vb$. It is clear that $\lambda \in \mathcal {L}$, $\rho \in \mathcal {R}$, and $\alpha = \lambda *\rho$.

Next, we prove that Axiom (5’) holds. Assume there are two different ways to factor a cell

**Figure:**
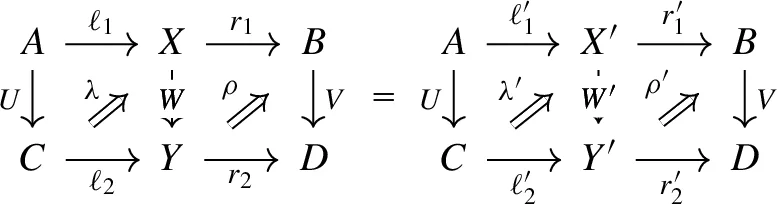

We wish to define a horizontally invertible cell

**Figure:**
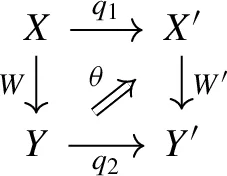

such that $\theta *\lambda = \lambda '$ and $\rho '*\theta = \rho$. By the orthogonal property of an OFS, there exist isomorphisms $q_1:X\rightarrow X'$ and $q_2:Y\rightarrow Y'$ that make the following diagrams commute

**Figure:**
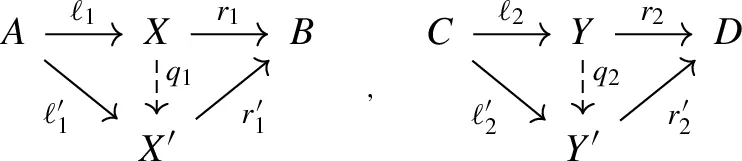

So, we wish to find a natural isomorphism $\theta :q_2W\rightarrow W'q_1$. First, observe that

**Figure:**
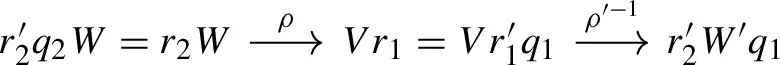

Since $$r_2'$$ is a discrete fibration, there is a unique cartesian lifting $$\theta :q_2W\rightarrow W'q_1$$ of $$\rho '^{-1}\rho $$. It is a natural isomorphism because $\rho^{\prime }$ and ρ are natural isomorphisms and $$r_2'$$ is a discrete fibration. Now, by definition, $$r_2'(\theta ) = \rho '^{-1}\rho $$, which implies that $$\rho ' r_2'(\theta ) = \rho $$, i.e., $$\rho '*\theta = \rho $$. Finally, we wish to prove that $$\theta *\lambda = \lambda '$$, i.e., $$\theta q_2(\lambda ) = \lambda '$$. We know that $$\rho r_2(\lambda ) = \rho *\lambda =\rho '*\lambda ' = \rho 'r_2'(\lambda ')$$. Then, $$r_2'(\theta )r_2(\lambda )=\rho '^{-1}\rho r_2(\lambda ) = r_2'(\lambda ')$$. Again, since $$r_2'$$ is a discrete fibration, there exists a unique morphism *s* such that $$r_2'(s) = r_2'(\lambda )$$. We also have that $$ \rho \rho '^{-1}r_2'(s) = r_2'(\lambda ')$$, and by the cartesian property $$\theta s = \lambda '$$. Observe that $$r_2'q_2(\lambda ) = r_2(\lambda )$$, by definition of $$q_2$$; then, by uniqueness of *s*, $$s=q_2(\lambda )$$, which concludes the example.

#### Example 3.21

*(The double category of paths in* $\mathbb {Q}\mathcal {B}$, $\mathbb {P}\textrm{ath}\mathbb {Q}\mathcal {B}$*)* In Example 3.11 we described a DOFS on the double category of paths in a category $\mathcal {C}$; we refer the reader to that example for a clearer understanding of the notation. Here, we extend the DOFS to a more general setting. For a 2-category $\mathcal {B}$, the double category $\mathbb {P}\textrm{ath}\mathbb {Q}\mathcal {B}$ of paths in the double category of quintets $\mathbb {Q}\mathcal {B}$ has the following structure: its objects are those of $\mathcal {B}$; its horizontal arrows are the 1-morphisms in $\mathcal {B}$; its vertical arrows are paths of 1-morphisms in $\mathcal {B}$; and the cells

**Figure:**
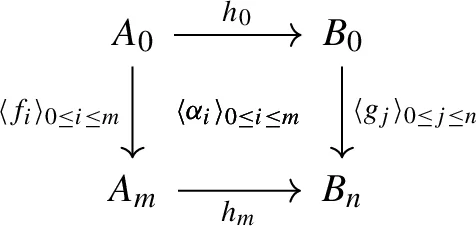

are pairs $(\varphi ,\langle \alpha _i\rangle _{0\le i\le m})$, where $\varphi :[m+1]\rightarrow [n+1]$ is an order preserving function with φ(0)=0 and φ(m)=n; and for every i∈[m+1], $\alpha _i$ is a double cell in $\mathbb {Q}\mathcal {B}$ of the form

**Figure:**
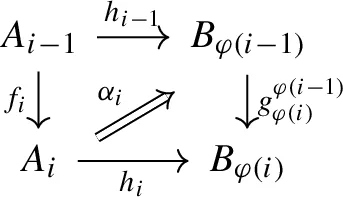

The double cells in $\mathbb {P}\textrm{ath}(\mathbb {Q}\mathcal {B})$ are called *fences*. Vertical composition of fences is defined by concatenation, while horizontal composition is induced by composition of arrows and double cells in $\mathbb {Q}\mathcal {B}$, following the threads of the cross arrows.

Suppose that the double category $\mathbb {Q}\mathcal {B}$ has a pair of OFSs, $(\mathcal {L}_0',\mathcal {R}_0')$ on $\mathbb {Q}\mathcal {B}_0=\mathcal {B}$, $(\mathcal {L}_1', \mathcal {R}_1')$ on $\mathbb {Q}\mathcal {B}_1$ with choices of factorizations such that $\operatorname {src}$, $\operatorname {tgt}$, and *i* are strict morphisms of OFSs. Now consider a fence $(\varphi , \langle \alpha _i\rangle _{0\le i\le m})$. Then, the cells $\alpha _i$ have chosen factorizations,

**Figure:**
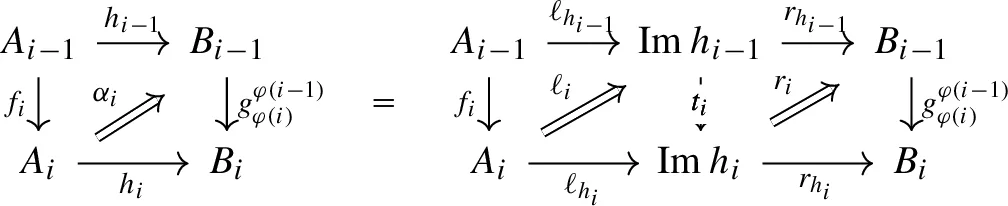

This provides a factorization for the fence $(\varphi , \langle \alpha _i\rangle _{0\le i\le m})$ and induces a DOFS $(\mathbb {L},\mathbb {R})$ on $\mathbb {P}\textrm{ath}\mathbb {Q}\mathcal {B}$, with $(\mathcal {L}_0,\mathcal {R}_0)=(\mathcal {L}_0',\mathcal {R}_0')$. The class $\mathcal {L}_1$ consists of the neat fences $(\operatorname {id}_{[m+1]},\langle \ell _i\rangle _{0\le i\le m} )$, where the cells $\ell _i$ are in the class $\mathcal {L}_1'$. Similarly, the class $\mathcal {R}$ consists of the fences $(\varphi , \langle r_i\rangle _{0\le i \le m})$, where the cells $r_i$ are in the class $\mathcal {R}_1'$. Then, since every cell in $\mathbb {P}\textrm{ath}\mathbb {Q}\mathcal {B}$ can be expressed as a vertical concatenation of cells that are induced by cells in $\mathbb {Q}(\mathcal {B})$, and by the conditions on the original OFSs, it is easy to check that the pair $(\mathcal {L}_1,\mathcal {R}_1)$ satisfies the axioms of Proposition 3.17. Therefore $(\mathcal {L},\mathcal {R})$ constitutes a pseudo DOFS on the double category $\mathbb {P}\textrm{ath}\mathbb {Q}\mathcal {B}$.

#### Remark 3.22

It may be tempting to compare the DOFS on a double category of quintets with the 2-dimensional OFS introduced by Stefan Milius [24, Definition 7.3]. However, the goals of these two types of systems are different: our systems provide a notion of factorization and hence an image for double cells, whereas the goal of Milius’ work is to provide a weakened version of orthogonal factorization systems for 2-categories. It is not obvious how to construct a left and right class of 2-cells from Milius’ concept. Also, the diagonal arrow goes in the wrong direction to form the image of a 2-cell.

## Monadicity of Double Orthogonal Factorization Systems

In this section, we prove a monadicity result for all types of double orthogonal factorization systems (strict, pseudo, lax and colax), achieving double categorical generalizations of the fundamental monadicity result of Theorem 2.5 and providing us with natural notions of morphisms and 2-cells between such DOFSs. To reach this, we first prove a conceptually important general monadicity result for categories internal to algebras, Theorem 4.3. This theorem is particularly helpful in our double categorical context, but is more widely applicable.

We then merge two flavours of DOFS (lax and pseudo, or colax and pseudo) together via $\mathcal {F}$-category theory, also known as enhanced 2-category theory (see [25]). The right $\mathcal {F}$-categorical monad to consider to achieve our monadicity theorem is a generalization of the squaring 2-monad on  to the double categorical setting.

The results of this section can be thought of as giving a monadic definition of (lax or colax) double orthogonal factorization systems and showing that this definition is equivalent to the internal one we presented in Sect. 3.

Aiming at the general monadicity theorem, we prove the following lemma. This is a generalization of Proposition 2.15.

### Lemma 4.1

Let  be a 2-category and let  be a 2-monad whose underlying 2-functor preserves pullbacks. The 2-category  (respectively, ) of normal pseudo-algebras and lax morphisms (respectively, pseudo-morphisms) between them has all pullbacks of strict morphisms along strict morphisms that exist in  (and these pullback morphisms are again strict).

### Proof

Let $(C_0,Q_0:T(C_0)\rightarrow C_0,\alpha _{\mu ,0})$, $(C_1,Q_1:T(C_1)\rightarrow C_1,\alpha _{\mu ,1})$ and $(C_2,Q_2:T(C_2)\rightarrow C_2,\alpha _{\mu ,2})$ be normal pseudo-algebras. Furthermore, let $f:C_1\rightarrow C_0$ and $g:C_2\rightarrow C_0$ be strict morphisms of normal pseudo-algebras, and assume that the pullback $C_1\times _{C_0}C_2$ of *f* and *g* exists in ,

**Figure:**
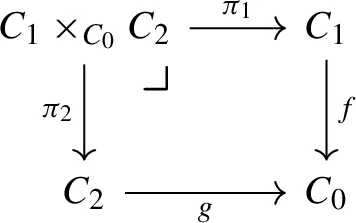

We prove that $(C_1\times _{C_0}C_2,Q_1\times _{Q_0}Q_2:T(C_1\times _{C_0}C_2)\rightarrow C_1\times _{C_0}C_2,\alpha _{\mu ,1}\times _{\alpha _{\mu ,0}}\alpha _{\mu ,2})$ is the pullback of *f* and *g* in .

Since *T* preserves pullbacks, we can choose $T(C_1)\times _{T(C_0)} T(C_2)$ to be $T(C_1\times _{C_0}C_2)$. Then the pullback square of $C_1\times _{C_0}C_2$ with its image under *T*, connected by $Q_0,Q_1,Q_2$ induce a morphism $Q_1\times _{Q_0}Q_2:T(C_1\times _{C_0}C_2)\rightarrow C_1\times _{C_0}C_2$, by the universal property of the pullback. Analogously, by the 2-dimensional universal property of the pullback, we can induce an invertible 2-cell

**Figure:**
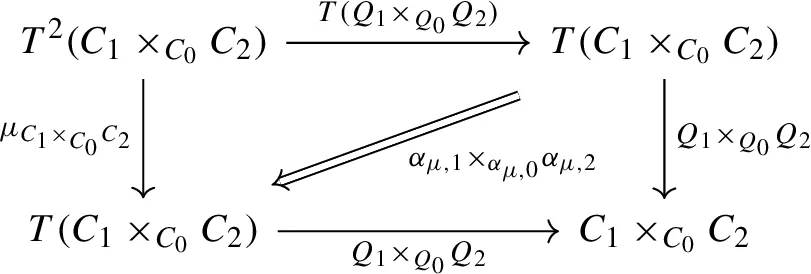

again using that *f* and *g* are strict morphisms of normal pseudo-algebras. It is straightforward to show that $(C_1\times _{C_0}C_2,Q_1\times _{Q_0}Q_2:T(C_1\times _{C_0}C_2)\rightarrow C_1\times _{C_0}C_2,\alpha _{\mu ,1}\times _{\alpha _{\mu ,0}}\alpha _{\mu ,2})$ is a normal pseudo-algebra. We then notice that $\pi _1$ and $\pi _2$ are strict morphisms of normal pseudo-algebras.

Consider now $(C_3,Q_3:T(C_3)\rightarrow C_3,\alpha _{\mu ,3})$ a normal pseudo-algebra together with lax morphisms $(h,\varphi ^h):(C_3,Q_3,\alpha _{\mu ,3})\rightarrow (C_1,Q_1,\alpha _{\mu ,1})$ and $(k,\varphi ^k):(C_3,Q_3,\alpha _{\mu ,3})\rightarrow (C_2,Q_2,\alpha _{\mu ,2})$ between normal pseudo-algebras such that $(f,\operatorname {id})\circ (h,\varphi ^h)=(g,\operatorname {id})\circ (k,\varphi ^k)$. Then by the universal property of the pullback, these data induce both a morphism $\langle h,k\rangle :C_3\rightarrow C_1\times _{C_0}C_2$ and a 2-cell

**Figure:**
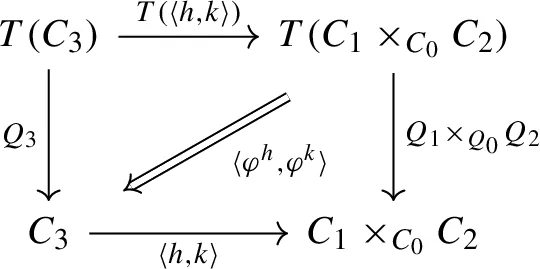

Notice that the morphism on top is indeed $T(\langle h,k\rangle )$ by the uniqueness part of the universal property of the pullback. It is straightforward to check that $(\langle h,k \rangle ,\langle \varphi ^h,\varphi ^k\rangle )$ is a lax morphism of normal pseudo-algebras, and that it is the unique lax morphism that fulfills the 1-dimensional universal property of the pullback in . Notice that if both $\varphi ^h$ and $\varphi ^k$ are iso, so is $\langle \varphi ^h,\varphi ^k\rangle$. So pseudo-morphisms between normal pseudo-algebras induce a pseudo-morphism.

Taking then 2-cells  and  between lax morphisms of algebras such that $f*\sigma =g*\tau$, we can analogously induce a 2-cell  between lax morphisms of algebras, which is straightforwardly shown to be the unique 2-cell that fulfills the 2-dimensional universal property of the pullback in . □

### Remark 4.2

The result of Lemma 4.1 could also be obtained as a consequence of Proposition A.2 in [26] which proves in the context of models for enhanced $\mathcal {F}$-sketches that all limits are (*s*, *s*)-created (where *s* stands for the strict, or tight, morphisms), and furthermore all tight limits are (*s*, *w*)-created.

We now prove a general monadicity theorem which provides a way to extend a 2-monad on a 2-category $\mathcal {K}$ to canonical 2-monads on the 2-categories of categories internal to $\mathcal {K}$ with either lax, oplax or pseudo-functors, and shows how the 2-categories of algebras for the latter are isomorphic to the corresponding 2-categories of internal categories in algebras for the former. This will be particularly useful for us in the context of double categories.

### Theorem 4.3

Let  be a 2-category and let  be a 2-monad whose underlying 2-functor preserves pullbacks. Then *T* induces 2-monads
such that
where  (respectively, ) is the full sub-2-category of  (respectively, ) on those pseudo-categories whose source, target and identity assignment are strict morphisms between normal pseudo-algebras.

### Proof

Given *C* a pseudo-category

**Figure:**
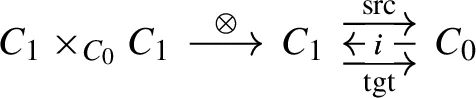

in , we define $\overline{T}_{{{\,\textrm{opl}\,}}}(C)$ to be the pseudo-category

**Figure:**
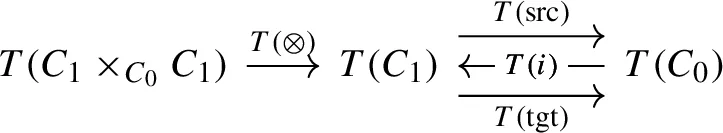

choosing ${T(C_1)\times _{T(C_0)}T(C_1)}$ to be $T({C_1\times _{C_0}C_1})$ (and similarly with iterated pullbacks), thanks to the fact that *T* preserves pullbacks. Its associator is just T(Φ), where Φ is the associator of *C*, and its left and right unitors are T(λ) and T(ρ) respectively, where λ and ρ are the left and right unitors of *C*. It is straightforward to show that this gives indeed a pseudo-category.

Given then a normal internal oplax functor $(F_0:C_0\rightarrow D_0,F_1:C_1\rightarrow D_1,\varphi )$, we send it to the normal internal oplax functor $(T(F_0),T(F_1),T(\varphi )):\overline{T}_{{{\,\textrm{opl}\,}}}(C)\rightarrow \overline{T}_{{{\,\textrm{opl}\,}}}(D)$. The required axioms are all images under *T* of the corresponding axioms for $(F_0,F_1,\varphi )$. And given an internal natural transformation $(\alpha _0,\alpha _1)$ between normal internal oplax functors, we send it to the internal natural transformation $(T(\alpha _0),T(\alpha _1))$. Again, all the required axioms are images under *T* of the corresponding axioms for $(\alpha _0,\alpha _1)$. It is straightforward to check that $\overline{T}_{{{\,\textrm{opl}\,}}}$ is a 2-functor.

We then define a unit  and a multiplication $\overline{\mu }:\overline{T}_{{{\,\textrm{opl}\,}}}^2\rightarrow \overline{T}_{{{\,\textrm{opl}\,}}}$ for $\overline{T}_{{{\,\textrm{opl}\,}}}$ as follows. Given , we define $\overline{\mu }_C$ to be the normal internal strict functor $(\mu _{C_0}:T^2(C_0)\rightarrow T(C_0),\mu _{C_1}:T^2(C_1)\rightarrow T(C_1),\operatorname {id})$, thanks to the naturality of μ. Notice that $\mu _{C_1}\times _{\mu _{C_0}}\mu _{C_1}$ coincides with $\mu _{C_1\times _{C_0}C_1}$ by the universal property of the pullback, and we can thus choose $\operatorname {id}$ as structure 2-cell in both

**Figure:**
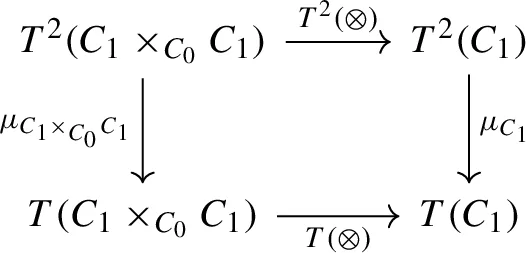

and

**Figure:**
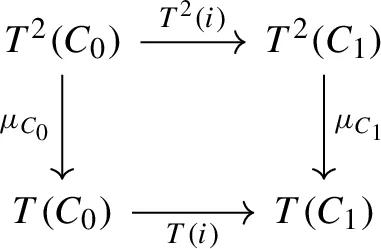

by naturality of μ, making $\overline{\mu }_C$ into an internal strict functor between internal pseudo-categories (the required axioms hold thanks to the 2-naturality of μ). 2-Naturality of μ then also guarantees that $\overline{\mu }$ is 2-natural.

Given , we then analogously define $\overline{\eta }_C$ to be the normal internal strict functor $(\eta _{C_0}:C_0\rightarrow T(C_0),\eta _{C_1}:C_1\rightarrow T(C_1),\operatorname {id})$. 2-Naturality of η guarantees that $\overline{\eta }$ is well defined and 2-natural.

It is straightforward to check that $(\overline{T}_{{{\,\textrm{opl}\,}}},\overline{\eta },\overline{\mu })$ satisfies the axioms for a 2-monad on , thanks to the corresponding ones for $(T,\eta ,\mu )$ and the fact that both $\overline{\eta }$ and $\overline{\mu }$ have internal strict functors as components, with structure 2-cells given by identities.

Notice then that the 2-monad $\overline{T}_{{{\,\textrm{opl}\,}}}$ on  restricts to a 2-monad $\overline{T}_{{{\,\textrm{ps}\,}}}$ on . Indeed if $(F_0:C_0\rightarrow D_0,F_1:C_1\rightarrow D_1,\varphi )$ is a normal internal pseudo-functor, which means that φ is invertible, then also T(φ) is invertible and

$$\begin{aligned} (T(F_0),T(F_1),T(\varphi )):\overline{T}_{{{\,\textrm{opl}\,}}}(C)\rightarrow \overline{T}_{{{\,\textrm{opl}\,}}}(D) \end{aligned}$$

is a normal internal pseudo-functor. Moreover, both $\overline{\eta }$ and $\overline{\mu }$ have as components internal strict functors.

We now prove that
So consider $(C,Q:\overline{T}_{{{\,\textrm{opl}\,}}}(C)\rightarrow C,(\alpha _{\mu ,0},\alpha _{\mu ,1}))$ a normal pseudo-algebra for $\overline{T}_{{{\,\textrm{opl}\,}}}$. Here, $C=(C_0,C_1,\operatorname {src},\operatorname {tgt},i,\Phi ,\lambda ,\rho )$, with Φ the associator and λ and ρ the left and right unitor respectively, and $(\alpha _{\mu ,0},\alpha _{\mu ,1}):Q\circ \overline{T}_{{{\,\textrm{opl}\,}}}Q\Rightarrow Q\circ \mu _C$ is the internal (levelwise) natural transformation which gives the structure 2-cell of a normal pseudo-algebra. We write $Q=(Q_0,Q_1,\varphi ^Q)$, with $\varphi ^Q$ the structure 2-cell for the internal normal oplax functor *Q*. The following diagrams help visualize the situation:

**Figure:**
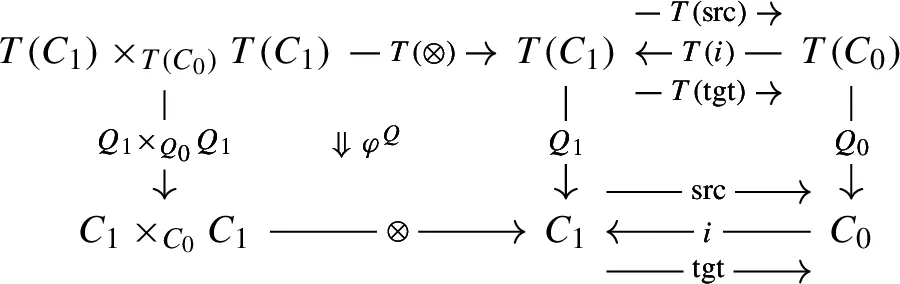

**Figure:**
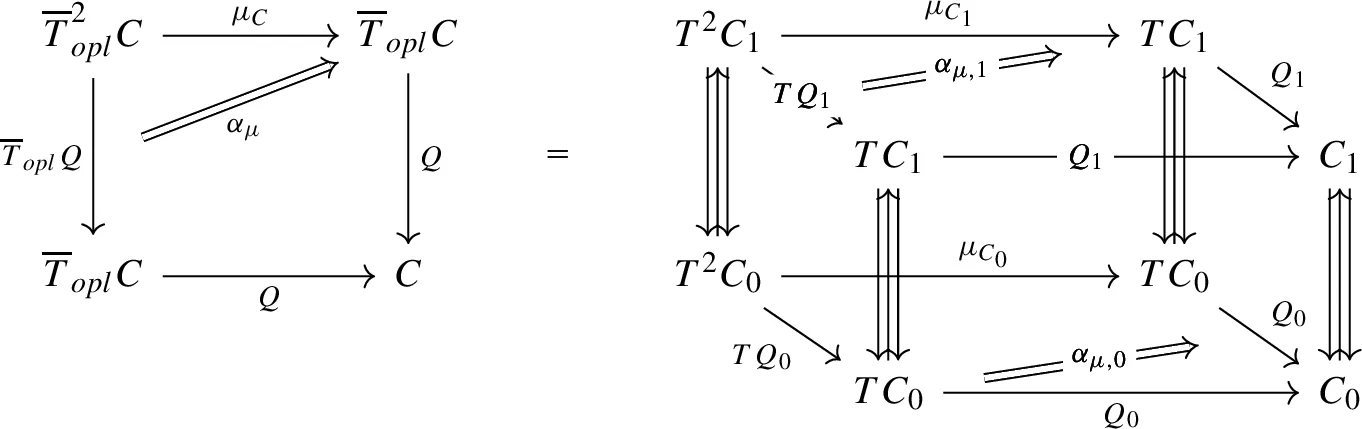

It is straightforward to show that:

- The 2-cells $\alpha _{\mu ,0}:Q_0\circ TQ_0\Rightarrow Q_0\circ \mu _{C_0}$ and $\alpha _{\mu ,1}:Q_1\circ TQ_1\Rightarrow Q_1\circ \mu _{C_1}$ give $(C_0,Q_0:T(C_0)\rightarrow C_0,\alpha _{\mu ,0})$ and $(C_1,Q_1:T(C_1)\rightarrow C_1,\alpha _{\mu ,1})$ respectively the structure of normal pseudo-algebras;
- The commutative diagram

**Figure:**
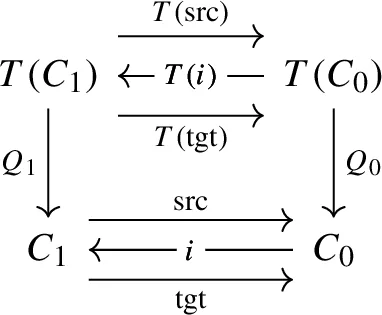

can be viewed as establishing that $\operatorname {src},\operatorname {tgt}:C_1\rightarrow C_0$ and $i:C_0\rightarrow C_1$ are strict morphisms of *T*-algebras.

By Lemma 4.1, $\operatorname {src}$ and $\operatorname {tgt}$ have a pullback in , given by

$$\begin{aligned} (C_1\times _{C_0} C_1, Q_1\times _{Q_0}Q_1:T(C_1\times _{C_0} C_1)\rightarrow C_1\times _{C_0} C_1, \alpha _{\mu ,1}\times _{\alpha _{\mu ,0}} \alpha _{\mu ,1}). \end{aligned}$$

And analogously, $\operatorname {src}$ and $\operatorname {tgt}$ have all iterated pullbacks. We can then use the vertical composition ⊗ of *C* and $\varphi ^Q$ to produce a vertical composition for $(Q_0,Q_1)$. It is indeed straightforward to show that the 2-cell

**Figure:**
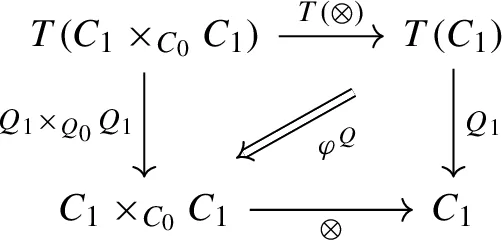

is a lax morphism of normal pseudo-algebras, and then that

$$\begin{aligned} (Q_0,Q_1,(\operatorname {src},\operatorname {id}),(\operatorname {tgt},\operatorname {id}),(i,\operatorname {id}),(\otimes ,\varphi ^Q),\Phi ) \end{aligned}$$

is a pseudo-category internal to .

If we now start from a pseudo-category internal to  with source, target and identity strict morphisms between normal pseudo-algebras, we can produce a normal pseudo-algebra for $\overline{T}_{{{\,\textrm{opl}\,}}}$. Indeed we can first produce a pseudo-category *C* internal to . Then the structure 2-cell $\varphi ^\otimes$ of the vertical composition ⊗ extends $Q_0$ and $Q_1$ to an internal oplax functor $Q:\overline{T}_{{{\,\textrm{opl}\,}}} (C)\rightarrow C$, also thanks to the associator Φ being a 2-cell between algebras. Finally, $\alpha _{\mu ,0}$ and $\alpha _{\mu ,1}$ form an internal natural transformation thanks to the fact that $\operatorname {src},\operatorname {tgt}$, and *i* are strict morphisms of normal pseudo-algebras. So we produce a normal pseudo-algebra for $\overline{T}_{{{\,\textrm{opl}\,}}}$. It is straightforward to see that the two constructions we have given are inverses of each other, giving a bijection on objects between  and .

Consider now a lax morphism

$$\begin{aligned} (C,Q:\overline{T}_{{{\,\textrm{opl}\,}}}(C)\rightarrow C,(\alpha _{\mu ,0},\alpha _{\mu ,1}))\rightarrow (D,R:\overline{T}_{{{\,\textrm{opl}\,}}}(D)\rightarrow D,(\beta _{\mu ,0},\beta _{\mu ,1})) \end{aligned}$$

between normal pseudo-algebras for $\overline{T}_{{{\,\textrm{opl}\,}}}$. This consists of an oplax functor $(F_0,F_1,\lambda ):C\rightarrow D$ internal to  equipped with a structure internal natural transformation $(\xi _0,\xi _1)$ where

**Figure:**
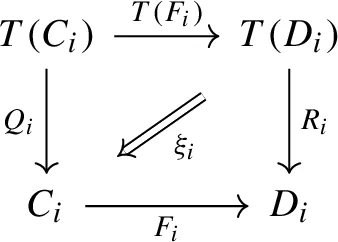

for i=1,2. This gives us a lax morphism of normal pseudo-algebras

$$\begin{aligned} (F_0,\xi _0):(C_0,Q_0:T(C_0)\rightarrow C_0,\alpha _{\mu ,0})\rightarrow (D_0,R_0:T(D_0)\rightarrow D_0,\beta _{\mu ,0}) \end{aligned}$$

and analogously $(F_1,\xi _1):Q_1\rightarrow R_1$. We can thus associate to $((F_0,F_1,\lambda ),(\xi _0,\xi _1))$ the oplax functor internal to  given by

$$\begin{aligned} ((F_0,\xi _0):Q_0\rightarrow R_0, (F_1,\xi _1):Q_1\rightarrow R_1,\lambda ). \end{aligned}$$

Indeed, it is straightforward to show that

**Figure:**
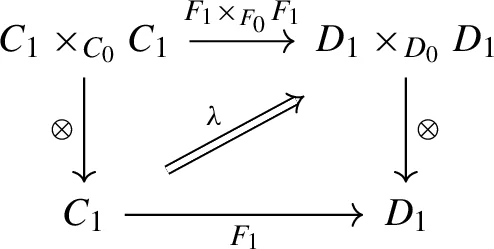

is a 2-cell between lax morphisms of normal pseudo-algebras and that the structure defined above is an oplax functor internal to . Notice that, for the latter, the required axioms are equalities of 2-cells between normal pseudo-algebras, which can then be checked in  using the underlying 2-cells. The following diagram helps visualize the situation:

**Figure:**
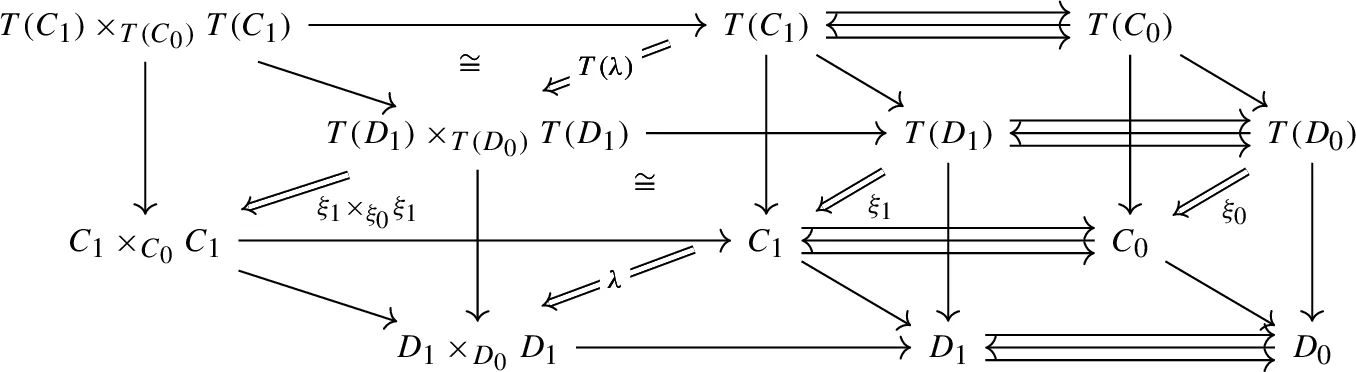

Starting now from an oplax functor $((F_0,\xi _0):Q_0\rightarrow R_0,(F_1,\xi _1):Q_1\rightarrow R_1,\lambda )$ internal to  between pseudo-categories with source, target and identity strict morphisms between normal pseudo-algebras, we produce a lax morphism between normal pseudo-algebras for $\overline{T}_{{{\,\textrm{opl}\,}}}$. It is straightforward to show that $(F_0,F_1,\lambda )$ is a normal oplax functor internal to , thanks to the fact that $((F_0,\xi _0),(F_1,\xi _1),\lambda )$ is a normal oplax functor and λ is a 2-cell between normal pseudo-algebras. Then $(\xi _0,\xi _1)$ is an internal natural transformation that makes $((F_0,F_1,\lambda ),(\xi _0,\xi _1))$ into a lax morphism between normal pseudo-algebras for $\overline{T}_{{{\,\textrm{opl}\,}}}$. It is straightforward to show that the two assignments we have described are functorial and inverses of each other, giving the 1-dimensional part of
We show that the 2-dimensional part holds as well. So consider a 2-cell between normal pseudo-algebras for $\overline{T}_{{{\,\textrm{opl}\,}}}$. This is an internal natural transformation  that satisfies some axioms. It is straightforward to see that $\gamma _0$ is a 2-cell from the lax morphism $(F_0,\xi _0)$ of normal pseudo-algebras to the corresponding $(G_0,\xi '_0)$, and analogously for $\gamma _1$. One can then see that sending $(\gamma _0,\gamma _1)$ to $(\gamma _0,\gamma _1)$ and vice versa gives an isomorphic 2-functor, exhibiting thus the isomorphism of 2-categories
Finally, it is straightforward to see that this isomorphism of 2-categories restricts to
Indeed the structure 2-cell $\varphi ^Q$ of the internal oplax functor *Q* and the structure 2-cell $\varphi ^\otimes$ of the lax morphism of normal pseudo-algebras which gives the vertical composition for $(Q_0,Q_1)$ correspond to each other, and if one is invertible then the other one is invertible. Analogously for $\xi _0,\xi _1$ and λ in the correspondence between morphisms.

So we have established the required isomorphisms of 2-categories. □

### Remark 4.4

The intuitive idea behind the proof of Theorem 4.3 is that, interestingly, objects, morphisms, and 2-cells of the 2-categories
and
respectively, are all presented by the same underlying data, grouped in two different ways.

### Remark 4.5

For the reader interested in the situation where we do require the units map *i* to be only a pseudo (or even lax) morphism of algebras, the proof above can be extended to the case with internal oplax functors between the pseudo-categories of pseudo-algebras which are either pseudo (resp. oplax) on the units as well.

We can now apply this general monadicity theorem to generalize the fundamental monadicity result of Theorem 2.5 to the double categorical setting.

### Construction 4.6

We construct the needed generalization of the squaring 2-monad on  to the double categorical setting. We use the recipe given by Theorem 4.3.

The underlying 2-functor of the squaring 2-monad  preserves pullbacks. Indeed taking exponentials to the walking arrow → in  gives a right adjoint, that then preserves limits. By Theorem 4.3, then  induces a 2-monad
(Note that in the notation of Theorem 4.3 this would be called $(-)^{\rightarrow }_{{{\,\textrm{opl}\,}}}$, but for reasons that will become clear in Remark 4.7 we choose this new notation.) But notice that  precisely gives the 2-category  of pseudodouble categories, normal (i.e., unitary) oplax double functors and horizontal natural transformations (i.e., with arrow components). So we get a 2-monad
which restricts to a 2-monad $(-)^{\rightarrow }_\tau$ on the 2-category  of pseudo double categories, normal pseudo double functors and horizontal natural transformations. By Theorem 4.3, the 2-monad $(-)^{\rightarrow }_\lambda$ sends a pseudo double category $\mathbb {D}$ to the pseudo double category $\mathbb {D}^\rightarrow$ given by

**Figure:**
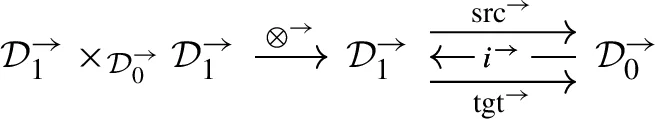

with associators induced from $\mathbb {D}$. Equivalently, $\mathbb {D}^\rightarrow$ is given by the exponential of $\mathbb {D}$ to the walking horizontal arrow → (i.e., the double category which has a single non-trivial horizontal arrow and only has vertical identities both as proarrows and double cells). See [14, Page 187] for the explicit construction of the exponential as the pseudo double category of (normal lax) double functors, horizontal transformations as arrows, (strong) vertical transformations as proarrows and (strong) modifications as double cells. Explicitly, $\mathbb {D}^{\rightarrow }$ has the following description:

- an object is a (horizontal) arrow f:A→B in $\mathbb {D}$;
- an arrow f→g is a pair (*u*, *v*) of arrows in $\mathbb {D}$ forming a commutative square in $\mathcal {D}_0$

**Figure:**
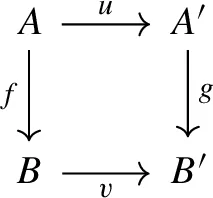
- a proarrow  is a double cell in $\mathbb {D}$

**Figure:**
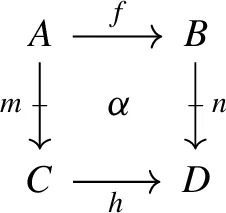
- a double cell

**Figure:**
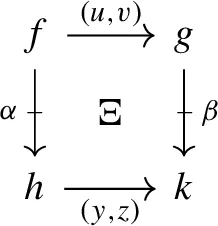

is a pair $\Xi =(\sigma ,\tau )$ making the following cube commute (as a square in $\mathcal {D}_1$)

**Figure:**
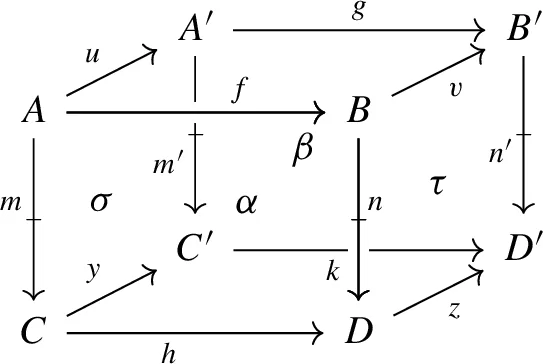

i.e., $$(\alpha |\tau )=(\sigma |\beta )$$.
- identities and composition are induced from $\mathbb {D}$.

The functor $(-)^{\rightarrow }_\lambda$ then acts on morphisms and 2-cells in the following way:

**Figure:**
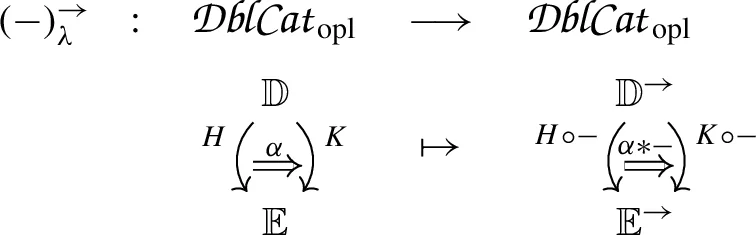

### Remark 4.7

We can merge the two 2-monads  and  into an $\mathcal {F}$-categorical monad.

Recall from [25] that $\mathcal {F}$-category theory coincides with category theory enriched over $\mathcal {F}$, which is the cartesian closed full subcategory of  determined by the functors which are injective on objects and fully faithful (i.e., full embeddings). An $\mathcal {F}$-category  is given by a collection of objects, a hom-category  of tight morphisms and a second hom-category  of loose morphisms that give 2-category structures (respectively)  and  to , together with an identity on objects, faithful and locally fully faithful 2-functor ; this is equivalent to a 2-category  with a selected subclass of morphisms called tight forming a sub-2-category (with all 2-cells). An $\mathcal {F}$-functor  is a 2-functor  that restricts to a 2-functor  (forming a commutative square); this is equivalent to $F_\lambda$ preserving tightness. And an $\mathcal {F}$-natural transformation is a 2-natural transformation $\alpha _\lambda$ between the loose parts that restricts to one between the tight parts; this is equivalent to $\alpha _\lambda$ having tight components.

$\mathcal {F}$-categories, $\mathcal {F}$-functors and $\mathcal {F}$-natural transformations then form a 2-category . An $\mathcal {F}$-monad is a pseudo-monoid in , which is equivalent to a 2-monad on the loose part whose underlying 2-functor preserves tightness and such that the multiplication and the unit have tight components.

Notice that an $\mathcal {F}$-monad *T* on  always induces two 2-monads $T_\tau$ and $T_\lambda$ on  and  respectively, together with a 2-monad morphism . As a consequence, it also induces a 2-functor (of inclusion)  (and an analogous one with lax morphisms of pseudo-algebras), by [27, Lemma 3.1].

### Proposition 4.8

The two squaring 2-monads of Construction 4.6 form an $\mathcal {F}$-monad $(-)^{\rightarrow }$ on the $\mathcal {F}$-category  of pseudo double categories, normal pseudo double functors as tight morphisms, normal oplax double functors as loose morphisms and horizontal natural transformations as 2-cells.

### Proof

By the proof of Theorem 4.3, the 2-monad $(-)^{\rightarrow }_\lambda$ on the loose part  of  restricts to the 2-functor $(-)^{\rightarrow }_\tau$. Moreover the multiplication and the unit of $(-)^{\rightarrow }_\lambda$ have tight components. Indeed, their components are actually internal strict functors. □

We can now deduce the main theorem of this section, which is a monadicity result for both pseudo and lax DOFS, generalizing the fundamental result of Theorem 2.5 to the double categorical setting. This theorem further justifies the internal definition of (lax/pseudo) double orthogonal factorization system we presented in Sect. 3.

### Theorem 4.9

Normal pseudo-algebras for the 2-monad $(-)^{\rightarrow }_\tau$ on  are precisely the pseudo double orthogonal factorization systems.

Normal pseudo-algebras for the 2-monad $(-)^{\rightarrow }_\lambda$ on  are precisely the lax double orthogonal factorization systems.

### Proof

By Theorem 4.3 and Construction 4.6, the 2-monad  is such that
But by Theorem 2.5 and Notation 2.10 (thanks to Proposition 2.12),  precisely coincides with . And then, by Definition 3.15,  is precisely the 2-category of lax double orthogonal factorization systems. We have thus proved that normal pseudo-algebras for the 2-monad $(-)^{\rightarrow }_\lambda$ give precisely lax DOFS. The restricted isomorphism of 2-categories
then shows that normal pseudo-algebras for the 2-monad $(-)^{\rightarrow }_\tau$ give precisely pseudo DOFSs. □

### Remark 4.10

The results in this section extend a body of a type of interplay results for algebras and models of finite limit sketches, with or without enhancement, as well as taking models of one sketch in those of another. In [28], Janelidze shows that internal categories in groups are equivalent to internal groups in categories; in [29], Lack and Paoli establish that for certain finite limit sketches an operad *T* on a symmetric monoidal closed category $$\mathcal {V}$$ induces an operad $T^{\prime }$ on the symmetric monoidal closed category of models of the finite limit sketch and the category of algebras for the operad $T^{\prime }$ is equivalent to the category of models of the finite limit sketch in the algebras for *T*.

### Remark 4.11

By Theorem 4.9, we can rewrite the axioms of a lax DOFS as the axioms of a normal pseudo-algebra for the 2-monad $(-)^{\rightarrow }_\lambda$ on . The latter is given by a double category $\mathbb {D}$ equipped with an algebra structure $Q:\mathbb {D}^\rightarrow \rightarrow \mathbb {D}$. *Q* has components $Q_0:\mathcal {D}_0^\rightarrow \rightarrow \mathcal {D}_0$ and $Q_1:\mathcal {D}_1^\rightarrow \rightarrow \mathcal {D}_1$, expressing the orthogonal factorization systems on $\mathcal {D}_0$ and $\mathcal {D}_1$ (i.e., on horizontal arrows and double cells viewed in the horizontal direction), by Theorem 2.5 and Remark 2.6. Interestingly, the axioms of compatibility between the two orthogonal factorization systems on $\mathcal {D}_0$ and $\mathcal {D}_1$ precisely translate into making *Q* a normal oplax functor. For example, the axiom of the oplax functor *Q* respecting sources

**Figure:**
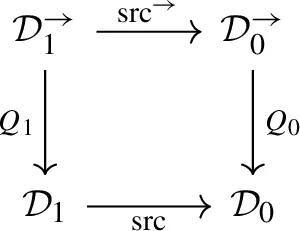

captures the axiom of $\operatorname {src}$ being a strict morphism of orthogonal factorization systems.

Moreover, by essentially the same line of reasoning as in Theorem 4.9, we can describe a lax morphism between lax DOFSs as a lax morphism between normal pseudo-algebras for the 2-monad $(-)^{\rightarrow }_\lambda$ on . The latter, for a pseudo-morphism between normal pseudo-algebras $Q^\mathbb {D}:\mathbb {D}^\rightarrow _\lambda \rightarrow \mathbb {D}$ and $Q^\mathbb {E}:\mathbb {E}^\rightarrow _\lambda \rightarrow \mathbb {E}$, is given by an oplax double functor $F :\mathbb {D}\rightarrow \mathbb {E}$ equipped with a transformation ϝ as in the following diagram:

**Figure:**
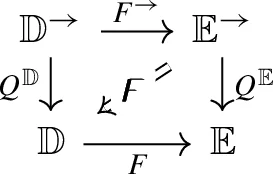

that commutes appropriately with the data of $Q^\mathbb {D}$ and $Q^\mathbb {E}$. In particular, by Proposition 2.12, the underlying natural transformations

$$\begin{aligned} \digamma _i : Q^\mathbb {E}_i \circ F^\rightarrow _i \Rightarrow F_i \circ Q_i^\mathbb {D}\end{aligned}$$

for i=0,1, equivalently witness that $F_i$ preserves the *right class* of morphisms in the factorization system on $\mathcal {D}_i$ given by $Q_i^\mathbb {D}$. Furthermore, 2-cells between such morphisms can be given by arrow-direction 2-cells that give rise to commuting cylinders,

**Figure:**
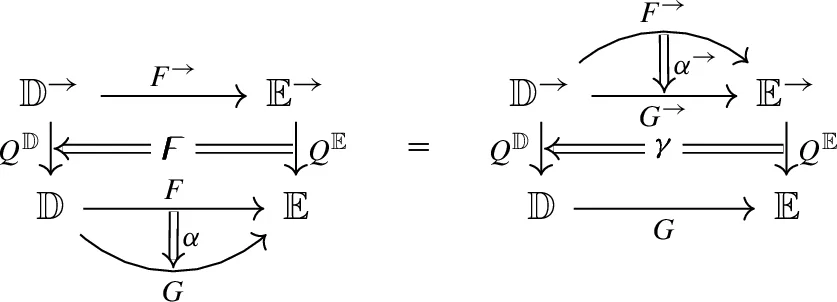

i.e., pairs of compatible 2-cells between lax morphisms of categories with orthogonal factorization systems. So we can conclude:

### Proposition 4.12

A lax morphism between lax DOFSs is an oplax double functor that preserves the right class – both of horizontal morphisms, and of double cells. Similarly, a pseudo-morphism between DOFSs is a pseudo double functor that preserves both left and right classes of horizontal arrows and double cells. 2-Cells between these morphisms correspond to arrow direction (horizontal) transformations between the double functors that are compatible with the algebra structure on both the arrows and the double cells.

### Remark 4.13

The inclusion of algebras  described in Remark 4.7, guaranteed from having an $\mathcal {F}$-monad $(-)^{\rightarrow }$ on , is the inclusion of pseudo DOFSs into lax DOFSs.

### Remark 4.14

The whole theory of this section can be dualized for colax DOFSs. This way we obtain that normal pseudo-algebras for the 2-monad $(-)^\rightarrow _{{{\,\textrm{lax}\,}}}$ on  are precisely colax double orthogonal factorization systems. A colax morphism between colax DOFSs is a lax double functor that preserves the left class – both of horizontal morphisms, and of double cells.

### Remark 4.15

We see here that the treatment of double orthogonal factorization systems as algebras for a monad gets us various 2-categories of double orthogonal factorization systems on double categories (one for each flavour: oplax, lax, pseudo, strict, where obviously the last two are sub-2-categories of the former). However, keeping the algebra functors within the larger context of all double categories with various types of double functors gives us further options for morphisms and 2-cells between them. In [30], Grandis and Paré introduce a double category of double categories with lax functors in one direction and oplax functors in the other direction and transformations in the arrow direction as double cells. The double cells of this double category can be used to define a notion of morphism between double categories with a lax orthogonal factorization systems that preserves the left class (whereas the vertical composition preserves the right class). In this paper we will not explore this further.

Another type of morphism that is afforded us by this approach is to go from a double category with a lax DOFS to one with an oplax DOFS (or vice versa). In this case the morphism *F* needs to be a pseudo-functor. We will have use for these morphisms in Sect.5 when we want to describe the category of DOFSs that extend a common OFS on the category of arrows and objects of the double category.

### Remark 4.16

Double categories also give us the option to give the hom-categories of the 2-category of double categories with a lax DOFS the structure of double categories. We do this by adding vertical transformations to the mix; i.e., transformations in the proarrow direction. Let $\mathbb {D}$ and $\mathbb {E}$ be double categories with a lax DOFS and $F,G:\mathbb {D}\rightarrow \mathbb {E}$ be oplax double functors which also preserve the right classes. A vertical transformation $\theta :F\Rightarrow \negthinspace \negthinspace \negthinspace \negthinspace \negthinspace \negthinspace |\,\,\,\, G$ would correspond to a functor $\theta :\mathcal {D}_0\rightarrow \mathcal {E}_1$ satisfying the usual properties. In this case, we only need to add the requirement that θ be a lax morphism of algebras; i.e., that it preserves the right class. We can now get the double category structure on the homs by taking the full sub double category on these horizontal and vertical transformations: simply, take all modifications that fit.

There is even more possible: we can combine all the different types of DOFS in one 2-category where we use only pseudo-functors as morphisms and the arrow direction double cells as 2-cells. We will not need this result in full. We will only use it in the next section for DOFSs extending a common OFS on the arrows and objects of a double category.

## Extending an Arrow OFS to a DOFS

In this section we present some results around the question when a given OFS on the category of objects and arrows of a double category can be extended to a DOFS and how these extensions are related to each other. We will illustrate this by adding new extensions to some examples from Sect. 3 and introducing some completely new examples.

### Theoretical Results

We present further examples of double orthogonal factorization systems. Every DOFS $(\mathbb {L},\mathbb {R})$ on $\mathbb {D}$ has an underlying OFS $(\mathcal {L}_0,\mathcal {R}_0)$ on its category of arrows and objects, $\mathcal {D}_0$. In this section we will consider the extensions of various well-known orthogonal factorization systems to double factorization systems and we will explore the morphisms between these extensions.

For our first general result we want to address the requirement that the source and target structure arrows, $\operatorname {src}$ and $\operatorname {tgt}$ of $\mathbb {D}$ need to be strict morphisms of orthogonal factorization systems. We give a condition under which a pair of orthogonal factorization systems for which these structure arrows only preserve the left and the right classes can be adjusted to a pair for which these maps are strict; i.e., preserve the chosen factorizations. We give in fact a slightly stronger result.

#### Proposition 5.1

Let $\mathbb {D}$ be a double category such that both $\mathcal {D}_0$ and $\mathcal {D}_1$ are categories with an OFS and chosen factorizations such that the source, target and units functors preserve the left and right classes, but not necessarily the chosen factorizations. Then the chosen factorizations of the cells in $\mathbb {D}$ can be adjusted to obtain an OFS for $\mathcal {D}_1$ such that the source, target and units functors are strict morphisms of OFSs, if for every diagram of invertible arrows and a proarrow as on the left of the following diagram there is a horizontally invertible double cell θ as on the right:

**Figure:**
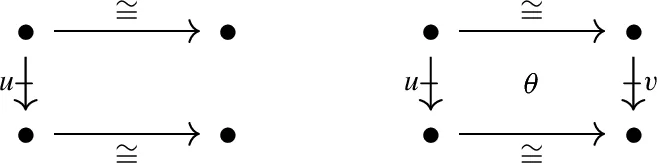

#### Proof

Given a choice of factorizations for the arrows, $f=r_f\circ \ell _f$ where $\ell _f\in \mathcal {L}$ and $r_f\in \mathcal {R}$, we will show how to adjust the factorization for the double cells so that all of $\operatorname {src}$, $\operatorname {tgt}$ and *i* preserve the chosen factorizations.

We make the units functor strict by choosing the corresponding factorizations for the unit cells: $\ell _{i_f}=i_{\ell _f}$ and $r_{i_f}=i_{r_f}$.

To adjust the factorizations of the other cells so that source and target become strict, let

**Figure:**
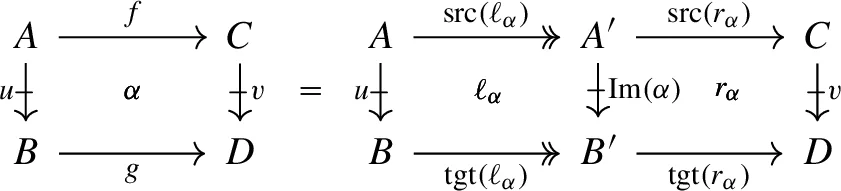

be the chosen factorization for a cell α. By orthogonality of the factorization system on $D_0$, there are unique isomorphisms $d_1$ and $d_2$ making the following diagrams commute:

**Figure:**
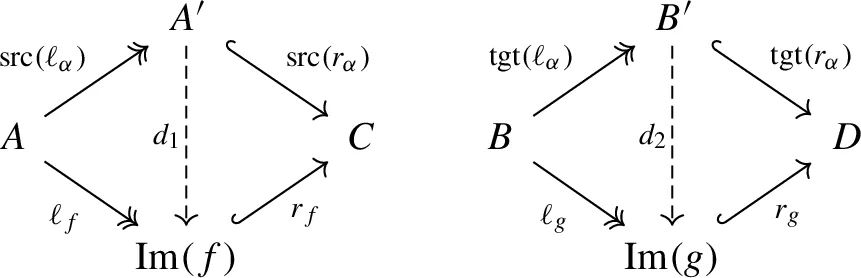

Now choose a horizontally invertible cell θ according to the condition of the proposition:

**Figure:**
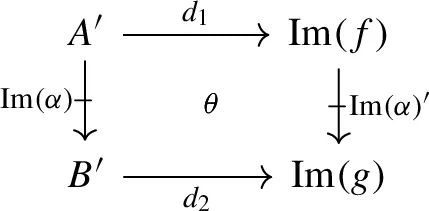

Now let $\ell '_\alpha =\theta \circ \ell _\alpha$ and $r'_\alpha =r_\alpha \circ \theta ^{-1}$, where $\theta ^{-1}$ is the horizontal inverse of θ. Since θ is horizontally invertible, $\ell '_\alpha$ is in the left class on $\mathcal {D}_1$ and $r'_\alpha$ is in the right class on $\mathcal {D}_1$ and with this new choice of factorizations, the source and target functors have become strict. □

#### Corollary 5.2

Given an internal pseudo-category in  (resp. , ) where the source, target and unit functors are pseudo-morphisms of OFSs, but not strict. If the underlying double category satisfies the condition of Proposition 5.1, then the chosen factorizations of the cells can be adjusted to make this a lax (resp. colax, pseudo) DOFS.

#### Remark 5.3

Note that since we do not change the left and right classes of the OFS on the category of cells and proarrows in Proposition 5.1 and Corollary 5.2 in order to make it part of the DOFS, the new OFS on the category of double cells and proarrows is isomorpic to the original one by a pseudo-morphism of OFSs. In particular, given an OFS on the category of arrows and objects of a double category, any family of choices of horizontally-invertible double cells as in this proposition gives rise to a functorial way to replace an OFS on the category of double cells and proarrows for which either the class of left cells or the class of right cells is closed under vertical composition, and that has left and right classes compatible with those on the category of arrows and objects with one that has also compatible chosen factorizations.

In the next proposition we discuss properties on a double category that assure us that there is at least one double orthogonal factorization system that extends a given factorization system on its arrows. We will make use of the notions of restriction (or, cartesian cell) and extension (or, opcartesian cell) in a double category [31]. The opcartesian cells are also called absolute right Kan extensions in [32].

#### Definition 5.4

1. A double category has an *extension*, or *opcartesian cell*, for the diagram on the left below if there is a cell $\gamma _{s,s'}^M$ as on the right,

**Figure:**
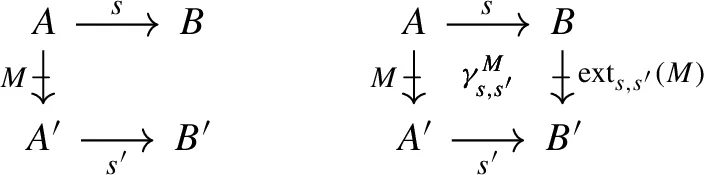

with the following universal property: for each double cell

**Figure:**
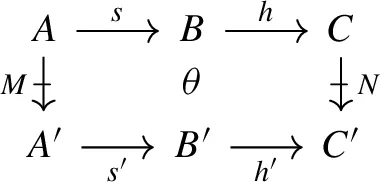

there is a unique cell $\theta^{\prime }$ such that

**Figure:**
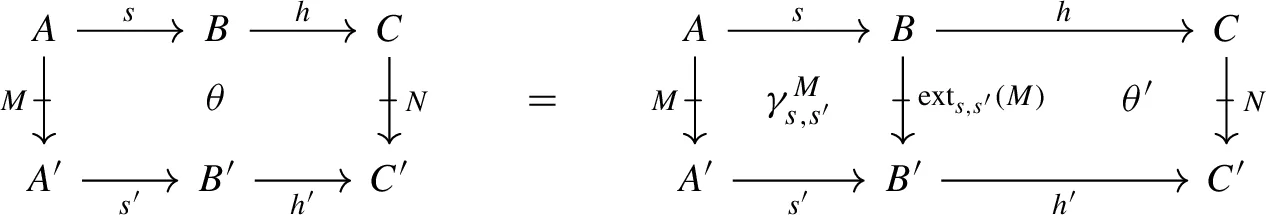
2. The definition of *restriction*, or *cartesian cell*, is dual to that of extension.
3. We say that a double category has all extensions along arrows in a class $\mathcal {S}$ if it has all extensions for diagrams where the arrows are in $\mathcal {S}$.
4. Dually, a double category has all restrictions along arrows in a class $\mathcal {S}$ if it has all restrictions for diagrams where the arrows are in $\mathcal {S}$.

#### Proposition 5.5

Let $\mathbb {D}$ be a double category with an orthogonal factorization system $(\mathcal {L},\mathcal {R})$ on $\mathcal {D}_0$.

1. If $\mathbb {D}$ has all extensions along the arrows in $\mathcal {L}$, it has a lax DOFS $(\mathbb {L},\mathbb {R})=(\mathcal {L},\mathcal {R})_{\operatorname {extn}}$ with $(\mathcal {L}_0,\mathcal {R}_0)=(\mathcal {L},\mathcal {R})$, where $\mathcal {L}_1$ consists of all extension cells with horizontal boundaries in $\mathcal {L}$, and $\mathcal {R}_1$ consists of all double cells with horizontal boundaries in $\mathcal {R}$;
2. If $\mathbb {D}$ has all restrictions along arrows in $\mathcal {R}$, it has a colax DOFS $(\mathbb {L},\mathbb {R})=(\mathcal {L},\mathcal {R})_{\operatorname {restr}}$ with $(\mathcal {L}_0,\mathcal {R}_0)=(\mathcal {L},\mathcal {R})$, where $\mathcal {L}_1$ has all double cells with horizontal boundaries in $\mathcal {L}$, and $\mathcal {R}_1$ has all restriction cells with horizontal boundaries in $\mathcal {R}$.

#### Proof

1. It is clear that both the class of extension cells with $$\mathcal {L}$$-boundaries and the class of cells with $$\mathcal {R}$$-boundaries are closed under horizontal composition and contain all horizontally invertible cells. It is also clear that the right class $$\mathcal {R}_1$$ is closed under vertical composition. To factor an arbitrary double cell, factor its horizontal boundary arrows in $$(\mathcal {L},\mathcal {R})$$, and take the extension of its vertical source arrow *M* along the arrows in $$\mathcal {L}$$. Then the right side of the factorization is the cell $\theta^{\prime }$ that exists by the universal property of the extension cell.

**Figure:**
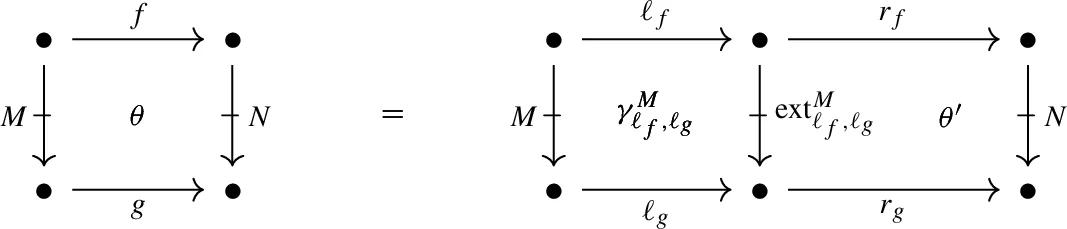

Finally, to prove the lifting property, we will denote a double cell θ as in the previous diagram by $M\xrightarrow []{(f,\theta ,g)}N$. With this notation, we are considering a commutative square of the form,

**Figure:**
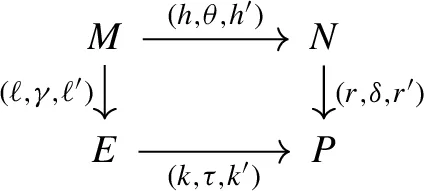

where γ is an extension cell, $\ell ,\ell '\in \mathcal {L}$ and $r,r'\in \mathcal {R}$. This gives us the following two diagrams in the arrow category of $\mathcal {D}_0$,

**Figure:**
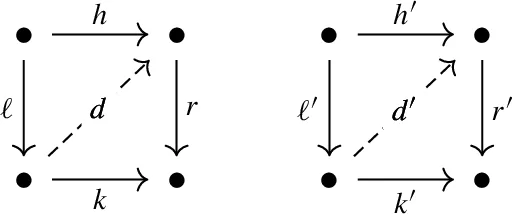

which have unique diagonals *d* and $d^{\prime }$ because $$(\mathcal {L},\mathcal {R})$$ is an OFS. Hence, $$h=d\circ \ell $$ and $$h'=d'\circ \ell '$$. The universal property of γ as extension cell gives rise to a unique double cell $\theta^{\prime }$ as in the following diagram,

**Figure:**
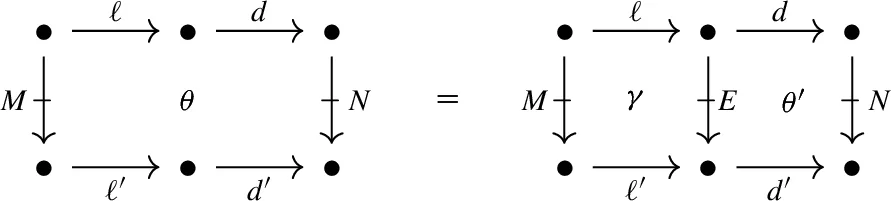

This gives the commutativity of the upper triangle in the following diagram,

**Figure:**
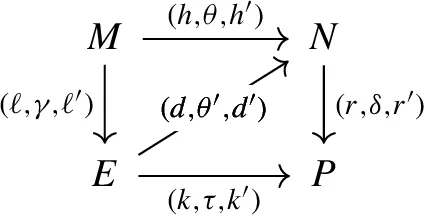

For the lower triangle, note that
  $$\begin{aligned} ((r,\delta ,r')\circ (d,\theta ',d')) \circ (\ell ,\gamma ,\ell ')= &   (r,\delta ,r')\circ ((d,\theta ',d')\circ (\ell ,\gamma ,\ell '))\\= &   (r,\delta ,r')\circ (h\theta ,h')\\= &   (k,\tau ,k')\circ (\ell ,\gamma ,\ell ') \end{aligned}$$
  The uniqueness in the universal property of γ now gives us that $$(r,\delta ,r')\circ (d,\theta ',d')=(k,\tau ,k')$$. So $\left(d,\theta^{\prime },d^{\prime }\right)$ is the required lifting. Its uniqueness follows again from the universal property of γ. Finally, a choice of an extension cell for each diagram,

**Figure:**
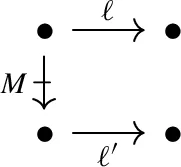

gives rise to a choice of factorizations of the double cells such that their sources and targets are the chosen factorizations on the arrows. So we have a lax DOFS.
2. This follows from the first part by duality.

□

#### Example 5.6

If a double category has all extensions (and hence all restrictions), the (all morphisms, isos) factorization system on the arrows can be extended to a DOFS with the (opcartesian, pseudo-globular) factorization system on the cells (any chosen factorization of the arrows can be extended to the cells by Proposition 5.1). In the same way, the (isos, all morphisms) factorisation systems can be extended to the (cartesian, pseudo-globular) DOFS. A cell is pseudo-globular if its source and target arrows are isomorphisms.

Observe that each DOFS on $\mathbb {D}$ gives rise to a suitable (i.e., normal oplax, normal lax, pseudo or strict, depending on the type of factorization system) double functor from $\mathbb {D}^{\rightarrow }$ to $\mathbb {D}$. Now a morphism between two double orthogonal factorization systems on the same double category with the same factorization system on the arrow category corresponds to a diagram

**Figure:**
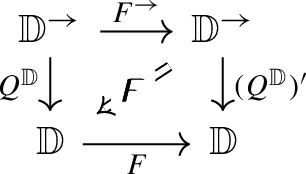

where $F^\rightarrow$ is the identity on $\mathbb {D}^\rightarrow$. Hence, *F* is the identity on $\mathbb {D}$. So a morphism between such double orthogonal factorization systems is a horizontal identity on objects transformation between the double functors representing the two factorization systems. Note that [30] gives a detailed description of horizontal transformations, where the domain and the codomain may be double functors with distinct types of laxity.

#### Theorem 5.7

1. Let $\mathbb {D}$ be a double category with an OFS $(\mathcal {L}_0,\mathcal {R}_0)$ on its category of arrows and all extensions along the left class $\mathcal {L}_0$. Then for any type of DOFS $(\mathbb {L},\mathbb {R})$ on $\mathbb {D}$ with $(\mathcal {L}_0,\mathcal {R}_0)$ as the arrow part, there is a unique morphism $(\mathbb {L},\mathbb {R})\rightarrow (\mathcal {L}_0,\mathcal {R}_0)_{\operatorname {extn}}$ of DOFSs with a common arrow OFS on $\mathbb {D}$.
2. Let $\mathbb {D}$ be a double category with an OFS $(\mathcal {L}_0,\mathcal {R}_0)$ on its category of arrows and all restrictions along the right class $\mathcal {R}_0$. Then for any type of DOFS $(\mathbb {L},\mathbb {R})$ on $\mathbb {D}$ with $(\mathcal {L}_0,\mathcal {R}_0)$ as the arrow part, there is a unique morphism $(\mathcal {L}_0,\mathcal {R}_0)_{\operatorname {restr}}\rightarrow (\mathbb {L},\mathbb {R})$ of DOFSs with a common arrow OFS on $\mathbb {D}$.

#### Proof

1. Let $(\mathbb {L},\mathbb {R})$ be a lax (resp. colax, pseudo, strict) DOFS on $\mathbb {D}$, with corresponding oplax (resp. lax, pseudo, strict) double functor $Q:\mathbb {D}^\rightarrow \rightarrow \mathbb {D}$. For a double cell θ in $\mathbb {D}$ we have now the following two double cell factorizations that share the same factorizations of the boundary horizontal arrows:

**Figure:**
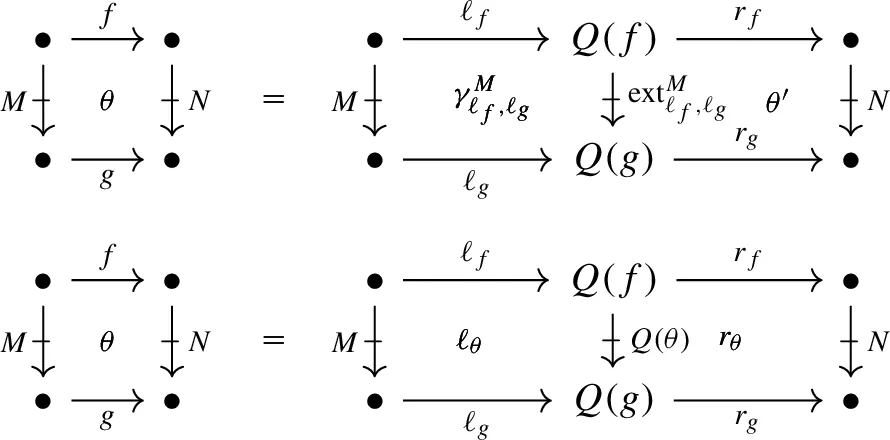

By the universal property of the extension cell $\gamma ^M_{\ell _f,\ell _g}$ there is a unique globular double cell

**Figure:**
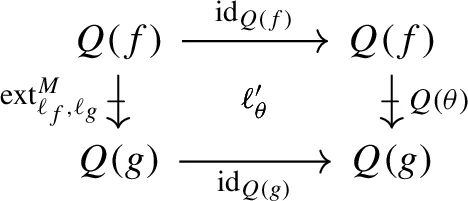

Let $Q_{\operatorname {extn}}:\mathbb {D}^\rightarrow \rightarrow \mathbb {D}$ be the double functor representing the extension DOFS. Then we define the components of a horizontal transformation

**Figure:**
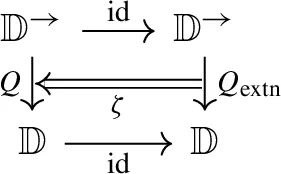

by $\zeta _f=\operatorname {id}_{Q(f)}$ for all objects *f* of $\mathbb {D}^{\rightarrow }$ (i.e., arrows of $\mathbb {D}$) and $\zeta _\theta =\ell '_\theta$ for each proarrow θ of $\mathbb {D}^\rightarrow$. Using the universal property of the extension cells it is easy to check that this family does indeed define a horizontal transformation, and hence a pseudo-morphism of DOFS on $\mathbb {D}$, $(\mathbb {L},\mathbb {R})\rightarrow (\mathcal {L}_0,\mathcal {R}_0)_{\operatorname {extn}}$. For each component of such a pseudo-morphism there is only one cell that could fulfill the role, so this pseudo-morphism is unique.
2. This follows from the first part by duality.

□

Recall that a fibrant double category [33], also called a framed bicategory [31], is a double category with all extensions and restrictions.

#### Corollary 5.8

For any fibrant double category $\mathbb {D}$ with an OFS $(\mathcal {L},\mathcal {R})$ on its category of arrows, the DOFS $(\mathcal {L},\mathcal {R})_{\operatorname {restr}}$ is initial and $(\mathcal {L},\mathcal {R})_{\operatorname {extn}}$ is terminal among the DOFSs of all types on $\mathbb {D}$ with $(\mathcal {L},\mathcal {R})$ as arrow part.

### Examples

We will now present examples of double categories with multiple DOFS that share the same OFS on the category of objects and arrows of the double category.

#### Example 5.9

*(The double category* $\mathbb {M}\textrm{od}$.*)* In the category $\mathcal {R}\text{ ing }$ of associative unital rings, the class $\mathcal {L}_{\operatorname {surj}}$ of surjective morphisms together with the class $\mathcal {R}_{\operatorname {inj}}$ of injective morphisms form an orthogonal factorization system. This follows from the fact that surjective and injective functions form an orthogonal factorization system in the category of sets, and the image of a ring morphism is itself a ring. Moreover, the bijection between two distinct factorizations into a surjective and an injective morphism is itself a ring isomorphism. The OFS $(\mathcal {L}_{\operatorname {surj}},\mathcal {R}_{\operatorname {inj}})$ of $\mathcal {R}\text{ ing }$ appears as the factorization system for the horizontal arrows in a colax DOFS $(\mathbb {L}_{\operatorname {surj}},\mathbb {R}_{\operatorname {inj}})$ on the double category $\mathbb {M}\textrm{od}$ of associative unital rings, bimodules, and equivariant morphisms. The left class $(\mathcal {L}_{\operatorname {surj}})_1$ of the factorization system on the double cells consists of surjective cells whose horizontal morphisms are surjective, while the right class $(\mathcal {R}_{\operatorname {inj}})_1$ consists of injective cells whose horizontal morphisms are injective. Every cell φ in $\mathbb {M}\textrm{od}$ admits a factorization of the form:

**Figure:**
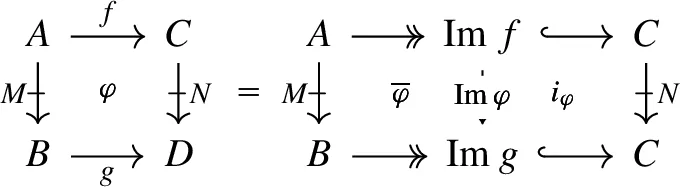

where $\bar{\varphi }$ denotes the restriction of φ to its image, and $i_\varphi$ is the inclusion cell. Furthermore, $(\mathcal {L}_{\operatorname {surj}})_1$ is closed under vertical composition, since the tensor product of surjective morphisms between modules is again surjective. However, the right class $(\mathcal {R}_{\operatorname {inj}})_1$ is not closed under vertical composition. For instance, the vertical composition of the cell

**Figure:**
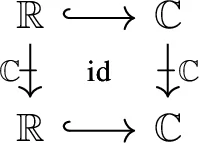

with itself does not belong to the right class $(\mathcal {R}_{\operatorname {inj}})_1$. This is because $i\otimes _\mathbb {R} 1 \ne 1\otimes _\mathbb {R}i$, while over $\mathbb {C}$ one has $i\otimes _\mathbb {C}1 = 1\otimes _\mathbb {C} i$. (Here, the left vertices labeled $\mathbb {R}$ in the cell $\textrm{id}$, as well as the subscript in the tensor operator, refer to the field of real numbers.)

This is not the only DOFS on $\mathbb {M}\textrm{od}$, because the double category $\mathbb {M}\textrm{od}$ is fibrant [31]. The restriction and extension cells are of the form

**Figure:**
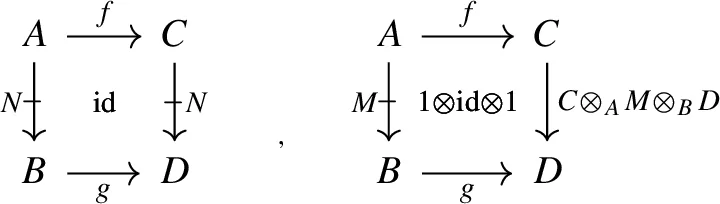

respectively. Here the left proarrow *N* in the restriction double cell is the $A\text {-}B$-bimodule induced by *f* and *g*, and the right proarrow $C\otimes _A M\otimes _B D$ in the extension is the $C\text {-}D$-bimodule obtained by tensoring the $C\text {-}A$-bimodule *C*, the $A\text {-}B$-bimodule *M*, and the $B\text {-}D$-bimodule *D*. The morphism $1\otimes \text{ id }\otimes 1$ maps an element m∈M to $1\otimes m\otimes 1$.

By Proposition 5.5, there exist a DOFS $(\mathcal {L}_{\operatorname {surj}}, \mathcal {R}_{\operatorname {inj}})_\text {restr}$ and a DOFS $(\mathcal {L}_{\operatorname {surj}},\mathcal {R}_{\operatorname {inj}})_\text {extn}$ on $\mathbb {M}\textrm{od}$, induced by restrictions and extensions cells. In these DOFSs, every cell φ admits factorizations of the form:

**Figure:**
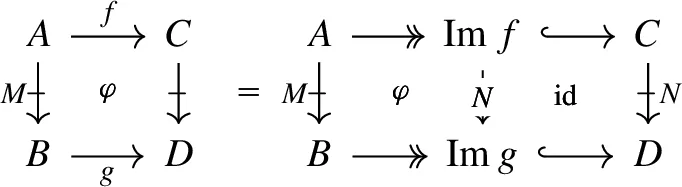

and

**Figure:**
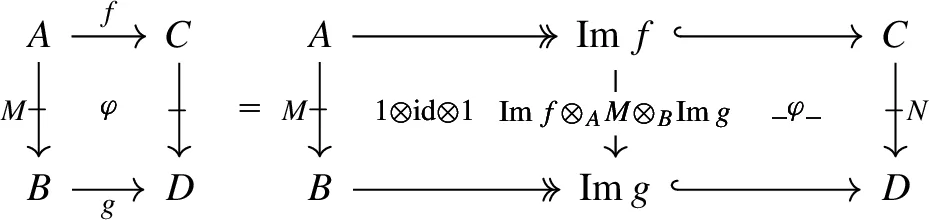

respectively, where the cell $\_\varphi \_$ sends an element $f(a)\otimes _A m\otimes _B g(b)$ to f(a)φ(m)g(b).

The DOFS $(\mathcal {L}_{\operatorname {surj}},\mathcal {R}_{\operatorname {inj}})_\text {restr}$ and $(\mathcal {L}_{\operatorname {surj}},\mathcal {R}_{\operatorname {inj}})_\text {extn}$ differ from the DOFS $(\mathbb {L}_{\operatorname {surj}},\mathbb {R}_{\operatorname {inj}})$. By Theorem 5.7, there exist pseudo-morphisms of DOFSs, $(\mathbb {L}_{\operatorname {surj}},\mathbb {R}_{\operatorname {inj}})\rightarrow (\mathcal {L}_{\operatorname {surj}},\mathcal {R}_{\operatorname {inj}})_\text {restr}$ and $(\mathcal {L}_{\operatorname {surj}},\mathcal {R}_{\operatorname {inj}})_\text {extn}\rightarrow (\mathbb {L}_{\operatorname {surj}},\mathbb {R}_{\operatorname {inj}})$. Explicitly, the globular double cells that define these pseudo-morphisms are

**Figure:**
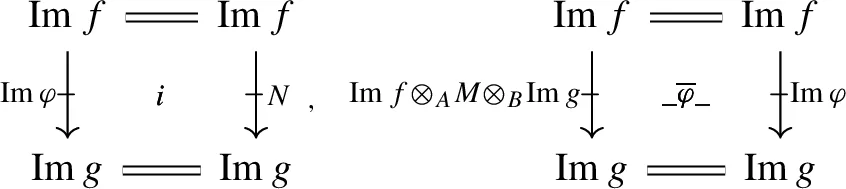

respectively, where the cell $\_\overline{\varphi }\_$ maps an element $f(a)\otimes _A m\otimes _B g(b)$ to $f(a)\overline{\varphi }(m)g(b)$.

Our next example will be the double category $\mathbb {P}\textrm{rof}$ of small categories, profunctors and functors. This double category is also known to be fibrant, so any orthogonal factorization system on  will give rise to one or more DOFS on $\mathbb {P}\textrm{rof}$. Here we want to consider the OFS on  by strong epimorphisms and monomorphisms. We beging by unpacking this factorization system in greater detail.

#### Observation 5.10

In , any functor $F:\mathcal {A}\rightarrow \mathcal {B}$ admits the following factorization. We write $\mathcal {I}m(F)$ for the subcategory of $\mathcal {B}$ on the objects of the form *F*(*A*) with *A* in $\mathcal {A}$ and arrows generated under composition in $\mathcal {B}$ by the arrows of the form *F*(*f*) with *f* in $\mathcal {A}$. We then factor *F* as

**Figure:**
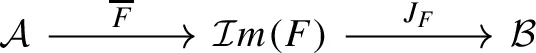

where $\overline{F}$ is the restriction of *F* and $J_F$ is the inclusion of the subcategory into $\mathcal {B}$.

#### Definition 5.11

A functor $F:\mathcal {A}\rightarrow \mathcal {B}$ is *compositionally surjective* if $\mathcal {B} = \mathcal {I}m(F)$.

#### Observation 5.12

The class $\mathcal {L}$ of compositionally surjective functors and the class $\mathcal {R}$ of injective-on-objects faithful functors form an orthogonal factorization system in . Moreover, injective-on-objects faithful functors are precisely the monomorphisms in . Hence, compositionally surjective functors are precisely the strong epimorphisms in .

We will use this factorization system in the next example.

#### Example 5.13

The OFS $(\mathcal {L}_{\operatorname {st.epi}},\mathcal {R}_{\operatorname {mono}})$ on  given in the above observation and the surjective-injective OFS on  induce the DOFS $(\mathbb {L}_{\operatorname {epi}},\mathbb {R}_{\operatorname {mono}})$ on the double category of profunctors $\mathbb {P}\textrm{rof}$. The left class $(\mathcal {L}_{\operatorname {epi}})_1$ of double cells consists of surjective natural transformations (where every component is surjective) with compositionally surjective functors as horizontal arrows. The right class $(\mathcal {R}_{\operatorname {mono}})_1$ of double cells consists of injective natural transformations (where every component is injective) with injective-on-objects faithful functors as horizontal arrows. It is clear that both $(\mathcal {L}_{\operatorname {epi}})_1$ and $(\mathcal {R}_{\operatorname {mono}})_1$ are closed under horizontal composition and contain all horizontally invertible cells. Before showing that any cell

**Figure:**
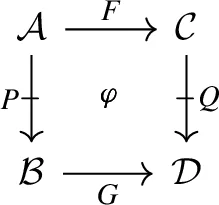

has an $((\mathcal {L}_{\operatorname {epi}})_1, (\mathcal {R}_{\operatorname {mono}})_1)$-factorization, it is necessary to define the profunctor , where $\mathcal {I}m(F)$ and $\mathcal {I}m(G)$ are defined as in Observation 5.12. For any objects $x\in \mathcal {I}m(F)$, $y\in \mathcal {I}m(G)$, with $a\in \mathcal {A}$ and $b\in \mathcal {B}$ that satisfy F(a)=x and G(b)=y, consider the surjective-injective factorization for the function $\varphi _{b,a}$:

**Figure:**
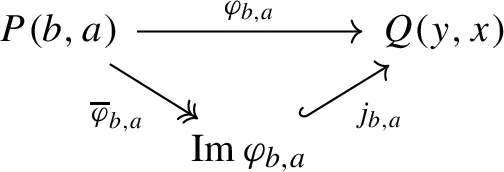

Taking the coproduct over every pair of elements in the fibers of *x* and *y*, we get the commutative diagram for each pair (*b*, *a*) in the fiber over (*y*, *x*):

**Figure:**
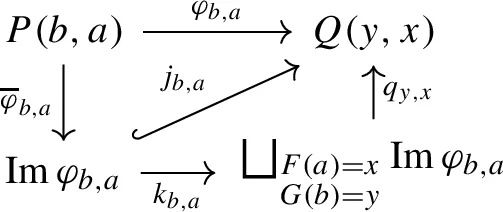

The functor $\mathcal {I}m(\varphi )$ is defined on objects as

$$\begin{aligned} \mathcal {I}m(\varphi )(y,x):= \bigsqcup _{\begin{array}{c} F(a)=x \\ G(b)=y \end{array}} \operatorname {Im}\varphi _{b,a} \end{aligned}$$

On morphisms it is defined as follows: if (*s*, *t*) is a morphism in $\mathcal {I}m(G)^{{\operatorname {op}}}\times \mathcal {I}m(F)$, then there are morphisms $s_1,\ldots ,s_m$ in $\mathcal {B}^{op}$ and $t_1,\ldots ,t_n$ in $\mathcal {A}$ such that $s = G(s_m)\cdots G(s_1)$ and $t=F(t_n)\cdots F(t_1).$ Without loss of generality, n=m (otherwise add identity arrows as needed). For every i=1,…,n there is a unique arrow $\operatorname {Im}\varphi (Gs_i,Ft_i)$ by the fact that OFSs are functorial (Definition 2.2):

**Figure:**
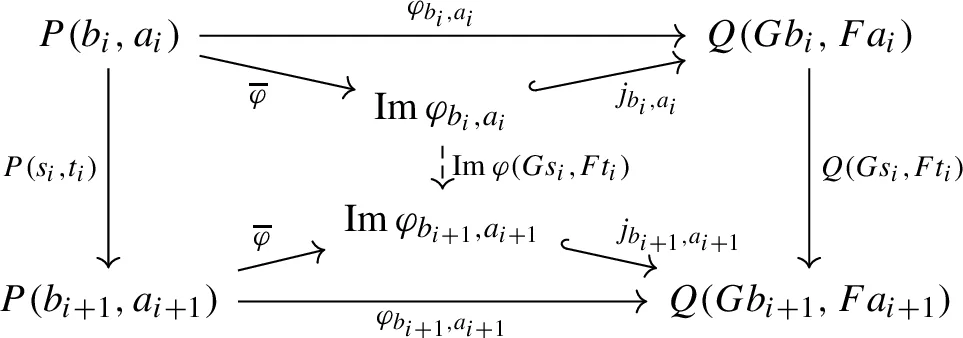

The composition $\operatorname {Im}\varphi (Gs_n,Ft_n)\cdots \operatorname {Im}\varphi (Gs_1,Ft_1)$ does not depend on the selection of the $s_i$ and the $t_i$ for i=1,…,n because $j_{b_{n+1},a_{n+1}}$ is injective (it is left cancellable) and for each choice it holds that

$$\begin{aligned}j_{b_{n+1},a_{n+1}} \operatorname {Im}\varphi (Gs_n,Ft_n)\cdots \operatorname {Im}\varphi (Gs_1,Ft_1)= &   Q(Gs_n,Ft_n)\cdots Q(Gs_1, Ft_1)j_{b_1,a_1}\\  = &   Q(s,t)j_{b_1,a_1}.\end{aligned}$$

The functor $\mathcal {I}m\varphi$ is now defined on a morphism (*s*, *t*) as

$$\begin{aligned} \mathcal {I}m(\varphi )(s,t) := \bigsqcup \operatorname {Im}\varphi (Gs_n,Ft_n)\cdots \operatorname {Im}\varphi (Gs_1,Ft_1). \end{aligned}$$

If f:a→c is a morphism in $\mathcal {A}$ and h:d→b is a morphism in $\mathcal {B}$, set $\alpha = \operatorname {Im}\varphi (Gh, Ff)$. This verifies that the following diagram commutes:

**Figure:**
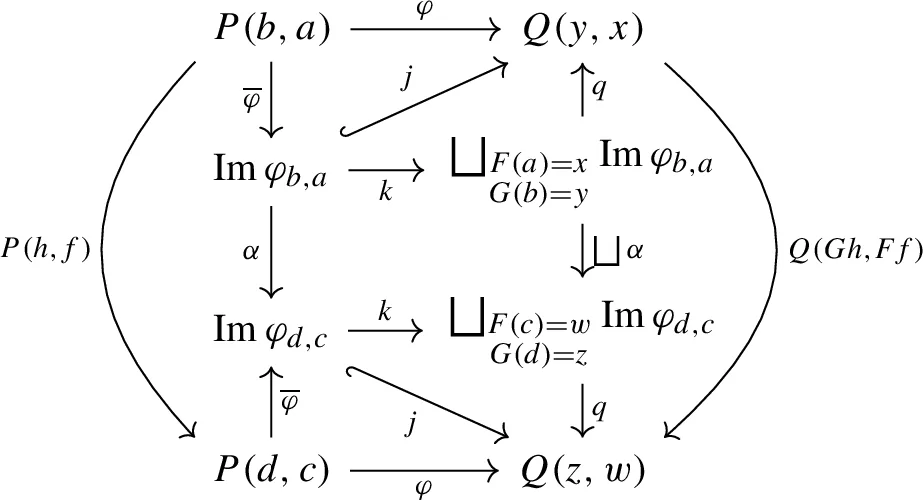

Thus, $k\overline{\varphi }$ and *q* are natural transformations and therefore the next equation holds:

**Figure:**
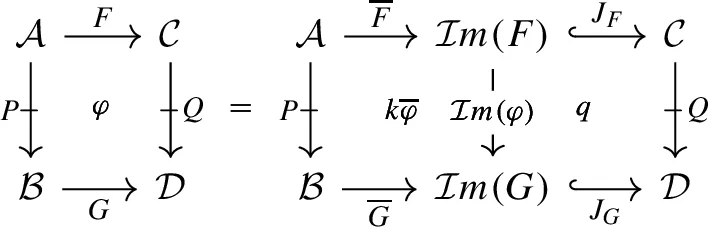

We now recall the vertical composition. Given two profunctors  and , their vertical composition $P'\otimes _{\mathcal {B}} P$ is defined by the coend

$$\begin{aligned} P'\otimes _\mathcal {B} P := \int ^{b\in \mathcal {B}}P'(e,b)\times P(b,a). \end{aligned}$$

The left class $(\mathcal {L}_{\operatorname {epi}})_1$ is closed under vertical composition because the tensor product of surjective morphisms of profunctors is again surjective. On the other hand, the counterexample in Example 5.9 shows that the right class $(\mathcal {R}_{\operatorname {mono}})_1$ is not closed under vertical composition.

The double category of profunctors is fibrant. The restriction and extension cells are of the form

**Figure:**
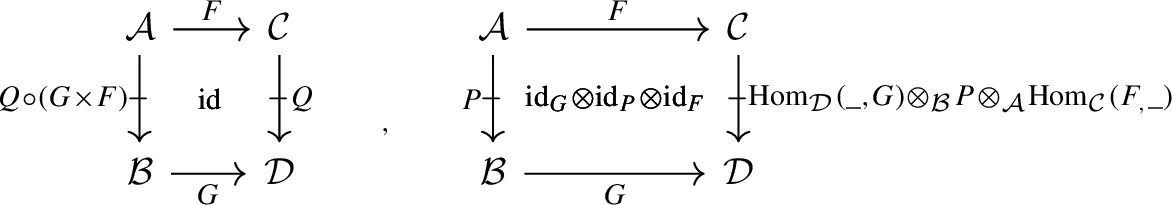

respectively, where for each pair (*b*, *a*) the natural transformation $\text {id}_G\otimes \textrm{id}_P\otimes \text {id}_F$ is the function $P(b,a) \rightarrow \text {Hom}_\mathcal {D}(Gb,G)\otimes _\mathcal {B} P\otimes _\mathcal {A}\text {Hom}(F_,Fa)$ sending x∈P(b,a) to $\text {id}_{Gb}\otimes x\otimes \text {id}_{Fa}$. Then, using the OFS in  described in Observation 5.12, Proposition 5.5 yields two DOFSs on the double category $\mathbb {P}\textrm{rof}$, namely $(\mathcal {L}_{\operatorname {st.epi}},\mathcal {R}_{\operatorname {mono}})_\text {restr}$ and $(\mathcal {L}_{\operatorname {st.epi}},\mathcal {R}_{\operatorname {mono}})_\text {extn}$. In these, each cell φ factors as

**Figure:**
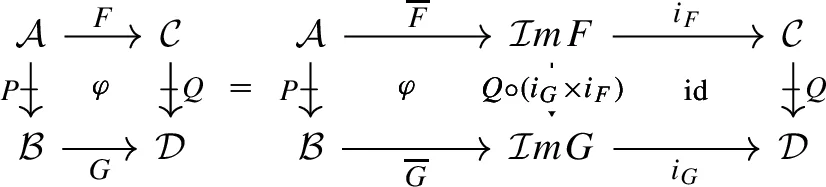

and

**Figure:**
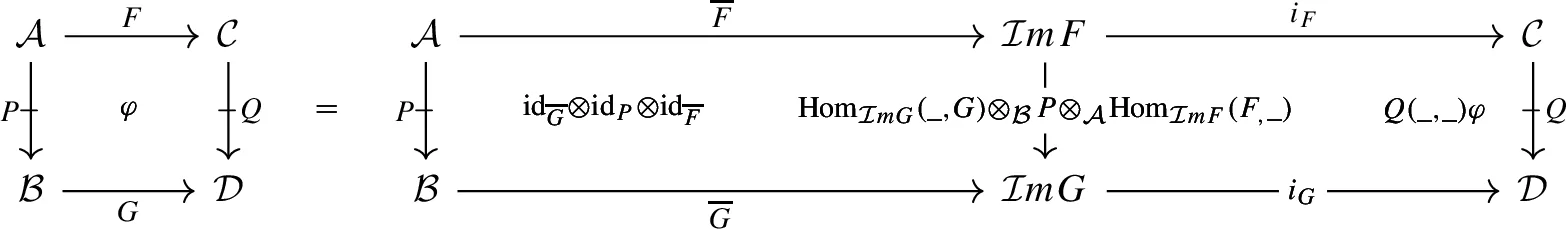

respectively. Here, the function

$$\begin{aligned} (Q(\_,\_)\varphi )_{(Gb,Fa)}:\text {Hom}(Gb,G)\otimes P \otimes \text {Hom}(F,Fa) \rightarrow Q(Gb,Fa) \end{aligned}$$

is defined on an element $g\otimes x\otimes f$ by

$$\begin{aligned} (Q(\_,\_)\varphi )_{(Gb,Fa)} (g\otimes x\otimes f)= Q(g,f)(\varphi (x)). \end{aligned}$$

The DOFS $(\mathcal {L}_{\operatorname {st.epi}}, \mathcal {R}_{\operatorname {mono}})_\text {restr}$ and $(\mathcal {L}_{\operatorname {st.epi}}, \mathcal {R}_{\operatorname {mono}})_\text {extn}$ both differ from the DOFS $(\mathbb {L}_{\operatorname {epi}}, \mathbb {R}_{\operatorname {mono}})$. By Theorem 5.7 there exist pseudo-morphisms of DOFS, $(\mathbb {L}_{\operatorname {epi}}, \mathbb {R}_{\operatorname {mono}}) \rightarrow (\mathcal {L}_{\operatorname {st.epi}}, \mathcal {R}_{\operatorname {mono}})_\text {restr}$ and $(\mathcal {L}_{\operatorname {st.epi}}, \mathcal {R}_{\operatorname {mono}})_\text {extn} \rightarrow (\mathbb {L}_{\operatorname {epi}}, \mathbb {R}_{\operatorname {mono}})$. Explicitly, the globular double cells that define these pseudo-morphisms are

**Figure:**
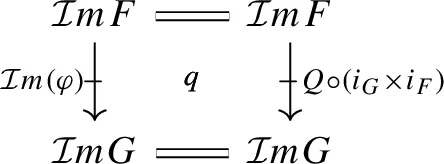

and

**Figure:**
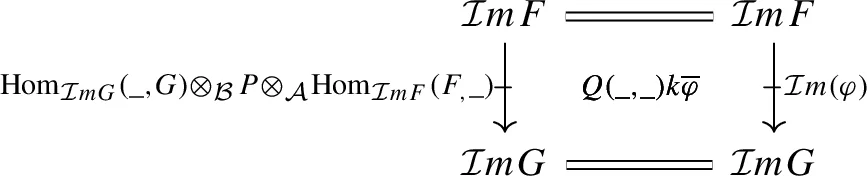

respectively. Here, the natural transformation $Q(\_,\_)k\overline{\varphi }$ is defined analogously to $Q(\_,\_)\varphi$ above.

#### Example 5.14

*(Quantale-valued relations,* *Q*- $\mathbb {R}\textrm{el}$ *)* Let *Q* be a unital quantale. A *Q-valued relation* between sets *X* and *Y* is a function $R:X \times Y \rightarrow Q$. Such relations may be composed; given , there exists a *Q*-valued relation , given by

$$\begin{aligned} (R \otimes S)(x,z) := \bigvee _{y \in Y} R(x,y) \odot S(y,z) \end{aligned}$$

where ⊙ denotes multiplication in *Q*. There is a double category *Q*- $\mathbb {R}\textrm{el}$ whose objects are sets, arrows are functions, and proarrows are *Q*-relations, with vertical composition given as above. A cell

**Figure:**
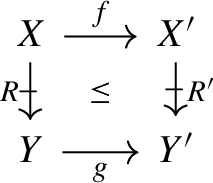

exists when $R\left(x,y\right)\leq R^{\prime }\left(f\left(x\right),g\left(y\right)\right)$ for all $$x \in X, y \in Y$$. If the inequality is in fact an equality for all x∈X and y∈Y, we indicate it by writing an equality symbol inside the cell. The unit proarrow associated to a set *X* is the function $$1:X\times X\rightarrow Q$$ that sends the diagonal to the top element 1∈Q and all other pairs to the bottom element 0∈Q. The unit cell associated to an arrow $f:X\to X^{\prime }$ is the cell

**Figure:**
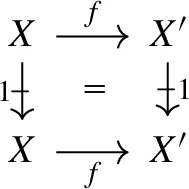

This double category *Q*- $\mathbb {R}\textrm{el}$ is fibrant. The restriction and extension cells are of the form

**Figure:**
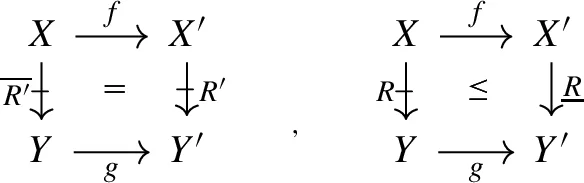

respectively. Here, $\overline{R'}:=R'\circ (f\times g)$ and $$\underline{R}(x',y'):= \bigvee _{\begin{array}{c} f(x)=x' \\ g(y)=y' \end{array}} R(x,y)$$.

Consider the surjective-injective orthogonal factorization system on the category of sets. Let $\mathcal {L}$ denote the class of surjective functions and $\mathcal {R}$ the class of injective functions. By Proposition 5.5, there exist two DOFSs in *Q*- $\mathbb {R}\textrm{el}$, namely $(\mathcal {L},\mathcal {R})_\text {restr}$ and $(\mathcal {L},\mathcal {R})_\text {extn}$ . These two DOFS provide distinct ways to factor a cell:

**Figure:**
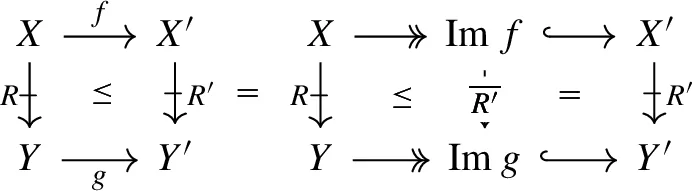

and

**Figure:**
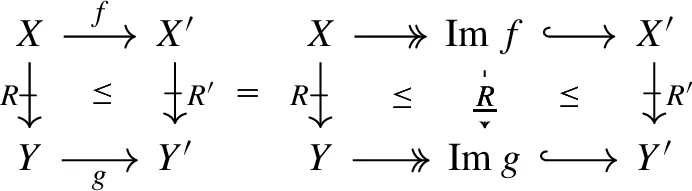

respectively. We verify how these two factorization systems are related. For $a\in \operatorname {Im}f$ and $b\in \operatorname {Im}g$, we calculate:

$$\begin{aligned} \underline{R}(a,b)= &   \bigvee _{\begin{array}{c} f(x)=a \\ g(y)=b \end{array}}R(x,y)\\\le &   \bigvee _{\begin{array}{c} f(x)=a \\ g(y)=b \end{array}}R'(f(x),g(y))\\= &   R'(a,b)\\= &   \overline{R'}(a,b). \end{aligned}$$

So we obtain a pseudo-morphism of DOFSs extending the epi-mono factorization system $(\mathcal {L},\mathcal {R})$ on , $(\mathcal {L},\mathcal {R})_{\operatorname {restr}}\rightarrow (\mathcal {L},\mathcal {R})_{\operatorname {extn}}$.

In the particular case $Q = \{0, 1\}$ we recover the double category $\mathbb {R}\textrm{el}(\textrm{Set})$ from Example 3.8, and the DOFS $(\mathcal {L},\mathcal {R})_\text {extn}$ coincides with the DOFS given in that example.

#### Example 5.15

*(The double category* $\mathbb {S}\textrm{pan}(\mathcal {C})$ *)* Let $\mathcal {C}$ be a category with pullbacks and an OFS $(\mathcal {L},\mathcal {R})$. In this example we want to expand on the work in Example 3.7. Let $Q:\mathcal {C}^\rightarrow \rightarrow \mathcal {C}$ be the functor that represents the OFS $(\mathcal {L},\mathcal {R})$. Write $f=r_f\circ \ell _f$ for the chosen factorizations of the arrows in $\mathcal {C}$. Then the factorization of a double cell in the DOFS of Example 3.7 is given by:

**Figure:**
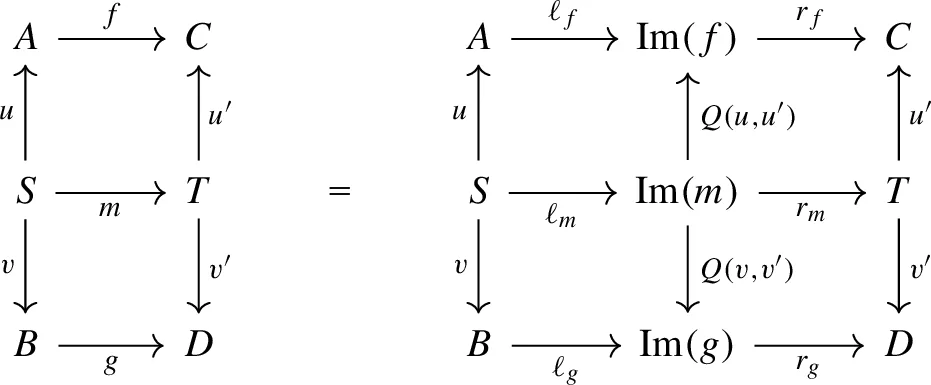

Extensions in $\mathbb {S}\textrm{pan}(\mathcal {C})$ are double cells of the form:

**Figure:**
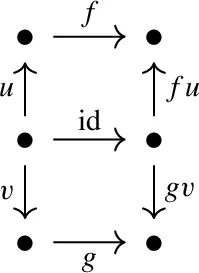

Hence, the factorization of cells in $(\mathcal {L},\mathcal {R})_{\operatorname {extn}}$ is given by:

**Figure:**
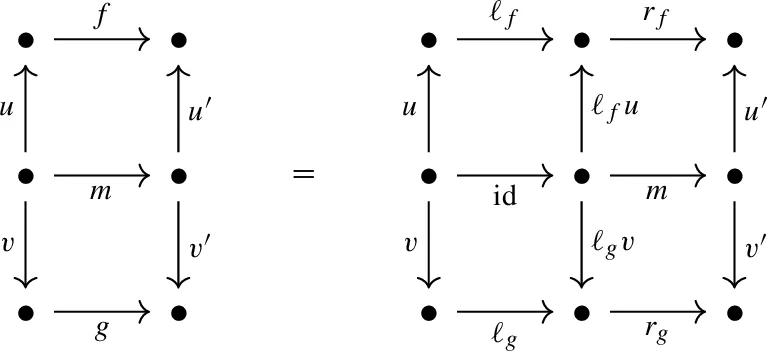

Because $\mathcal {C}$ has pullbacks, it also has restrictions, defined as:

**Figure:**
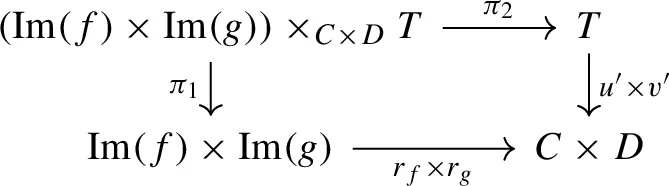

We will also write $(r_f\times r_g)^*(T)$ for $(\operatorname {Im}(f)\times \operatorname {Im}(g))\times _{C\times D}T$. This gives us the restriction cell

**Figure:**
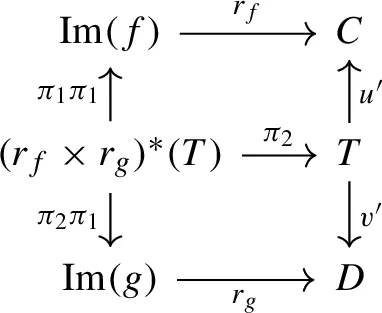

and the corresponding factorization of the double cell is given by:

**Figure:**
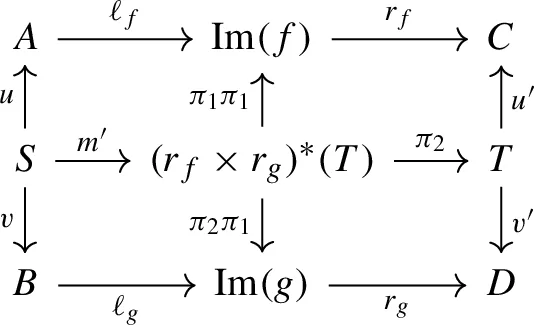

where $m'=((\ell _fu,\ell _gv),m)$ is the unique arrow into the pullback.

In the following diagram, we see how our original DOFS from Example 3.7 sits in between the extension and restriction systems:
8where $r_m'=((Q(u,u'),Q(v,v')),r_m)$ is the unique arrow into the pullback.

We will continue this example in Example 6.15 of the next section where we will add one more factorization system on $\mathbb {S}\textrm{pan}(\mathcal {C})$ that extends the epi-mono factorization system on .

## Double Factorization Systems and Double Fibrations

We study the interaction between double fibrations, introduced in [8], and our double orthogonal factorization systems. We generalize some results that are well-known in the ordinary case to the double categorical context and we present some examples. In particular, we show that both pseudo DOFSs and lax DOFSs can be lifted along double fibrations.

We start by recalling the following folklore result for which we could not find an appropriate reference. The construction in the proof will be used in proving the results for DOFS and double fibrations that follow.

### Proposition 6.1

Let $P:\mathcal {E} \rightarrow \mathcal {B}$ be an (ordinary) cloven Grothendieck fibration and let $(\mathcal {L}, \mathcal {R})$ be an orthogonal factorization system on $\mathcal {B}$. Then $(\mathcal {L}, \mathcal {R})$ lifts to an orthogonal factorization system $(\mathcal {L}^{P}, \mathcal {R}^{P})$ on $\mathcal {E}$, taking $\mathcal {L}^{P}$ to be the collection of morphisms over morphisms in $\mathcal {L}$ and $\mathcal {R}^{P}$ to be the collection of cartesian morphisms over morphisms in $\mathcal {R}$.

### Proof

Let f:x→y be a morphism in $\mathcal {E}$ and let $P(f)=r_{P(f)}\circ \ell _{P(f)}$ be the chosen factorization in $\mathcal {B}$. Then $f=\operatorname {cart}(r_{P(f)}, y) \circ g$, where $\operatorname {cart}(r_{P(f)}, y)$ is the cartesian lifting of $r_{P(f)}$ with codomain *y* given by the cleavage, and *g* is the unique morphism induced by the cartesian property of this lifting as in the following diagram; i.e., $P(g)=\ell _{P(f)}$.

**Figure:**
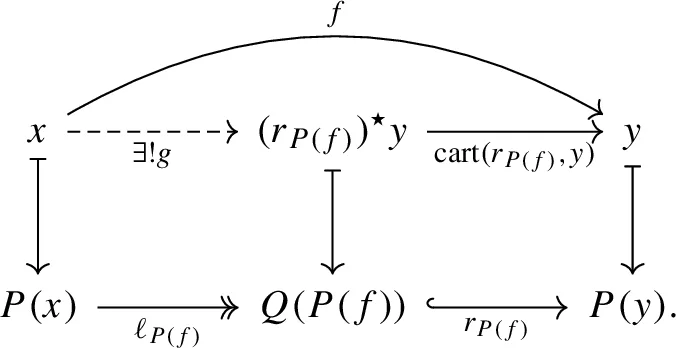

So *f* can be factorized as a composite of a morphism over a morphism in $\mathcal {L}$ followed by a morphism that is cartesian over a morphism in $\mathcal {R}$ (and the cleavage gives us the chosen factorization for *f*).

Moreover, using the fact that the morphisms in $\mathcal {R}^P$ are cartesian, it is straightforward to prove that the required lifting properties hold and hence $(\mathcal {L}^{P}, \mathcal {R}^{P})$ is an orthogonal factorization system on $\mathcal {E}$. □

### Remark 6.2

Note that in the proof of Proposition 6.1 we provided a lifting of the factorization system on $\mathcal {B}$ to one on $\mathcal {E}$ that makes the fibration *P* a strict morphism of OFSs.

We want to generalize the previous result of lifting of OFSs along fibrations to the double categorical context. Double fibrations have been introduced and studied in [8].

We recall that a double fibration $P:\mathbb {E}\rightarrow \mathbb {B}$ between pseudo double categories is a pseudo-category in the category  of fibrations. This amounts to a double functor $P:\mathbb {E}\rightarrow \mathbb {B}$ between pseudo double categories:
9such that $P_0$ and $P_1$ are cloven fibrations, $s_{\mathbb {E}}$ and $t_{\mathbb {E}}$ are cleavage preserving, and $y_{\mathbb {E}}$ and $\otimes _{\mathbb {E}}$ preserve cartesian morphisms. Note that the notion of double fibration as internal pseudo-category in the category of fibrations does not entirely line up with our notion of DOFS, because we have chosen to assume that the units functor is a strict morphism of DOFSs whereas it is not required to preserve cleavages in the notion of double fibration (it is taken to be among the looser class of morphisms). So, in order to obtain a lifting result, we have two options: either we put an extra assumption on the double fibration or we remove the strictness requirement on the units functor for DOFSs. We present the result for both cases.

### Theorem 6.3

Let $P:\mathbb {E}\rightarrow \mathbb {B}$ be a double fibration for which the units functor $y_{\mathbb {E}}$ is cleavage preserving, and let $(\mathbb {L},\mathbb {R})$ be a pseudo (resp. lax, colax) double orthogonal factorization system on $\mathbb {B}$. Then $(\mathbb {L},\mathbb {R})$ lifts to a pseudo (resp. lax, colax) double orthogonal factorization system $(\mathbb {L}^P,\mathbb {R}^P)$ on $\mathbb {E}$, taking $(\mathcal {L}^P_0, \mathcal {R}^P_0):=(\mathcal {L}_0^{P_0},\mathcal {R}_0^{P_0})$ and $(\mathcal {L}^P_1, \mathcal {R}^P_1):=(\mathcal {L}_1^{P_1},\mathcal {R}_1^{P_1})$ (in the notation of Proposition 6.1).

### Proof

Assume that $(\mathbb {L},\mathbb {R})$ is a double orthogonal factorization system that is either lax, colax or pseudo. We need to prove that $((\mathcal {E}_0, (\mathcal {L}_0^{P},\mathcal {R}_0^{P})), (\mathcal {E}_1, (\mathcal {L}_1^{P},\mathcal {R}_1^{P})))$ is a pseudo-category in the corresponding category of OFSs with strict source, target and identity arrows. We see immediately that Proposition 6.1 and Remark 6.2 imply that $(\mathcal {L}_0^{P},\mathcal {R}_0^{P})$ and $(\mathcal {L}_1^{P},\mathcal {R}_1^{P})$ are orthogonal factorization systems on $\mathcal {E}_0$ and $\mathcal {E}_1$ respectively such that $P_0$ and $P_1$ are strict morphisms between categories with orthogonal factorization systems. We will show below that $s_{\mathbb {E}}$, $t_{\mathbb {E}}$ and $y_{\mathbb {E}}$ are strict morphisms between categories with the lifted factorization systems and the chosen factorizations from the proof of Proposition 6.1. It follows then, thanks to Proposition 2.15, that the required pullbacks in  lift automatically to pullbacks in the appropriate categories of OFSs. We will end the proof by showing that if $\otimes _{\mathbb {B}}$ is a pseudo (resp. lax, colax) morphism of OFSs, so is $\otimes _{\mathbb {E}}$.

We now check that $s_{\mathbb {E}}$, $t_{\mathbb {E}}$ and $y_{\mathbb {E}}$ preserve the chosen factorizations. Let α be a cell in $\mathbb {E}$,

**Figure:**
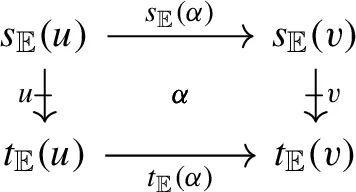

The factorization of $P_1(\alpha )$ is
10So the factorization of α is

**Figure:**
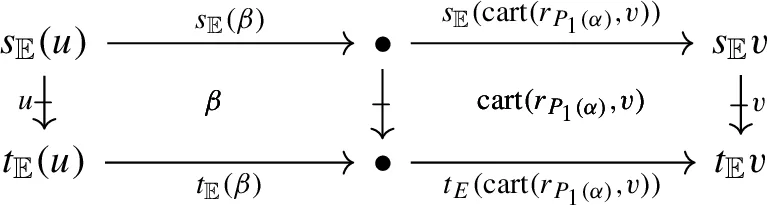

where $\operatorname {cart}(r_{P_1(\alpha )},v)$ is the chosen cartesian lifting of $r_{P_1(\alpha )}$ and β is the unique arrow over $\ell _{P_1(\alpha )}$ so that the composition gives α. We want to show that ${s_{\mathbb {E}}(\operatorname {cart}(r_{P_1(\alpha )},v))}\circ {s_{\mathbb {E}}(\beta )}$ is the chosen factorization in $\mathcal {E}_0$ of $s_{\mathbb {E}}(\alpha )$ and similarly for $t_{\mathbb {E}}(\alpha )$.

Since $s_{\mathbb {B}}$ and $t_{\mathbb {B}}$ are strict morphisms of OFSs, (10) is equal to the diagram

**Figure:**
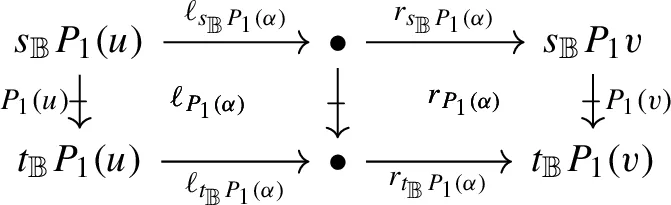

Since $P_0s_{\mathbb {E}}(\alpha )=s_{\mathbb {E}}P_1(\alpha )$ and $P_0t_{\mathbb {E}}(\alpha )=t_{\mathbb {E}}P_1(\alpha )$ and $s_{\mathbb {E}}$ is cleavage preserving as map between fibrations, we have that

$$\begin{aligned} r_{s_{\mathbb {E}}(\alpha )}= &   \operatorname {cart}(r_{P_0s_{\mathbb {E}}(\alpha )},s_{\mathbb {E}}(v))\\= &   \operatorname {cart}(r_{s_{\mathbb {B}}P_1(\alpha )},s_{\mathbb {E}}(v))\\= &   s_{\mathbb {E}}\operatorname {cart}(r_{P_1(\alpha )},v) \end{aligned}$$

By uniqueness of factorizations through cartesian arrows and the fact that $s_{\mathbb {E}}(\beta )$ fits we find that

$$\begin{aligned} \ell _{s_{\mathbb {E}}(\alpha )}=s_{\mathbb {E}}(\beta ) \end{aligned}$$

We conclude that $s_{\mathbb {E}}$ is a strict map of DOFSs. The proof for $t_{\mathbb {E}}$ and $y_{\mathbb {E}}$ goes similarly, using that $t_{\mathbb {E}}$ and $y_{\mathbb {E}}$ are cleavage preserving and that $t_{\mathbb {B}}$ and $y_{\mathbb {B}}$ are strict maps of DOFSs.

We observe that $s_{\mathbb {E}}, t_{\mathbb {E}}$ and $y_{\mathbb {E}}$ preserve both the left and the right class of arrows. Indeed, they preserve cartesian morphisms by definition of double fibration and the fact that the images of morphisms in the left and right class of the total category are over morphisms in the left and right class in the base category is guaranteed by the fact that $s_{\mathbb {B}}, t_{\mathbb {B}}$ and $y_{\mathbb {B}}$ preserve morphisms in the left and right class and by the commutativity of diagram (9). For example, given a double cell $\alpha \in \mathcal {L}_1^{P}$, we have $P_0(s_{\mathbb {E}}(\alpha ))= s_{\mathbb {B}}(P_1(\alpha ))$ and $s_{\mathbb {B}}(P_1(\alpha ))\in \mathcal {L}_0$ since $P_1(\alpha )\in \mathcal {L}_1$ and $s_{\mathbb {B}}$ preserves the left class.

Furthermore, when $\otimes _{\mathbb {B}}$ preserves the right class (i.e., the DOFS on the base of the double fibration is lax), the fact that $\otimes _{\mathbb {E}}$ preserves cartesian arrows gives us that $\otimes _{\mathbb {E}}$ also preserves the right class. Hence, $(\mathbb {L}^P,\mathbb {R}^P)$ is also a lax double orthogonal factorization system.

When $\otimes _{\mathbb {B}}$ preserves the left class (i.e., the DOFS on the base of the double fibration is colax), it follows immediately that $\otimes _{\mathbb {E}}$ does so as well, since the left class in $\mathbb {E}$ consists just of those cells the lie over cells in the left class in $\mathbb {B}$. Hence, $(\mathbb {L}^P,\mathbb {R}^P)$ is also a colax double orthogonal factorization system.

Finally, if $\otimes _\mathbb {B}$ preserves both classes (i.e., the DOFS on the base of the double fibration is pseudo), the arguments just given for the lax and colax cases both apply and we see that $\otimes _{\mathbb {E}}$ preserves both classes: the lifted DOFS on $\mathbb {E}$ is pseudo. □

### Corollary 6.4

Let $P:\mathbb {E}\rightarrow \mathbb {B}$ be a double fibration for which the units functor $y_{\mathbb {E}}$ is cleavage preserving. Then $\mathbb {E}$ has the pseudo DOFS

$$\begin{aligned} ((\text {pseudo-vertical}, \text {cartesian}), (\text {pseudo-vertical}, \text {cartesian})), \end{aligned}$$

where an arrow is pseudo-vertical if *P* sends it to an isomorphism.

### Proof

By Proposition 6.3 we can lift the trivial double orthogonal factorization system $((\text {isomorphisms}, \text {all morphisms}), (\text {isomorphisms}, \text {all morphisms}))$ on $\mathbb {B}$ to a double orthogonal factorization system on $\mathbb {E}$. By construction, the double orthogonal factorization system obtained this way has left classes given by pseudo-vertical morphisms and right classes given by cartesian morphisms. □

### Remark 6.5

If we remove the condition that $y_{\mathbb {E}}$ be cleavage preserving in the double fibration and we require the units functor of the DOFS on $\mathbb {B}$ to be merely a pseudo-morphism of OFSs, the construction in the proof of Theorem 6.3 still works and yields a DOFS of the same type on $\mathbb {E}$: the units functor will still preserve the left and right classes but it may not preserve the chosen factorizations.

### Remark 6.6

If $\otimes _{\mathbb {E}}$ in Theorem 6.3 is cleavage preserving, then a strict DOFS on $\mathbb {B}$ will also lift to a strict DOFS on $\mathbb {E}$.

By duality we obtain the analogous results for opfibrations.

### Proposition 6.7

Let $P:\mathcal {E} \rightarrow \mathcal {B}$ be an (ordinary) cloven Grothendieck opfibration and let $(\mathcal {L}, \mathcal {R})$ be an orthogonal factorization system on $\mathcal {B}$. Then $(\mathcal {L}, \mathcal {R})$ lifts to an orthogonal factorization system $(\mathcal {L}^{P}, \mathcal {R}^{P})$ on $\mathcal {E}$, taking $\mathcal {L}^{P}$ to be the collection of cocartesian morphisms over morphisms in $\mathcal {L}$ and $\mathcal {R}^{P}$ to be the collection of morphisms over morphisms in $\mathcal {R}$.

### Proof

Dual to the proof of Proposition 6.1. □

### Theorem 6.8

Let $P: \mathbb {E}\rightarrow \mathbb {B}$ be a double opfibration for which the units functor $y_{\mathbb {E}}$ is cleavage preserving, and let $(\mathbb {L},\mathbb {R})$ be a pseudo (resp. lax, colax) DOFS on $\mathbb {B}$. Then $(\mathbb {L},\mathbb {R})$ lifts to a pseudo (resp. lax, colax) DOFS $(\mathbb {L}^P,\mathbb {R}^P)$ on $\mathbb {E}$, taking $(\mathcal {L}^P_0, \mathcal {R}^P_0):=(\mathcal {L}_0^{P_0},\mathcal {R}_0^{P_0})$ and $(\mathcal {L}_1^{P}, \mathcal {R}_1^{P}):=(\mathcal {L}_1^{P_1},\mathcal {R}_1^{P_1})$ (in the notation of Proposition 6.7).

### Proof

Dual to the proof of Theorem 6.3. □

### Corollary 6.9

Let $P:\mathbb {E}\rightarrow \mathbb {B}$ be a double opfibration such that the units functor $y_{\mathbb {E}}$ is cleavage preserving. Then $\mathbb {E}$ has a pseudo DOFS

$$\begin{aligned} (( \text {cocartesian},\text {pseudo-vertical} \,), ( \text {cocartesian},\text {pseudo-vertical}\, )). \end{aligned}$$

### Proof

Dual to Corollary 6.4. □

In order to make a connection with the results in the previous section, notice that if $P:\mathbb {E}\rightarrow \mathbb {B}$ is a double fibration then a cartesian cell over a restriction cell is also a restriction cell; in particular, if $\mathbb {B}$ is fibrant, so is $\mathbb {E}$. The following result follows immediately.

### Proposition 6.10

The lifting of a restriction factorization system along a double fibration is a restriction factorization system.

We now present some applications of the results in this section.

### Example 6.11

*(Double Elements Construction)* Following [8], we can associate to any lax double pseudo-functor  (with $\mathbb {D}$ a pseudo double category) its double elements construction $\Pi :\mathbb {E}\textrm{l}(F) \rightarrow \mathbb {D}$. Recall that $\Pi _0$ is the (ordinary) elements construction of , while $\Pi _1$ is the elements construction of the composite
where $\operatorname {apx}$ is the apex functor that sends any span to its middle object. Explicitly, by [8, Theorem 3.46], the pseudo double category $\mathbb {E}\textrm{l}(F)$ is given as follows:

- objects: pairs (*C*, *X*) with $C\in \mathcal {D}_0$ and X∈F(C);
- arrows: pairs $(f,\overline{f}):(C,X) \rightarrow (D,Y)$ with f:C→D in $\mathbb {D}$ and $\overline{f}:X \rightarrow f^{\star }Y$ in *F*(*C*);
- proarrows: pairs  with  in $\mathbb {D}$ and $\overline{m} \in F(m)$, such that $D_m (\overline{m})=X$ and $U_m (\overline{m})=Y$ where the span

**Figure:**
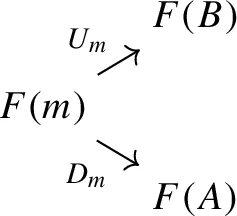

is the image of the proarrow *m* under *F*;
- double cells: pairs $(\theta , \overline{\theta })$ displayed

**Figure:**
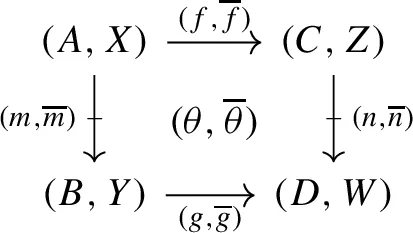

with

**Figure:**
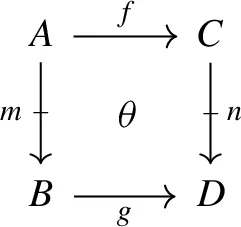

a double cell in $\mathbb {D}$ and $\overline{\theta }:\overline{m} \rightarrow \theta ^{\star }\overline{n}$ an arrow of *F*(*m*) such that $D_m (\overline{\theta }) =\overline{f}$ and $U_m(\overline{\theta }) =\overline{g}$.

Corollary 6.4 yields the DOFS, ((pseudo-vertical, cartesian), (pseudo-vertical, cartesian)), on $\mathbb {E}\textrm{l}(F)$, by lifting the trivial DOFS on $\mathbb {D}$ along Π. In terms of the explicit description of the double elements construction, we obtain the following factorizations. By the proof of Proposition 6.1, an arrow $(f,\overline{f}):(C,X) \rightarrow (D,Y)$ in $\mathbb {E}\textrm{l}(F)$ factorizes as the composite
since $(f,\operatorname {id})$ is the chosen cartesian lifting of *f* to *Y* along the fibration $\Pi _0:\mathbb {E}\textrm{l}(F)_0 \rightarrow \mathbb {D}_0$ and the arrow $(\operatorname {id}, \overline{f})$ is the vertical arrow induced by the cartesian property of $(f,\operatorname {id})$. A double cell

**Figure:**
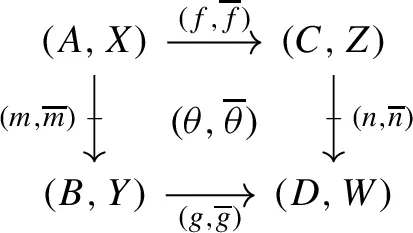

then factorizes as the composite

**Figure:**
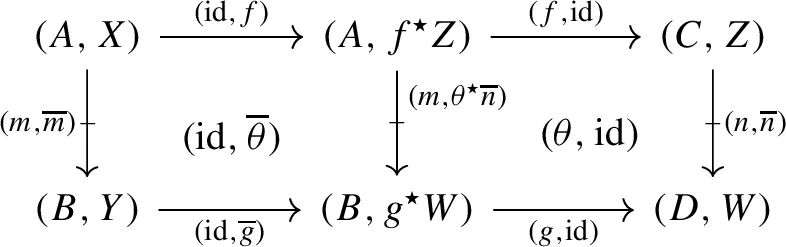

where the double cell $(\theta , \operatorname {id})$ is the chosen cartesian lifting of θ along $\Pi _1:\mathbb {E}\textrm{l}(F)_1 \rightarrow \mathcal {D}_1$ and the vertical double cell $(\operatorname {id}, \overline{\theta })$ is the one induced by cartesian property of $(\theta , \operatorname {id})$.

### Example 6.12

*(Domain Double Fibration)* Consider the domain double fibration

$$\begin{aligned} \operatorname {dom}:\mathbb {D}^{\rightarrow } \rightarrow \mathbb {D}\end{aligned}$$

that sends:

- an arrow  to *A*;
- a commutative square  to ;
- a double cell  to
- a cube  to

Given a double orthogonal factorization system $(\mathbb {L},\mathbb {R})$ on $\mathbb {D}$, Theorem 6.3 lets us lift it to a double orthogonal factorization system $(\mathbb {L}^{\operatorname {dom}},\mathbb {R}^{\operatorname {dom}})$.

In particular, since a square
11is cartesian if and only if *v* is an isomorphism, we obtain that $\mathcal {R}^{\operatorname {dom}}_0$ is the collection of commutative squares with top morphism in $\mathcal {R}_0$ and bottom morphism an isomorphism while $\mathcal {L}^{\operatorname {dom}}_0$ is the collection of commutative squares with top morphism in $\mathcal {L}_0$. Moreover, the square in diagram (11) factorizes as the composite

**Figure:**
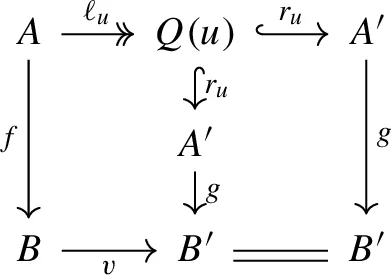

Analogously, a cube
12is cartesian if and only if the cell τ is horizontally invertible and thus $\mathcal {R}^{\operatorname {dom}}_1$ is the collection of cubes with left face in $\mathcal {R}_1$ and right face horizontally invertible, while $\mathcal {L}^{\operatorname {dom}}_1$ is the collection of cubes with left face in $\mathcal {L}_1$. Moreover, a factorization of the cube of diagram (12) is given by the composite:

**Figure:**
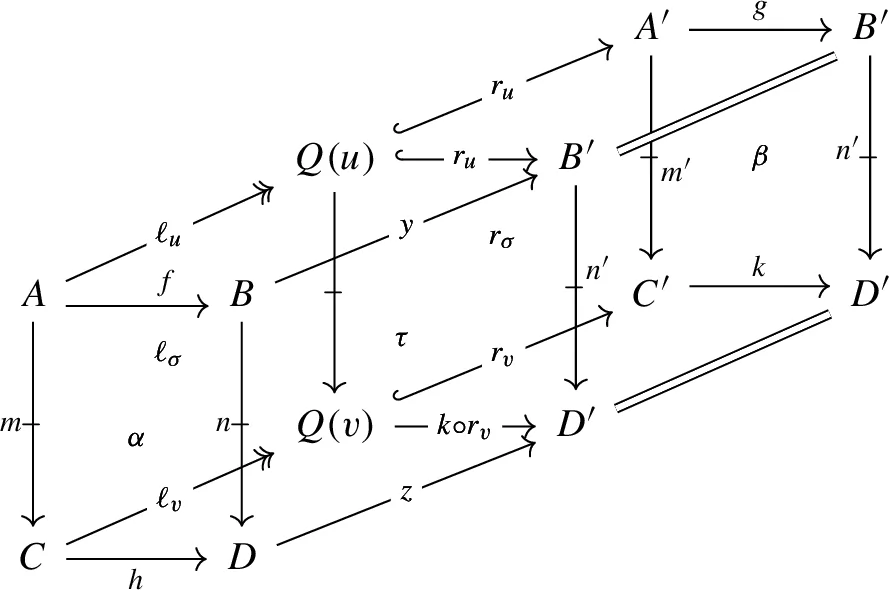

Furthermore, we notice that if $(\mathbb {L},\mathbb {R})$ is the trivial double orthogonal factorization system, the obtained factorizations coincide with the factorization system of the free algebra for the squaring monad (see Construction 4.6).

### Example 6.13

*(Codomain Double Opfibration)* Dual to Example 6.12, we can lift a double orthogonal factorization system $(\mathbb {L},\mathbb {R})$ on $\mathbb {D}$ along the double opfibration

$$\begin{aligned} \operatorname {cod}:\mathbb {D}^{\rightarrow } \rightarrow \mathbb {D}\end{aligned}$$

defined dually to $\operatorname {dom}:\mathbb {D}^{\rightarrow } \rightarrow \mathbb {D}$. A square is cocartesian exactly when its top morphism is an isomorphism. So, by Theorem 6.8, $\mathcal {L}^{\operatorname {cod}}_0$ is the collection of commutative squares with bottom morphism in $\mathcal {L}_0$ and top morphism an isomorphism while $\mathcal {R}^{\operatorname {cod}}_0$ is the collection of commutative squares with bottom morphism in $\mathcal {R}_0$. And analogously for double cells. The obtained factorizations of squares and cubes in $\mathbb {D}^{\rightarrow }$ are dual to the ones described in Example 6.12.

### Example 6.14

*(Codomain Double Fibration)* Let $\mathbb {D}$ be a pseudo-category in the 2-category of finitely-complete categories with chosen finite limits, limit-preserving functors (but not necessarily the chosen limits) and natural transformations. Assume also that source, target and identity functors preserve the chosen finite limits and the composition functor just preserves them up to isomorphism.

Under these hypotheses, the codomain double functor $\operatorname {cod}:\mathbb {D}^{\rightarrow } \rightarrow \mathbb {D}$ has a structure of double fibration as well, with cartesian liftings given by pullbacks (see Proposition 2.29 of [8]). Cartesian squares are exactly pullback squares and cartesian cubes are pullback squares of double cells (composed horizontally) in $\mathcal {D}_1$.

Given a DOFS $(\mathbb {L},\mathbb {R})$ on $\mathbb {D}$, its lifting along the double fibration $\operatorname {cod}$ produces a double orthogonal factorization system on $\mathbb {D}^{\rightarrow }$ as follows. The left class of squares consists of the squares with bottom morphism in $\mathcal {L}_0$, while the right class is the collection of pullback squares with bottom morphism in $\mathcal {R}_0$. And analogously for cubes, considering pullbacks in $\mathcal {D}_1$. The square

**Figure:**
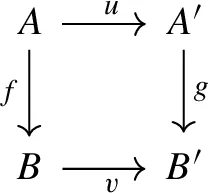

factorizes as the composite

**Figure:**
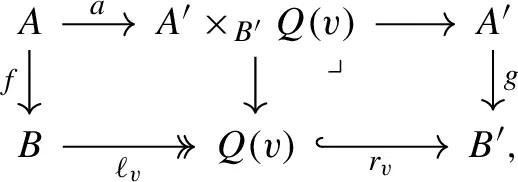

where *a* is the unique morphism induced by the universal property of the pullback $A'\times _{B'} Q(v)$ starting from the pair $(u,\ell _v \circ f)$. Factorizations of cubes are given analogously.

### Example 6.15

*(Image Double Fibration)* Consider the image double functor $\operatorname {Im}:\mathbb {S}\textrm{pan}\rightarrow \mathbb {R}\textrm{el}$ such that  is the identity and $\operatorname {Im}_1:\mathbb {S}\textrm{pan}_1 \rightarrow \mathbb {R}\textrm{el}_1$ sends a span  to the image $\operatorname {Im}(u,v)$ of  (seen as a relation) and a morphism of spans
13to the morphism of relations

**Figure:**
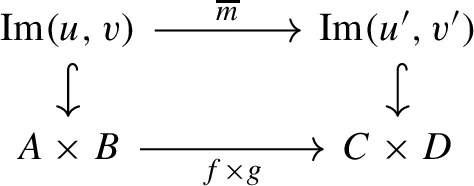

where $\overline{m}:\operatorname {Im}(u,v) \rightarrow \operatorname {Im}(u',v')$ is the function defined by,

$$\begin{aligned} (u(s), v(s))\mapsto (u'(m(s)), v'(m(s))). \end{aligned}$$

The double functor $\operatorname {Im}$ is a double fibration (Example 2.25, [8]). A morphism of spans (*f*, *m*, *g*) as in (13) is cartesian precisely when the square

**Figure:**
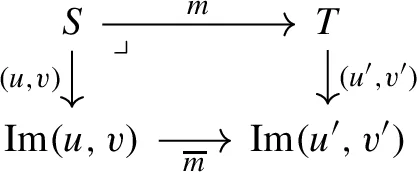

is a pullback. We can now consider two distinct double orthogonal factorization systems on $\mathbb {R}\textrm{el}$: the one defined by extensions and the one defined by restrictions (as in Example 5.14). The one defined by extensions was also described in detail in Example 3.8. Lifting this DOFS along the image double fibration, we obtain the following DOFS on $\mathbb {S}\textrm{pan}$. The left and right classes of arrows are given by surjective and injective functions as expected. The left class of double cells $\mathcal {L}_1^{\operatorname {Im}}$ is given by morphisms of spans (*f*, *m*, *g*) such that *f* and *g* are surjective and $\overline{m}$ is surjective, while the right class $\mathcal {R}_1^{\operatorname {Im}}$ is given by those morphisms of spans (*f*, *m*, *g*) such that *f* and *g* are injective, $\overline{m}$ is injective and (*f*, *m*, *g*) is cartesian. We can now compare this DOFS on $\mathbb {S}\textrm{pan}$ with the one described in Example 3.7. Since the surjectivity of *m* implies the surjectivity of $\overline{m}$ (but the two are not equivalent) we have that $\mathcal {L}_1^{\operatorname {Im}}$ strictly contains the left class of double cells of Example 3.7. On the other hand, as expected, since the injectivity of $\overline{m}$ implies the injectivity of *m*, we have that $\mathcal {R}_1^{\operatorname {Im}}$ is strictly contained in the right class of double cells described in Example 3.7. To obtain the morphisms between this DOFS on $\mathbb {S}\textrm{pan}$ and the system described in Example 3.7 as double factorization systems over a common factorization system on the arrows, we spell out the chosen factorizations of the cells in $\mathbb {S}\textrm{pan}$ with this system. We will denote this factorization by
14To determine the central object of this diagram and the arrows in and out of it, let  be the epi-mono factorization of $\overline{m}$. Then define $\mathcal {I}m(S)$ as the pullback in the following diagram,

**Figure:**
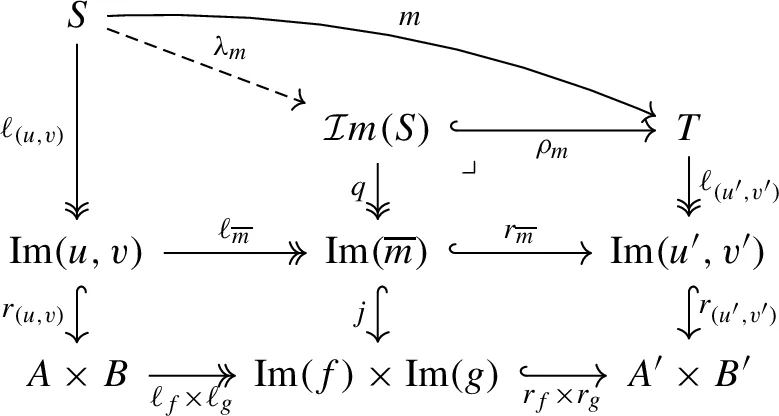

We will write $(q_1,q_2):\mathcal {I}m(S)\rightarrow \operatorname {Im}(f)\times \operatorname {Im}(g)$ for the composition j∘q. To compare the factorization $m=r_m\circ \ell _m$ (the chosen epi-mono factorization from Example 3.7) with $m=\rho _m\circ \lambda _m$, take the chosen epi-mono factorization of $\lambda _m$, $\lambda _m=\lambda '_m\circ \ell _m.$ This induces a morphism from this lifted system to the system from Example 3.7 given by the cells

**Figure:**
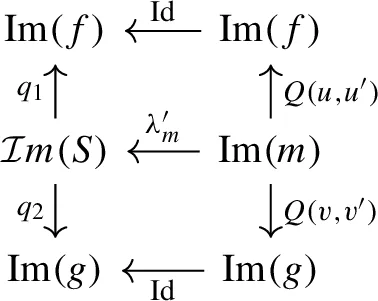

Furthermore, write $((q_1,q_2),\rho _m):\mathcal {I}m(S)\rightarrow (r_f\times r_g)^*(T)$ for the unique map into the pullback. By Proposition 6.10, the lifting of the restriction factorization system on $\mathbb {R}\textrm{el}$ lifts to the restriction factorization system on $\mathbb {S}\textrm{pan}$, so this does not give us anything new. We conclude that we have found four DOFSs on $\mathbb {S}\textrm{pan}$ that lift the epi-mono factorization system on , with morphisms between them represented by the double cells in the following diagram (this extends the diagram (8) from Example 5.15):

**Figure:**
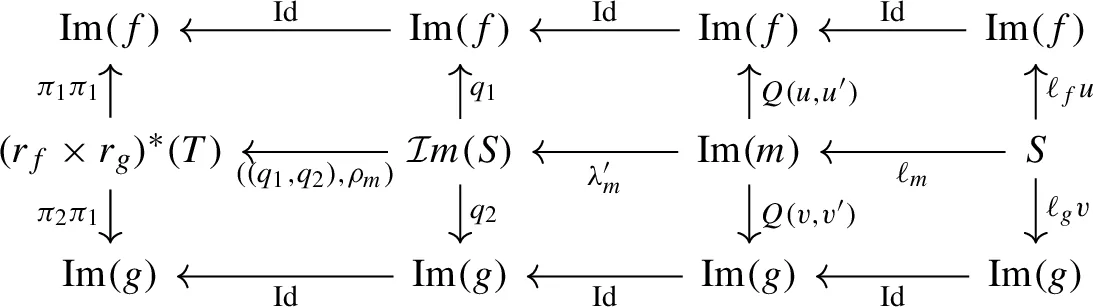

## Data Availability

No datasets were generated or analysed during the current study.
